# A survey of researchers’ attitudes to preregistration in animal research reveals multiple perceived barriers to adoption

**DOI:** 10.1371/journal.pbio.3003511

**Published:** 2026-07-28

**Authors:** Cristina Priboi, Boris Mayer, Evie Vergauwe, Bernice S. Elger, Hanno Würbel

**Affiliations:** 1 Veterinary Public Health Institute, University of Bern, Bern, Switzerland; 2 Institute of Psychology, University of Bern, Bern, Switzerland; 3 Faculty of Psychology and Educational Sciences, University of Geneva, Geneva, Switzerland; 4 Institute for Biomedical Ethics, University of Basel, Basel, Switzerland; Universidade Federal de Santa Catarina, BRAZIL

## Abstract

Preregistration is arguably one of the most promising and potentially impactful Open Science practices. Defined as the a priori registration of study designs and analysis plans, preregistration has long been established as standard practice in clinical human research and is increasingly taken up in other fields of science. Despite growing evidence suggesting that preregistration can mitigate questionable research practices, and the existence of two platforms targeting animal studies, preregistration has remained uncommon in animal research. In light of a “reproducibility crisis” and calls for more transparency and rigor also in animal research, preregistration represents a potentially promising step forward. However, implementing such policies without understanding their impact carries potential risks. It is, therefore, essential to uncover animal researchers’ perspectives on the strengths, weaknesses, opportunities, and threats of preregistration before advancing its implementation. Here, we addressed this need as part of a comprehensive feasibility project on preregistration of animal experiments in Switzerland, with the aims to: (1) assess animal researchers’ experiences with preregistration; (2) examine their attitudes, subjective norms, perceived behavioral control, intentions, motivations, and perceived obstacles regarding preregistration; (3) explore associations between these psychosocial constructs and relevant background characteristics; (4) identify perceived facilitators and barriers to preregistration; and (5) summarize researchers’ suggestions for improving preregistration. To this end, we conducted a preregistered, cross-sectional online survey among all registered study directors of ongoing animal experiments in Switzerland. Of 1,385 invited study directors, 418 completed the survey (30.2% return rate; 41% female; age *M* = 47.1, *SD* = 9.52). Of these, only 10% had preregistered studies before participating in the survey and 39.2% had never even heard of preregistration. Bureaucratic burden (77.6%), time costs (71.4%), and low flexibility (65.7%) were the most common reported barriers to preregistration. On average, participants reported rather unfavorable attitudes towards preregistration, negative subjective norms, relatively low perceived behavioral control, weak intention, and limited motivation to preregister, along with high perceived obstacles. Participants who had never preregistered a study, as well as those with more research experience, had more negative scores on all assessed psychosocial constructs related to preregistration. Our findings offer guidance on promising measures to enhance acceptance of preregistration among animal researchers, including raising awareness, offering education and training, and facilitating the procedure of preregistration.

## Abstract 2

Preregistration is a promising and potentially impactful Open Science practice, but remains uncommon in animal research. This survey of animal researchers’ experiences, attitudes and barriers toward preregistration reveals that despite recognized benefits, preregistration is hindered by perceived bureaucracy, time costs and inflexibility.

## Introduction

The scientific community is facing a profound methodological reformation towards Open Science in response to the so-called “reproducibility crisis”. Institutions, funders and journals are implementing policies to enhance rigor, transparency and reproducibility, with the goal of increasing credibility and trust in science. Preregistration is arguably one of the most promising and potentially impactful tools for improving transparency and scientific rigor [1–3]. Defined as the registration of hypotheses, study designs and analysis plans in independent open repositories before data collection or analysis [1,2,4,5], preregistration may reduce questionable research practices such as publication bias, selective reporting, *p*-hacking, and HARKing, which may help improve the reproducibility of research findings [1–4,6–9]. Although the effects of preregistration have only recently begun to be studied systematically and robust evidence is still scarce, available research from different disciplines provides direct or indirect support for a potential positive impact of preregistration on the reproducibility of research findings [10,11]. Preregistered studies reported fewer statistically significant results, found smaller effect sizes, and described methods in more detail compared to studies that had not been preregistered [6,8,12–17].

The inability to replicate published results, e.g., in cancer research [18], and the low rates of translating preclinical findings to clinical studies [19,20], have led to questions about the rigor and reproducibility of animal research [18,21–23]. This threatens public support for the responsible use of animals in research, which depends not only on the implementation of the 3Rs (replace, reduce, refine [24]) but also on the assumption that such practices will lead to important contributions to science, medical advances, or nature conservation. It has been shown that study protocols and results from many research projects using animals never get published or published in full [25–28]. Furthermore, substantial deficiencies in the use and reporting of measures against risks of bias have been documented [29,30], including in an exemplary study conducted in Switzerland [31,32]. Apart from increasing public doubts about animal research, such questionable research practices can slow down scientific progress, waste animal lives for inconclusive research, and expose participants in clinical trials to unnecessary risks [33–35].

Although study registries tailored to animal research have been established [4,5], the animal research community appears to meet preregistration with reservations [35]. This may be due to a number of reasons, including researchers’ concerns that preregistration would increase administrative burden, that it would stifle creativity and discovery, that others might steal their ideas, or that they would become targets of animal rights activists [17,36–38]. In addition, researchers may not be aware of the possibilities, functioning, and benefits of preregistration [35]. Nevertheless, policies regarding preregistration are already shifting with key stakeholders adopting preregistration standards for animal studies. A report by the Advisory Committee to the Director (ACD) working group on enhancing rigor, transparency, and translatability in animal research commissioned by the US National Institutes of Health has recommended preregistration for animal studies [39], an increasing number of journals are adopting preregistration badges as part of their publication policies [1,8], and a recent call has been made to implement preclinical study preregistration in the animal research community [38].

Despite the growing popularity of preregistration, the relevance, applicability, and implementation of preregistration have not yet been properly analyzed for animal research. There is a risk of changing policies too far or prematurely before the potential costs and benefits for innovation and research quality, and the practical implications of preregistration have been understood by all parties involved. There is, therefore, an urgent need to evaluate the perceived strengths, weaknesses, opportunities, and threats of preregistration in animal research. As part of a project to address these different aspects, we conducted the present survey among all animal study directors registered in Switzerland. The study was informed by Spitzer and Mueller [40], who had investigated attitudes and experiences with preregistration in psychology researchers. Building on their framework, the specific aims of our study were to:

1. assess the experience of animal researchers in Switzerland with study preregistration,
2. examine several psychosocial constructs related to preregistration, including attitudes, subjective norms, perceived behavioral control, intentions, motivations, and perceived obstacles,
3. explore associations between these six psychosocial constructs and background characteristics, such as animal researchers’ preregistration experience, animal research experience, gender, field of animal research, and organization of employment,
4. identify perceived barriers and facilitators to preregistration from the perspective of Swiss animal researchers, and
5. summarize participants’ suggestions for improving the practice of preregistration in Switzerland.

We reasoned that achieving these aims is important in view of providing guidance on promising measures to enhance acceptance of preregistration among animal researchers and facilitate its implementation.

## Materials and methods

The aims, design and analysis plan of this study were preregistered during data collection on the Open Science Framework on June 25, 2024 (https://doi.org/10.17605/OSF.IO/CAUFW). All deviations from the preregistered plan are described following Willroth and Atherton [41] and can be found in the supplementary material (S1 Table).

### Study design

This cross-sectional study received ethical approval from the Ethical Commission of the Faculty of Human Sciences at the University of Bern, Switzerland (Nr. 2024-03-06). All participants provided written informed consent prior to participation.

### Sample

The study sample included researchers accredited as “study directors” of animal experiments under Swiss law. In Switzerland, all animal experiments must be authorized by the competent authority (usually the cantonal veterinary office), and applications for a license to conduct an animal experiment must be submitted through the federal platform animex-ch (https://www.blv.admin.ch/blv/en/home/tiere/tierversuche/forschende/animex-ch.html). According to Swiss animal welfare legislation (TSchV Art. 131), study directors are responsible for designing and conducting animal experiments in compliance with the national and cantonal legal requirements. To qualify as a study director, researchers need to hold a biomedical university degree, have at least three years of experience with animal experimentation, and complete specific additional education and training. Although the role of “study director” is a legal designation, it largely overlaps with the role of “principal investigator”. Our decision to sample participants for this study from the population of study directors was based on the fact that they are usually the ones who decide whether or not to preregister a study. Since other personnel involved in animal experiments (e.g., animal care staff, attending veterinarians, technicians, and students) might have somewhat different views, our findings do not necessarily generalize to all animal researchers. Only researchers who held an active animal research license and were registered as study directors or deputy study directors at the time of recruitment were eligible to participate.

### Procedure and data collection

Data collection was conducted with the support of the Federal Food Safety and Veterinary Office (FSVO) in Switzerland and took place between 29.05.2024 and 26.06.2024. The FSVO, which holds contact information for all registered study directors and deputy study directors in the country, sent an official invitation email with the survey link to all eligible study directors. Two further reminders were sent by the FSVO, seven and 14 days after the initial invitation email. Additionally, one more reminder was issued at the institutional level by the Animal Welfare Officers (AWO). In Switzerland, every institution conducting animal research is obliged by law to have one or more AWO, whose role, as defined by the Animal Welfare Ordinance, is to ensure that applications for animal experiments are complete and coherent, particularly regarding the justification of the experiment’s indispensability, defined endpoints and harm-reduction measures, and the harm–benefit analysis. Our study was discussed within the national AWO network, and all AWO agreed to disseminate an additional reminder at the institutional level within their respective institutions. We provided them with a standard text for their email reminder to ensure consistent wording across institutions. We did not verify whether all AWO complied with this agreement, but we had no reason to doubt it. We, therefore, consider the risk of systematic bias introduced by this reminder to be negligible.

### Questionnaires

Participants completed an online survey in English that included both closed-ended and open-ended items. The items were adapted from the questionnaire developed by Spitzer and Mueller [40] and modified to align with the field of animal research and the study’s research questions. An overview of the specific changes can be consulted in the supplementary material (S1 File). The online survey collected information related to study preregistration, such as participants’ experience with preregistration, their attitudes, subjective norms, perceived behavioral control, intentions, motivations, obstacles, facilitators, barriers, and suggestions for improving preregistration, as well as socio-demographic characteristics.

#### Socio-demographic characteristics.

The following demographic variables were collected from the sample: age, gender, years registered as study directors, years of animal research experience, academic age, educational attainment, seniority level, organization of employment, and field of animal research.

#### General experiences.

Between three and seven closed-ended items, depending on whether participants had ever preregistered a study or not, were used to assess participants’ general practices and their experiences with study preregistration (S1 File).

#### Psychosocial constructs.

The survey included six Preregistration Scales, each evaluating one of the following six psychosocial constructs related to preregistration: attitudes (23 items); subjective norms (7 items); perceived behavioral control (7 items); intentions (3 items); motivations (10 items); and obstacles (10 items). These constructs were primarily derived from the Theory of Planned Behavior (attitudes, subjective norms, perceived behavioral control, and intentions), supplemented by closely related motivational components (motivations and obstacles) [42–44].

- The attitudes scale measured participants’ evaluations of and opinions toward the concept of preregistration.
- The subjective norms scale explored the social pressure and expectations perceived by participants regarding preregistering their studies.
- The perceived behavioral control scale assessed participants’ judgment of their own ability and confidence to preregister studies.
- The intentions scale evaluated the determination to preregister future studies.
- The motivations scale focused on understanding the rationales behind researchers’ decisions to preregister or not preregister their studies.
- The obstacles scale measured challenges perceived by researchers in the preregistration process and was conceptualized as part of the psychosocial framework because obstacles capture researchers’ subjective evaluations rather than objective constraints.

A detailed description of the items for each scale is provided in the supplementary material (S1 File). All items were closed-ended and were assessed on a 7-point Likert scale ranging from 1 to 7. The values were re-coded from the original scale to a scale centered around 0 ranging from −3 to +3.

#### Barriers and facilitators.

The number and wording of items addressing barriers and facilitators of preregistration varied depending on whether participants had previously preregistered a study or not (S1 File). Barriers were assessed with one closed-ended item and up to four open-ended items *(“What do you perceive as drawbacks of preregistration?”; “What do you think would be the long-term negative consequences of mandatory preregistration?”; “What are the reasons for not preregistering your studies?”; “I am now less motivated to preregister than I was before because____”)*. Facilitators were examined exclusively with open-ended items (*“What do you perceive as benefits of preregistration?”; “What do you think would be the long-term benefits of mandatory preregistration?”; “I am now more motivated to preregister than I was before because____”*).

#### Suggestions.

The following open-ended items were used to collect suggestions for improving preregistration of animal experiments in Switzerland: *“We are interested in how we can improve various aspects of preregistration (e.g., templates, repositories, reviewing process, integrations in published articles, education, etc.). Do you have any suggestions? What do you think should be improved about preregistration?”; “What would make you (and perhaps other researchers) preregister more often?”; “Do you have any suggestions as to how to lower your (or other researchers’) negative perceptions of preregistration?”*.

### Data analysis

#### Sample description.

Descriptive statistics are reported to summarize the socio-demographic characteristics of the study participants. To assess comparability between participants and non-participants, we used an equivalence-based approach. Specifically, we examined whether 90% confidence intervals of effect sizes fell within predefined bounds of negligible effects, corresponding to a two one-sided tests (TOST) framework. For categorical variables, equivalence was defined as |*φ*| < 0.1, and for continuous variables as |*d*| < 0.2, reflecting conventional thresholds for small effects [45]. Confidence intervals entirely within these bounds were interpreted as evidence of practical equivalence. The following information regarding non-participants was provided by the FSVO: the total number of eligible study directors invited to participate, and, separately for participants and non-participants, their age (range, median, mean, and standard deviation), gender (number and percentage of female and male participants), and years since their registration as study directors (range, median, mean, and standard deviation). Due to differences in the operationalization of the gender variable and to ensure comparability with FSVO data, the survey responses were re-coded prior to analysis. The FSVO used two categories (“female” and “male”), while our survey included four options (“female”, “male”, “other”, and “prefer not to say”). For consistency, responses coded as “other” and “prefer not to say” were treated as missing in the comparative analysis. The values made available by the FSVO enabled comparisons between participants and non-participants with respect to age, gender, and years since registration as study directors.

#### General experiences (Aim 1).

Descriptive statistics were calculated and reported for each item assessing participants’ general experiences with preregistration. In addition, participants with and without preregistration experience were compared across all socio-demographic characteristics. Independent samples *t*-tests were used to analyze mean differences in continuous variables, while percentage differences in categorical variables were tested using Fisher’s Exact Test.

#### Psychosocial constructs (Aim 2).

***Psychometric quality:*** The items of the scales used in the survey were adapted from a questionnaire developed by Spitzer and Mueller [40], who reported high reliabilities for all six Preregistration Scales. However, it remained unclear whether these scales were truly unidimensional. Therefore, we performed principal component analyses (PCA) to explore the dimensional structure of the items within each of the six scales. To determine the appropriate number of components, we applied parallel analysis, using the mean eigenvalues of 1,000 randomly generated samples for comparison. In addition, we used a criterion based on the ratio of the first and second eigenvalue of the PCA (ratios > 3) [46], as well as omega hierarchical (ωH) from a bifactor model (values ≥ .70) [47–49], to assess essential unidimensionality (S2 File). Essential unidimensionality refers to the existence of a dominant general component with the simultaneous presence of subdimensions.

If more than one component was identified for a specific scale after applying these criteria, the extraction was followed by an oblique Oblimin-rotation to arrive at a simple structure even when components were correlated. Subsequently, we calculated McDonald’s omega to assess the internal consistency of each scale (or subscale—in case of more than one dimension obtained in the PCA for a specific scale). Item selection was based on factor loadings, corrected item-total correlations, and conceptual alignment.

***Overview of scales:*** Descriptive results are reported for each of the six Preregistration Scales (attitudes, subjective norms, perceived behavioral control, intentions, motivations, obstacles).

#### Association analysis (Aim 3).

A multivariate analysis of covariance (MANCOVA) was conducted to examine the overall associations of several predictors with the Preregistration Scales and potential Subscales, which served as the outcome variables. The predictors included in the multivariate model were: preregistration experience (“preregistered at least one study”, “never preregister a study”), animal research experience (in years), gender (“female”, “male”), field of animal research (“basic biological research”, “general biology”, “basic and experimental medical research”), and organization of employment (“academia”, “private sector”, “other”). Due to the very low frequency of responses in the gender categories “other” (*n* = 1) and “prefer not to say” (*n* = 14), these responses were re-coded as missing prior to analysis. Wilks’ Lambda was used to test the overall multivariate effects of the MANCOVA model for each predictor variable. Pillai’s Trace was additionally computed as a robustness check to verify the stability of the results, given its greater resistance to violations of MANCOVA assumptions.

If a significant multivariate effect was detected, follow-up univariate analyses of covariance (ANCOVAs) were performed for each outcome separately to identify which predictors were significantly associated with which outcome. Because the univariate follow-up involved testing the same predictor across multiple outcomes, we controlled the familywise error rate within each predictor by applying a Holm correction across the univariate *p*-values corresponding to that predictor. Partial eta-squared ($\eta_{p}^{\text{2}}$) was computed for each predictor within each outcome to estimate the effect sizes in the univariate models. Values of 0.01, 0.06, and 0.14 for $\eta_{p}^{\text{2}}$ were considered small, medium and large [50].

For categorical predictors with more than two levels (i.e., field of animal research and organization of employment), post-hoc pairwise comparisons were conducted only for those outcomes for which the predictor remained statistically significant after Holm correction. Post-hoc tests were based on estimated marginal means (adjusted for all other predictors in the model), with Tukey’s correction applied to pairwise comparisons within each outcome.

#### Thematic analysis (Aims 4 and 5).

The answers provided to the qualitative open-ended items were analyzed using inductive thematic analysis [51,52]. Initial coding was conducted by one researcher (CP), followed by discussing the resulting codes and preliminary categories with a second researcher (HW). Codes were subsequently grouped into higher-order categories reflecting facilitators, barriers, and suggestions for improving preregistration. Recurring themes were identified and described within each category. The coding framework was refined iteratively, and differences in interpretation between researchers were resolved by consensus. Example quotations are presented to illustrate each theme.

## Results

### Sample description

The FSVO identified 1,428 eligible study directors and invited them via email to take part in the online survey. Each study director was provided with a unique access code required to enter the survey. Forty-three emails bounced back, resulting in 1,385 successfully reached study directors. Of these, 744 individuals clicked on the survey link. However, 58 did not enter an access code, 59 entered an incorrect code, and 106 either failed to check the consent box or explicitly declined consent, preventing them from proceeding. Additionally, 17 access codes were used to complete the questionnaire twice, and 2 codes were used three times. In such cases, only the first completed attempt was kept, and subsequent entries were deleted. Three additional cases were removed at the request of the participants. After these exclusions, 497 valid entries remained. A further 17 cases were excluded due to over 90% missing data, and 62 for failing the attention check item (S1 Fig). A final sample of *N* = 418 participants was included in the analysis, resulting in a response rate of 30.2% among the reached population.

#### Socio-demographic characteristics.

Participants were on average 47 years old, and slightly less than half identified as female (Table 1). They had been registered as study directors for about 13 years and had worked in animal research for about 20 years. The majority were highly educated, with over 90% reporting a PhD, Dr. med. or higher, and 60% occupying senior positions such as group leader or professor. The predominant research field was basic and experimental medical research, and most participants were employed in academia.

**Table 1:** Sample description.

Characteristics	Participants (*n* = 418)	Non-participants (*n* = 1,010)	Total (*N* = 1,428)
**Age** ^**a**^			
*M (SD)*	47.1 (9.52)	47.9 (9.65)	47.8 (9.60)
*Mdn*	46.0	47.0	47.0
Range	24–84	27–94	24–94
*n*	418 (0 missing)	943 (67 missing)	1,361 (67 missing)
**Gender** ^**b**^			
Female	41.0% (170)	44.0% (276)	–
Male	55.4% (230)	56.0% (352)	–
Other	0.2% (1)	–	–
Prefer not to say	3.4% (14)	–	–
*n*	400 (18 missing)	628 (382 missing)	–
**Years registered as study director** ^**c**^			
*M (SD)*	12.6 (5.86)	12.8 (5.86)	12.8 (5.86)
*Mdn*	12.7	13.3	13.3
Range	0.2–28.4	0.3–26.2	0.2–28.4
*N*	418 (0 missing)	1,010 (0 missing)	1,428 (0 missing)
**Animal research experience**			
*M (SD)*	20.1 (8.83)	–	–
*Mdn*	19.0	–	–
Range	2–44	–	–
*n*	414 (4 missing)	–	–
**Academic age**			
*M (SD)*	17.3 (9.32)	–	–
*Mdn*	16.0	–	–
Range	0–40	–	–
*n*	408 (10 missing)		
**Educational attainment**			
Master’s degree	5.3% (22)	–	–
PhD/Dr. med.	51.6% (214)	–	–
Habilitation/professorship	41.9% (174)	–	–
Other	1.2% (5)	–	–
*n*	415 (3 missing)	–	–
**Seniority level**			
PhD student/technician	3.9% (16)	–	–
Postdoc./senior researcher	26.6% (110)	–	–
Lecturer	2.7% (11)	–	–
Group leader/Professor	60.0% (248)	–	–
Other	6.8% (28)	–	–
*n*	413 (5 missing)	–	–
**Field of animal research**			
Basic and experimental medical research^d^	72.6% (302)	–	–
General biology^e^	13.9% (58)	–	–
Basic biological research^f^	13.5% (56)	–	–
*n*	416 (2 missing)	–	–
**Organization of employment**			
Academia	77.6% (323)	–	–
Academia and other	2.1% (9)	–	–
Private sector	13.7% (57)	–	–
Governmental	2.6% (11)	–	–
Non-profit	3.8% (16)	–	–
*n*	416 (2 missing)	–	–

*Note. M*, mean; *SD*, standard deviation; *Mdn*, median; *n*, subgroup sample size; *N*, total sample size.

^a^The values for both participants and non-participants were provided by the Swiss Federal Food Safety and Veterinary Office (FSVO). FSVO calculated age using exact birthdates (day, month, year), whereas our survey asked only for the year of birth to protect confidentiality. Therefore, to ensure comparability, FSVO-provided age values are presented. The mean age of participants calculated from the survey data was nearly identical to the FSVO value (*M* = 46.9, *SD* = 9.20).

^b^The values for non-participants are FSVO-provided data, whereas those for participants are based on survey responses. This distinction arises because FSVO collected gender data using only two categories (female, male), while our survey allowed for four options (female, male, other, and prefer not to say).

^c^The values for both participants and non-participants are FSVO-provided data. They are reported instead of the survey data for participants because the FSVO dataset contained no missing values for this variable.

^d^Including following fields: Biomedical Engineering; Cancer Research; Cardiovascular Research; Endocrinology; Immunology; Medical Microbiology; Neuroscience; Nutrition and Metabolism; Pathology and/or Pathophysiology; Pharmacology; Physiology; Regenerative Medicine; Toxicology; Veterinary Medicine; Virology.

^e^Including following fields: Animal Breeding; Animal Nutrition; Animal Welfare; Ecology; Ethology; Evolution; Laboratory Animal Science; Wildlife Biology; Zoology.

^f^Including following fields: Biochemistry; Biophysics; Cell Biology; Cytology; Developmental Biology; Embryology; Epigenetics; Experimental Microbiology; Genetics; Molecular Biology; Radiobiology; Structural Biology.

To assess whether the two samples can be considered equivalent, we examined 90% confidence intervals (CIs) of effect sizes. For gender distribution, the 90% CI of the *φ*-coefficient (*φ* = −0.01, 90% CI [−0.07, 0.04]) fell entirely within the bounds of a small effect (|*φ*| < 0.1), indicating equivalence. Similarly, for age (*d* = −0.08, 90% CI [−0.18, 0.01]) and years registered as study director (*d* = −0.03, 90% CI [−0.13, 0.06]), the confidence intervals were fully contained within the range of negligible effects (|*d*| < 0.2). These findings suggest that any differences between participants and non-participants are unlikely to represent effective selection biases.

### General experiences (Aim 1)

Only 10.0% (*n* = 42) of the total sample reported having previously preregistered at least one study, while 90.0% (*n* = 376) had never done so (S2 Table). Overall, 39.2% (*n* = 147) of participants had never heard of preregistration prior to this survey, while 31.5% (*n* = 118) had read about it, and 27.2% (*n* = 102) had learned about it during further education (S2 Table). Institutional policies were most frequently chosen as influencing the decision whether or not to preregister (47.6%, *n* = 175), followed by funding guidelines (35.6%, *n* = 131), superior(s) (32.6%, *n* = 120), and journal guidelines (26.4%, *n* = 97). Notably, 28.0% of participants (*n* = 103) indicated that nobody has an influence on their decision (S2 Table). Participants with preregistration experience had a median of 3 preregistered studies (range 1–80), with an estimated median time of 11 hours per preregistered study (range 1–150) (S2 Table).

Comparing the socio-demographic characteristics of participants with and without preregistration experience revealed no statistically significant differences between these two groups (S3 Table).

### Psychosocial constructs (Aim 2)

#### Psychometric quality.

The dimensionality and internal consistency of all preregistration scales was assessed using PCA together with further criteria to assess essential unidimensionality, and McDonald’s omega. When necessary, items with poor psychometric properties (low factor loadings and/or low corrected item-total correlations) and weak conceptual alignment were removed, resulting in improved internal consistency and construct validity of each scale. Specifically, Items 9 and 21 were removed from the Attitudes Scale, and Item 2 was removed from the Subjective Norms Scale due to poor psychometric performance and weak conceptual alignment. No further items were removed from the remaining scales (S2 File). The Preregistration Scales for Attitudes, Subjective Norms, Intentions, and Motivations showed clear unidimensional structures and good to excellent reliability (*ω* = .84 to *ω* = .96). In contrast, the Perceived Behavioral Control Scale and the Obstacles Scale revealed a two-component structure and were therefore each divided into two subscales. The Perceived Behavioral Control Scale was separated into: the Perceived Behavioral Control – Resources Subscale (*ω* = .72), reflecting resources and support for preregistration from colleagues and supervisors; and the Perceived Behavioral Control – Knowledge Subscale (*ω* = .65), reflecting the knowledge required and authority to decide about preregistration. Similarly, the Obstacles Scale was structured into: the Practical Obstacles Subscale (ω = .84), reflecting perceived disadvantages in terms of time and effort; and the Competitive Obstacles Subscale (*ω* = .66), describing concerns about scooping and competitive challenges. For detailed results of these analyses, see S2 File.

#### Overview of scales.

Based on the psychometric analysis, the following Preregistration Scales and Subscales were used as outcomes in all subsequent analyses: attitudes scale (21 items), subjective norms scale (6 items), perceived behavioral control – resources subscale (4 items), perceived behavioral control – knowledge subscale (3 items), intentions scale (3 items), motivations scale (10 items), practical obstacles subscale (6 items), and competitive obstacles subscale (4 items).

Descriptive statistics of all Preregistration Scales and Subscales are presented in Table 2. Across the entire sample, participants reported slightly negative attitudes toward preregistration, moderately negative subjective norms, and slightly negative perceived behavioral control on both subscales. Similarly, both their intentions and motivations to preregister were slightly negative. In contrast, practical obstacles were perceived as moderately higher, and competitive obstacles slightly higher than neutral, indicating that many participants perceived substantial barriers to preregistration. However, on all scales and subscales, participants’ ratings ranged from very low (−2.2 to −3.0) to very high (+1.8 to +3.0) (Table 2).

**Table 2:** Overview preregistration scales.

Scales	Total participants (*N* = 418)
**Attitudes Scale**	
*M (SD)*	−0.9 (1.22)
*Mdn*	−0.8
Range	[−3.0; +2.2]
*N*	371 (47 missing)
**Subjective Norms Scale**	
*M (SD)*	−1.6 (1.00)
*Mdn*	−1.7
Range	[−3.0; +1.8]
*N*	353 (65 missing)
**Perceived Behavioral Control**	
*Resources Subscale*	
*M (SD)*	−0.7 (1.06)
*Mdn*	−0.8
Range	[−3.0; +2.2]
*N*	351 (67 missing)
*Knowledge Subscale*	
*M (SD)*	−0.4 (1.30)
*Mdn*	−0.8
Range	[−3.0; +3.0]
*N*	351 (67 missing)
**Intentions Scale**	
*M (SD)*	−0.8 (1.65)
*Mdn*	−0.7
Range	[−3.0; +3.0]
*N*	349 (69 missing)
**Motivations Scale**	
*M (SD)*	−0.8 (1.36)
*Mdn*	−0.8
Range	[−3.0; +2.2]
*N*	348 (70 missing)
**Obstacles**	
*Practical Obstacles Subscales*	
*M (SD)*	1.5 (1.04)
*Mdn*	1.7
Range	[−2.2; +3.0]
*N*	348 (70 missing)
*Competitive Obstacles Subscales*	
*M (SD)*	0.7 (1.05)
*Mdn*	0.8
Range	[−2.5; +3.0]
*N*	348 (70 missing)

*Note. M*, mean; *SD*, standard deviation; *Mdn*, median; *N*, total sample size.

### Association analysis (Aim 3)

**Fig 1:**
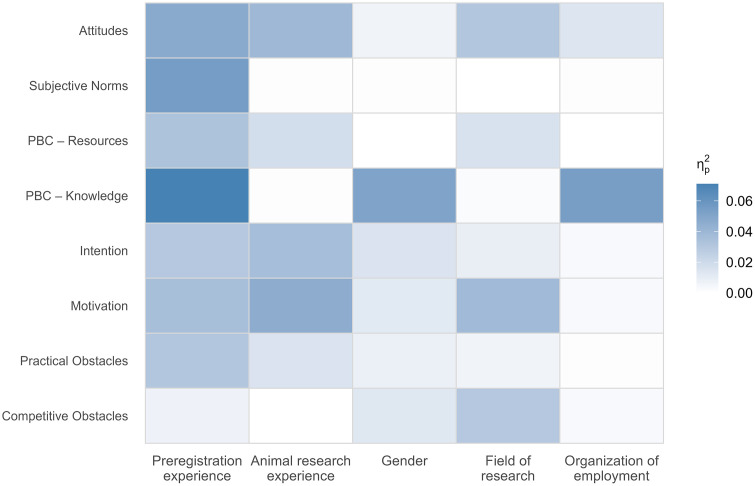
Strength of associations between predictors and outcomes. PBC, Perceived Behavioral Control. Cell color indicates partial $\eta_{p}^{\text{2}}$ estimates from univariate ANCOVAs adjusted for all other predictors in the model. The data and code required to generate this figure can be found at https://doi.org/10.48620/98470.

Analyses were performed on complete cases (*n* = 329). A MANCOVA was conducted to examine the associations of preregistration experience, animal research experience, gender, field of research, and organization of employment with the eight Preregistration Scales and Subscales. All predictors were entered simultaneously, so that each effect reflects the unique association of that predictor with the combined outcome variables, controlling for the other predictors. The multivariate model revealed statistically significant associations for all predictors, namely preregistration experience, animal research experience, gender, field of research, and organization of employment (S4 Table). Fig 1 provides an overview of the strength of these associations across predictors and outcomes based on eta-squared values from the follow-up univariate models. Preregistration experience was most strongly associated with Preregistration Scales and Subscales, followed by animal research experience and field of animal research, while organization of employment and gender showed comparatively weaker associations (Fig 1).

#### Preregistration experience.

**Fig 2:**
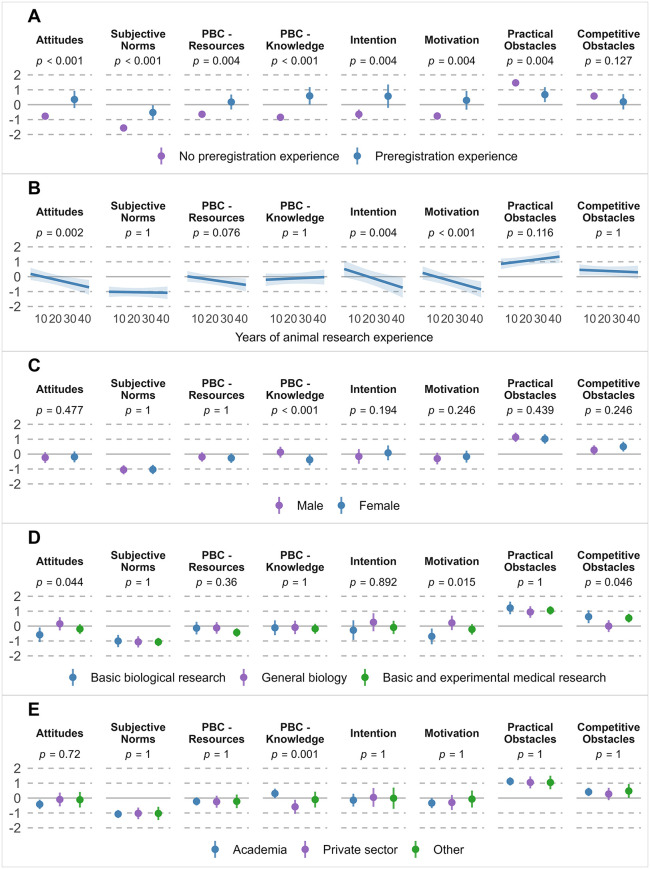
Associations across predictors and outcomes. **(A)** Adjusted means by preregistration experience. **(B)** Adjusted associations by years of animal research experience. **(C)** Adjusted means by gender. **(D)** Adjusted means by field of animal research. **(E)** Adjusted means by organization of employment**.** *Note.* PBC, Perceived Behavioral Control. Points in A, C, D, E represent adjusted estimated marginal means controlling for the remaining predictors and error bars indicate 95% confidence intervals. Lines in B represent adjusted predicted values across years of animal research experience, controlling for the remaining predictors, and shaded areas indicate 95% confidence intervals. The data and code required to generate these figures can be found at https://doi.org/10.48620/98470.

The univariate ANCOVAs (Holm-corrected) showed that participants with preregistration experience had more favorable attitudes, higher subjective norms, greater perceived behavioral control regarding both resources and knowledge, higher intentions, higher motivations, and fewer perceived practical obstacles than those without preregistration experience, while no significant difference between the two groups was found for perceived competitive obstacles (Fig 2A and S4 Table). The corresponding effect sizes were mostly small to medium in magnitude ($\eta_{p}^{\text{2}}\;\approx \;.\text{03}-.\text{07}$).

#### Animal research experience.

By contrast, greater experience in animal research was negatively associated with several Scales and Subscales in the univariate ANCOVAs, although effect sizes were rather small ($\eta_{p}^{\text{2}}\;\approx \;.\text{04}-.\text{05}$). Specifically, more years of research experience was associated with less favorable attitudes, lower intentions, and lower motivations to preregister (Fig 2B and S4 Table). No significant associations were observed for remaining Scales and Subscales (S4 Table).

#### Gender.

The univariate analyses further showed differences between genders, with women reporting moderately lower perceived behavioral control—knowledge compared to men, while no significant gender differences were detected for all other Scales and Subscales (Fig 2C and S4 Table). The corresponding effect size was in the small-to-moderate range ($\eta_{p}^{\text{2}}\;\approx \;.\text{05}$), indicating a modest difference rather than a pronounced gender effect (S4 Table).

#### Field of research.

Univariate ANCOVAs also revealed differences across participants’ field of animal research in attitudes, motivations, and competitive obstacles (Fig 2D and S4 Table). Post hoc Tukey pairwise comparisons showed that participants working in General Biology reported more positive attitudes towards preregistration, higher motivations to preregister, and lower perceived competitive obstacles compared to those in Basic Biological Research or Basic and Experimental Medical Research. Effect sizes were small ($\eta_{p}^{\text{2}}\;\approx \;.\text{03}-.\text{04}$), indicating modest differences, and no further significant differences were observed for the remaining outcomes (S4 Table).

#### Organization of employment.

With respect to the participants’ employer, a slight to moderate difference was found for perceived behavioral control—knowledge ($\eta_{p}^{\text{2}}\;\approx \;.\text{05}$) (Fig 2E and S4 Table). Post hoc Tukey pairwise comparisons revealed that participants working in academia rated their knowledge about preregistration higher than those working in the private sector. No additional significant associations were observed for the remaining Scales and Subscales (S4 Table).

### Thematic analysis (Aims 4 and 5)

A total of 334 participants responded to at least one open-ended item addressing barriers, facilitators, or suggestions for improving preregistration. Of these, 321 had no preregistration experience, while 13 had preregistered at least once prior to data collection. Given the small number of participants with preregistration experience, the uneven distribution of responses across participant characteristics, and the generally brief nature of the open-ended responses, subgroup analyses (e.g., by preregistration experience or other background characteristics) would not have been meaningful. Accordingly, the thematic analysis presented below primarily reflects the views of participants without preregistration experience. An overview of the qualitative codes, including the number of participants who mentioned each code at least once, is provided in S5–S7 Table.

#### Barriers (Aim 4).

**Fig 3:**
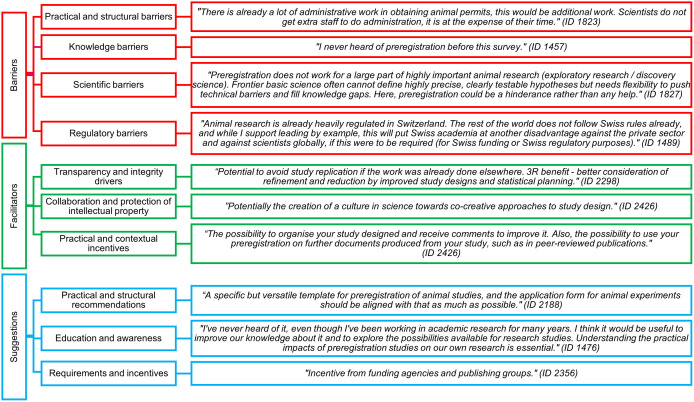
Thematic analysis.

Responses to open-ended questions on preregistration barriers were provided by up to 313 participants (S5 Table). The thematic analysis of these responses revealed four overarching themes: practical and structural barriers, knowledge barriers, scientific barriers, and regulatory barriers (Fig 3).

- Practical and structural barriers

Participants described both practical and structural barriers to preregistration of their studies. Regarding practical barriers, they identified bureaucratic burden (*n* = 107), the time needed for preregistration (*n* = 81), the costs and delays preregistration might entail (*n* = 26), and a lack of resources (*n* = 5). Concerns were also raised about the risk of being scooped (*n* = 72), confidentiality issues (*n* = 10), and worries about external complaints and harassment (*n* = 10), as well as incompatibility with industry practices and patenting (*n* = 5). Structural barriers included the lack of interest (*n* = 31), the absence of requirements from institutions, journals, or funders (*n* = 28), as well as a lack of incentives or feedback mechanisms related to preregistration (*n* = 8). In some cases, researchers reported limited support from co-authors and research partners for preregistration (*n* = 2), while others were not in the position to make the decision themselves (*n* = 8).

- Knowledge barriers

Many participants stated that they were not aware that preregistration even existed (*n* = 76), while some were just unfamiliar with the procedure, describing a lack of guidance or uncertainty about the steps involved and the appropriate platforms for their studies (*n* = 7). Others, however, expressed general skepticism about the potential benefits and overall impact of preregistration (*n* = 42).

- Scientific barriers

Many participants working in basic or experimental research questioned the suitability of their studies for preregistration based on the unpredictability and need for flexibility of their research (*n* = 74). Several of them emphasized the frequent need to deviate from initial methodological plans as part of the iterative nature of their research programs (*n* = 27), which for them seems incompatible with preregistration. Others raised more general concerns that preregistration limits flexibility (*n* = 48), compromises innovation (*n* = 20), and stifles creativity (*n* = 14).

- Regulatory barriers

A main issue under regulatory barriers was the country’s highly controlled research environment that limits animal research (*n* = 22) and reduces international competitiveness (*n* = 18). Others more generally stated the overregulation of animal research in Switzerland (*n* = 16), and some participants even expressed concerns that additional interventions, such as (mandatory) preregistration, could lead Swiss animal researchers to relocate to countries with more permissive regulations (*n* = 16).

- Quantitatively assessed barriers

**Fig 4:**
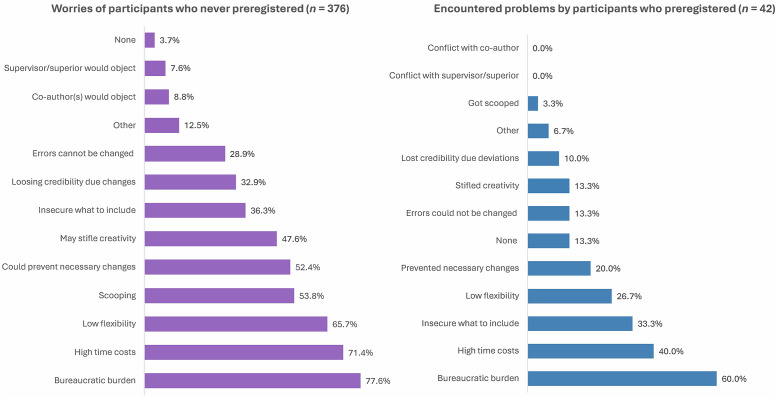
Barriers to preregistration. *n*, subgroup sample size. The data required to generate this figure can be found at https://doi.org/10.48620/98470.

Apart from the open-ended items, barriers to preregistration were also assessed using two closed-ended items: one administered to participants with and one to participants without preregistration experience. The responses to the closed-ended items reflected the patterns identified through thematic analysis. Participants who had never preregistered before chose bureaucratic burden (77.6%), time costs (71.4%), and low flexibility (65.7%) as the most common barriers to preregistration (Fig 4). Additionally, more than half also indicated concerns of being scooped (53.8%) and the inability to make necessary changes to their study protocol (52.4%) as important barriers (Fig 4). Similarly, the problems most frequently encountered by those with preregistration experience were bureaucratic burden (60%), time costs (40%), insecurity about what to include in the preregistration (33.3%), limited flexibility during analysis (26.7%), and being prevented from making necessary changes to the study protocol (20%) (Fig 4). Overall, the concerns expressed by participants without preregistration experience were mirrored by the problems reported by those with preregistration experience, but the rates of concerns by the inexperienced participants were much higher than the rates of problems encountered by the experienced participants.

#### Facilitators (Aim 4).

When asked about the benefits of preregistration, up to 140 participants described various advantages of the practice (S6 Table). These perceived benefits could serve as motivators for adopting preregistration in the future. Three main themes emerged from the thematic analysis: transparency and integrity drivers, collaboration and protection of intellectual property, and practical and contextual incentives (Fig 3). However, while some participants recognized these advantages, many saw no benefits at all (*n* = 55), or only for confirmatory studies (*n* = 12).

- Transparency and integrity drivers

Despite the mainly negative perception overall, some participants pointed out that preregistration could promote transparency in science (*n* = 30), increase credibility and trustworthiness (*n* = 7), and support the Open Science movement (*n* = 4). Others highlighted its role in reducing redundant parallel research (*n* = 10) and contributing to a better public understanding of research (*n* = 9).

Besides benefits to transparency and openness, participants mentioned the potential to promote research rigor and scientific integrity. Some argued that it could generally improve research quality (*n* = 19) or the reliability and reproducibility of research findings (*n* = 10). Others noted more specifically that it could lead to better methodological planning and study design (*n* = 15), improve statistical analysis (*n* = 5), or promote uniformity of experimental protocols (*n* = 7) and fewer deviations (*n* = 3). Others saw benefits in preventing questionable research practices (*n* = 8), such as HARKing (*n* = 5) and p-hacking (*n* = 4), or in reducing publication bias (*n* = 16) or researcher bias (*n* = 2). Furthermore, a few participants mentioned better consideration of the 3Rs of animal use (*n* = 8).

- Collaboration and protection of intellectual property

Participants also described collaboration-related benefits of preregistration. Some pointed out that preregistration may encourage scientific feedback and exchange (*n* = 16), while others described its potential to foster collaborations and synergies (*n* = 2). In addition, preregistration was seen by some as providing a clear track record of the studies, which could help protect researchers’ intellectual property (*n* = 6).

- Practical and contextual incentives

Finally, participants mentioned some practical and contextual incentives related to preregistration that might facilitate its future use. Some highlighted the improved time and project management, which they attributed to better study planning required for preregistration (*n* = 19). A few noted that it may help with drafting papers and even speed up the journal review process, as parts of the manuscript are already written during the preregistration phase (*n* = 2).

#### Suggestions (Aim 5).

Up to 137 participants responded to the open-ended items regarding suggestions for improving preregistration (S7 Table). The following overarching themes emerged from the thematic analysis: practical and structural recommendations, education and awareness, and incentives (Fig 3).

- Practical and structural recommendations

Participants offered a wide range of practical and structural recommendations to adapt the preregistration process to the Swiss context. They suggested the implementation of an easy-to-use tool with a clear, yet flexible, template (*n* = 34) and emphasized the need to make changes after preregistration (*n* = 18). Some proposed linking this tool to the legal authorization procedure for animal studies (*n* = 29). Strong confidentiality measures were also recommended (*n* = 16) and extending the embargo period to more than five years (*n* = 4). In addition, participants asked for institutional support and resources, such as dedicated staff and funding (*n* = 8), and highlighted the value of receiving expert feedback on the preregistered studies (*n* = 6). A few participants called for international rather than national implementation of preregistration (*n* = 3). Furthermore, several participants advocated for preregistration to remain voluntary in Switzerland (*n* = 11), and if made mandatory, to restrict it to confirmatory studies (*n* = 6).

- Education and awareness recommendations

To facilitate the adoption of preregistration in Switzerland, participants described a need for more knowledge and awareness (*n* = 27). Specifically, they requested information on where and how to preregister (*n* = 13), access to workshops, courses, or continued education options (*n* = 11), and concrete examples of preregistered studies (*n* = 1). Importantly, however, many participants emphasized the importance of proof that preregistration is beneficial across all types of research, including basic and exploratory studies (*n* = 34).

- Incentive recommendations

Principal investigators, institutions, funders, journals, and the scientific community were identified as essential in fostering the practice of preregistration (*n* = 28). Several participants noted that endorsement or requirement from these parties would legitimize preregistration in animal research. Participants also listed a series of incentives that could encourage researchers to preregister, including a faster authorization procedure for animal studies, benefits from funding organizations, or publishing advantages (*n* = 16).

## Discussion

With this survey, we aimed to evaluate animal researchers’ experience with, and perspectives on, preregistration in animal research. To this end, we assessed psychosocial constructs related to preregistration among animal researchers, using the population of animal study directors accredited in Switzerland as a study population. To our knowledge, this is the first study to examine psychosocial constructs related to preregistration among animal researchers. Previous work has examined preregistration in animal research through qualitative stakeholder analyses of implementation barriers [53] and survey-based studies of perceived strengths and weaknesses among animal researchers [37]. In contrast, the present study provides a structured assessment of psychosocial factors related to preregistration, informed by the Theory of Planned Behavior, by examining key components such as attitudes, perceived social norms, perceived behavioral control, and related barriers and experiences.

### General experiences

We found that almost half of the participants were unaware of preregistration, and only 10% had preregistered a study before. These results are in stark contrast to a recent survey on preregistration in psychology in Germany [40], where only 4% of participants had never heard of preregistration, and 62% had preregistered at least one study in the past. Similarly, a further survey conducted in the Netherlands reported that 43% of researchers across all research fields had prior experience with preregistration [54], while studies from behavioral and social sciences found rates of approximately 20% [55,56]. Although these differences may be partly explained by sampling biases inherent to the convenience samples of previous studies, our findings suggest that awareness of, and experience with, preregistration are rather limited among animal researchers.

Interestingly, motivation for first-time preregistration also differed across studies. In our sample, almost half of the participants indicated that they had preregistered because it was mandatory for a project, while only one-third was self-motivated. By comparison, in the German study on researchers in psychology, 49% had preregistered voluntarily, and only 13% reported having preregistered because it was mandatory. This suggests that enforcement, rather than an intrinsic motivation, led Swiss animal researchers to preregister. In line with this, nearly half of the participants indicated that institutional policies would influence their decision to preregister in the future. However, almost a third stated that nothing could affect their decision. Our findings therefore suggest that policy changes may hold the greatest potential in promoting preregistration among animal researchers, but the widespread resistance within the research community should be considered when developing and implementing new policies.

### Psychosocial constructs and associations

On average, participants showed negative perceptions on all psychosocial constructs assessed here. They exhibited rather negative attitudes toward preregistration, perceived social norms as discouraging preregistration, and indicated limited abilities for preregistration in terms of both resources and knowledge. Once again, our results differ from those described in other disciplines and countries, where preregistration was typically perceived and evaluated rather positively [40,56,57]. Furthermore, both the intention and motivation to preregister future studies were low in our sample, while obstacles to preregister were perceived as relatively high. These findings indicate that animal researchers are reluctant to engage in preregistration and perceive the barriers to do so as substantial. However, participants with prior preregistration experience held more positive views of preregistration compared to those who had never preregistered before, a pattern also observed in the field of psychology [40,57]. In contrast, the longer participants had worked in animal research, the more negative their perceptions of preregistration were. This is in line with evidence from behavioral science and psychology, where more experienced researchers showed greater reluctance toward preregistration [40,56]. Taken together, our findings reveal substantial resistance and little support for preregistration within the Swiss animal research community, particularly among researchers without preregistration experience and more senior researchers. Future efforts to promote preregistration should therefore tailor their approach to target these critical subgroups.

Although no significant associations of gender and organization of employment were observed for any other Scale or Subscale, they both differed in the researchers’ perceived ability to preregister. Thus, female researchers and participants employed in the private sector rated their knowledge required for preregistration lower than male researchers and those working in academia. These findings may suggest potential structural differences in animal researchers’ capacities to preregister, with women and industry researchers perceiving themselves less equipped to engage in preregistration. However, the gender difference could also reflect that men tend to overestimate their knowledge more often than women, suggesting that women’s lower ratings may indicate more accurate or honest self-evaluations rather than a true lack of capacity [58].

Lastly, the field of animal research in which participants were active was also associated with differences in perceptions of preregistration. Researchers from General Biology (including areas such as animal behavior, nutrition, ecology and zoology) reported more favorable perceptions of and less competitive obstacles regarding preregistration than biomedical researchers. Whether this indicates that General Biology is perceived as less competitive, resulting in less fear of being scooped compared to basic and applied biomedical research is not clear. However, disciplinary differences should be considered when allocating resources toward fostering preregistration among animal researchers.

### Barriers and facilitators

The most common practical and structural barriers identified by participants without preregistration experience included bureaucratic burden, excessive time investment, restricted flexibility, and challenges related to implementing necessary modifications to study plans. These findings are consistent with previous qualitative work identifying similar concerns among animal research stakeholders [53], as well as with evidence from psychology [40], where comparable barriers have been observed, suggesting that these issues are shared across disciplines. Notably, in our study, anticipated barriers among participants without preregistration experience exceeded the actual problems reported by those who had preregistered. This discrepancy indicates a gap between perceived and actual barriers to preregistration, likely stemming from assumptions rather than practical realities of preregistration. Moreover, several concerns reported by participants without preregistration experience reflect common misconceptions, such as the assumption that deviations from a preregistered study plan are not allowed or discouraged. In fact, preregistration has been described as a “plan, not a prison”, recognizing the need for deviations when they are justified and transparently reported [59]. Methodological guidance on when and how to deviate from preregistered study plans is available, demonstrating that deviations are part of preregistered research [41,60]. The same is true for the fear of being scooped. In fact, preregistration protects against scooping, as it provides a time stamp to a research idea much earlier in the process, and by putting the preregistered study plan under embargo until the findings have been published prevents scooping of research ideas. Such misconceptions are concerning, as they contribute to unfavorable attitudes towards preregistration and prevent researchers from engaging with it. Targeted education for animal scientists may help address some of these perceived barriers and correct misconceptions. At the same time, it is paramount to acknowledge the existing administrative burden and the regulatory pressure faced by the animal research community in Switzerland and elsewhere. Our analysis suggests that some animal researchers perceive preregistration as redundant given the existing authorization procedure for animal experimentation, and the majority expressed concerns about the additional administrative burden that preregistration would entail. This may also reflect the general frustration and pressure experienced by Swiss animal scientists with the current system, which may contribute to the negative attitudes towards preregistration as it is perceived as an additional administrative layer within an already very bureaucratic process. It is, therefore, important to emphasize that preregistration does not duplicate the authorization procedure but serves a complementary purpose. While the authorization procedure is legally mandated and serves ethical approval and implementation of the 3R principle, preregistration aims to foster transparency and scientific rigor of animal research. Before implementing new policies, authorities should be mindful of the research context and of the potential resistance such changes may raise.

In addition to practical and structural barriers, several participants indicated that preregistration is incompatible with the unpredictable, iterative nature of discovery-oriented, exploratory research. Although preregistration is arguably more straightforward in the case of a confirmatory study with a clearly defined hypothesis, it is another common misunderstanding that preregistration cannot be used for exploratory research. Exploratory research generates preliminary evidence and hypotheses that stimulate further avenues of research [61]. It is thus in the best interest of the scientific community that exploratory research is trustworthy and useful [62]. Again, this perceived barrier might be addressed by educating animal researchers about existing flexible preregistration templates (e.g., AsPredicted, OSF) that support preregistration of specific types of research.

Apart from the barriers to preregistration, participants also acknowledged a range of potential benefits, which may serve as motivators for its adoption. Participants saw value in preregistration for promoting transparency, improving study design and fostering scientific integrity. Some also noted its potential to mitigate publication bias, enhance research quality and support better consideration of animal use. Similar perceived benefits, particularly regarding transparency, research quality, and reduction of publication bias, have been highlighted in previous work in animal research [37,53]. Emphasizing the role of preregistration in addressing questionable research practices and the overall contribution to scientific rigor might help motivate researchers for preregistration.

Taken together, the barriers and facilitators as currently perceived by the animal research community highlight the complexity of implementing preregistration in animal research. Addressing misconceptions, emphasizing the benefits, and providing support for targeted problems may help reduce resistance and facilitate adoption of preregistration among animal researchers.

### Suggestions

Participants provided a wide range of recommendations for improving and facilitating preregistration. Among practical aspects, they highlighted the need for a clear and flexible template, strong confidentiality measures, additional funding, specialized support staff, and integration of preregistration with the authorization procedure for animal experimentation to avoid duplication and unnecessary bureaucratic burden. Moreover, participants stressed the importance of keeping preregistration voluntary. These suggestions underline the need to adjust policies and procedures of preregistration to the specific research context (e.g., the existing authorization procedure for animal experiments) to facilitate uptake among scientists.

Given the limited knowledge about preregistration within the animal research community, participants also emphasized the need to increase awareness and offer education and training. Similar to findings in behavioral science [56], participants expressed uncertainty about the benefits of preregistration and highlighted the importance of evidence demonstrating that preregistration improves scientific quality. In addition, participants indicated a preference for concrete incentives to encourage preregistration, such as preregistration accelerating the authorization procedure, funding benefits, and publishing advantages. Thus, besides offering appropriate education and training, as well as institutional support for preregistration, providing adequate infrastructure might be key for scaling up the use of preregistration in animal research. This could entail aligning the application form for the authorization of animal experiments with a preregistration template to avoid duplication and facilitate automatic transfer of relevant content to the preregistration template.

### Strengths and limitations

A major strength of this study lies in the clearly targeted study population and the high response rate, with nearly one-third of all accredited study directors of animal experiments in Switzerland participating in the survey. Unlike previous studies, which were typically based on convenience samples and thus less representative of their target populations, our survey reached the entire population of accredited study directors. Based on the available socio-demographic and professional characteristics, we did not detect any differences between study participants and non-participants that would indicate selection biases. Taken together, these aspects suggest that our study sample was representative of our target population. Nonetheless, because only a limited set of variables could be compared between participants and non-participants, some caution may be warranted when extending our findings to the overall population of animal study directors in Switzerland. Moreover, our decision to target study directors was based on the fact that they are the ones who decide whether or not to preregister a study, while other personnel involved in animal experiments (animal care staff, attending veterinarians, technicians, and students) may have different views but do not usually have the power to decide on whether or not to preregister a study. Therefore, our findings may not necessarily generalize to all personnel involved in animal research. However, given the international nature of the Swiss animal research community and the observation that research communities often vary more by field than by country, the patterns described in this study may be informative beyond the Swiss context. Particularly for countries with regulatory structures similar to those in Switzerland, such as the countries within the European Union, our findings may provide useful guidance for fostering preregistration in animal research. Another important strength of the study is its mixed-methods design. The use of both closed-ended and open-ended measurements allowed for a deeper and more nuanced understanding of the studied constructs, which should enhance the robustness of our findings.

Apart from these strengths, the following limitations of the study should be considered. First, despite the high response rate and the apparent similarity between participants and non-participants, our sample might still suffer from some selection bias. For example, individuals with particularly strong opinions about preregistration may have been more likely to participate in the study, leading to a skewed distribution of responses, towards both more negative and more favorable evaluations of preregistration. However, given that almost 40% of the participants had never heard of preregistration prior to the survey, it seems unlikely that such strong views were predominant, suggesting that selection bias may be limited. Second, despite being introduced to the definition of preregistration at the beginning of the survey, some participants may not have read or fully understood the concept and may have confused preregistration with the mandatory authorization procedure required to obtain a license for an animal experiment in Switzerland (animex-ch). Such misunderstanding would likely bias the results toward an overestimation of preregistration experience and further reduce the proportion of participants with actual preregistration experience in our sample. This is supported by requests received after survey completion from three participants who asked to have their data deleted upon realizing that they had confused the two concepts. Finally, we do not know whether participants with preregistration experience had preregistered preclinical animal studies or clinical human studies, which may again lower the proportion of those with actual preregistration experience in animal research. If preregistration experience was derived from clinical human research, these participants may not have encountered the specific challenges associated with preregistering animal studies. As a result, their responses may be more similar to those of participants who never preregistered, thereby attenuating observed differences between groups. As almost half of the participants indicated that their first preregistration was carried out because it was mandatory for a project, it is plausible that these cases refer to clinical human studies where preregistration is mandatory. Nevertheless, this may not compromise our conclusions, as the underlying concept and purpose of preregistration—namely transparency on the design, conduct, analysis, and reporting of research—is the same across both research domains.

### Conclusions

Preregistration is arguably among the most impactful practices promoted by the Open Science movement. While it has long been established as standard practice in human clinical trials [17] and is increasingly taken up in other fields of research [55], preregistration has not been widely adopted in animal research. Given the limited knowledge of preregistration among animal scientists in Switzerland, as revealed by this study, targeted educational initiatives that clarify the purpose of preregistration, address common misconceptions (e.g., regarding flexibility, deviations, and scooping), and provide guidance on how to preregister studies are necessary before the research community would consider adopting preregistration on a wider scale. The widespread skepticism and resistance among specific subgroups of researchers—particularly those with no prior preregistration experience, greater research seniority, and those working in biomedical research—highlight the need for strategies tailored to the specific concerns of these groups. For example, educational efforts may be particularly important for animal researchers who never preregistered studies, while senior researchers and those working in the biomedical field may benefit from discipline-specific examples demonstrating how preregistration can be integrated into discovery-driven, exploratory research. Addressing key concerns, such as the bureaucratic burden and time constraints faced by animal researchers, may further require structural support, such as user-friendly preregistration templates, integration of the authorization procedure with preregistration, clear institutional guidelines, and administrative support for preregistration. Finally, implementing incentives (e.g., publication and funding benefits, recognition in research assessments) that recognize preregistration and transparent reporting may help foster engagement. Taken together, these measures would represent essential steps toward the successful implementation of preregistration in Switzerland and possibly elsewhere.

## Supporting information

S1 File Codebook of all survey items used in the study and differences from those used by Spitzer and Mueller (2023). (XLSX)

S2 File Psychometric properties of the analyzed scales and subscales. (DOCX)

S3 File Comparison of preregistered and non-preregistered analyses. (DOCX)

S1 Fig Attention check item. (DOCX)

S1 Table Deviations from the preregistered analysis plan. (DOCX)

S2 Table Results on general experiences with study preregistration. (DOCX)

S3 Table Subsample comparison of demographic characteristics between participants with and without preregistration experience. (DOCX)

S4 Table Statistics on the multivariate and univariate effects reported for each predictor separately. (DOCX)

S5 Table Overview of qualitative codes – Barriers. (DOCX)

S6 Table Overview of qualitative codes – Facilitators. (DOCX)

S7 Table Overview of qualitative codes – Suggestions. (DOCX)

## Abbreviations

ACD: Advisory Committee to the Director
ANCOVAs: analyses of covariance
AWO: Animal Welfare Officers
CIs: confidence intervals
FSVO: Federal Food Safety and Veterinary Office
MANCOVA: multivariate analysis of covariance
PCA: principal component analyses
TOST: two one-sided tests
ωH: omega hierarchical
