# Supplementary material for: A survey of researchers’ attitudes to preregistration in animal research reveals multiple perceived barriers to adoption
Source: PLoS Biol. 2026 Jul 28;24(7):e3003511. doi: 10.1371/journal.pbio.3003511 (PMC13411886; doi:10.1371/journal.pbio.3003511)
Supplement: S2 File — (DOCX) [file pbio.3003511.s002.docx]

**S2 File: Psychometric quality**

All psychosocial construct scales were assumed to be unidimensional, but this assumption has not been examined in prior research. We therefore tried to establish strict or essential unidimensionality of the attitudes, perceived social norms, perceived behavioral control, intentions, motivations, and obstacles towards preregistration scales. While strict unidimensionality refers to the situation where there truly exists only one underlying dimension for a scale or construct, essential unidimensionality refers to the situation where there is a dominant general factor or component but nevertheless there is some sort of multidimensionality in terms of subdimensions present. It is important to establish whether the general factor is strong enough to allow for using an overall composite scale score for the construct.

Several rules and indices have been suggested to assess essential unidimensionality. Slocum-Gori and Zumbo (46) recommend a combination of parallel analysis and a criterion based on the ratio of the first to the second eigenvalue: if this ratio is greater than three or (in a stricter version) greater than four - thus, when the first eigenvalue is at least three times as high as the second eigenvalue, essential unidimensionality can be assumed even when parallel analysis suggests the existence of more than one component.

In addition, following McDonald (47) and Revelle and Zinbarg (48), we computed omega hierarchical (ωH) from a bifactor model to assess essential unidimensionality. A bifactor structure means that there is a general factor indicating unidimensionality, but also two or more so-called group factors that are modelled simultaneously. While Cronbach’s alpha and also the “normal” coefficient ω represent the proportion of shared variance in the data vis-à-vis to total variance, ωH represents the proportion of variance in overall scores that can be attributed to a single general factor, while variation in scores coming from the existence of group factors is treated as measurement error (49). Values of ωH above .70 are generally taken as evidence that a scale is sufficiently unidimensional for practical purposes.

For scales that showed clear (strict) unidimensionality (we assumed this when a PCA-based parallel analysis suggested only one component) we used coefficient ω (and not α) as a measure of reliability. The reason is that alpha’s assumption of an essential τ-equivalent model (implying equal factor loadings for all items) is seldom warranted and can lead to an underestimation of reliability when loadings are not equal for all items (congeneric model). On the other hand, ω is suitable for this case since it is computed directly from the factor loadings of a unidimensional solution.

Prior to conducting the analyses described above we checked the appropriateness of a PCA for the items referring to the respective scales using Bartlett’s test of sphericity and the Kaiser-Meyer-Olkin (KMO) measure of sampling adequacy. While Bartlett’s test checks whether there are any non-zero-correlations at all among the items under study (a condition that is almost never violated in applied settings), the KMO describes the suitability of a set of items for factor or principal component analysis in terms of the degree the correlations among variables can be explained by one or more underlying components.

Results showed that the Bartlett’s test of sphericity was highly significant for all scales analyzed in the following (all *p*s < .001), thus the minimum precondition of correlation matrices differing from an identity matrix was given for all subsequent PCAs. With regard to the KMO, the overall measure of sampling adequacy was .96 for the Attitudes Scale, .82 for the Subjective Norms Scale, .57 for the Perceived Behavioral Control Scale, .73 for the Intentions Scale, .95 for the Motivations Scale, and 0.81 for the Obstacles Scale. Thus, the overall KMO was quite high (> .8) for four of the six constructs studied here, and it was in the acceptable range (> .5) for all constructs.

**Attitudes Scale**

A parallel analysis based on principal components analysis (from now on abbreviated as PA-PCA) using the mean eigenvalues of 1,000 randomly generated samples indicated a two-component structure, accounting for 58.5% of the total variance (Component 1 = 51.8%, Component 2 = 6.7%) of attitudes towards preregistration. The eigenvalues were 11.92 and 1.54 for the first and second component, respectively, with a ratio of the first to the second eigenvalue of 7.72. The eigenvalue of the second component was only slightly above chance level according to the PA-PCA. Coefficient ωH was .95. These two criteria strongly suggest essential unidimensionality of the Attitudes Scale.

A one-component PCA was computed (see Table A) where most items loaded strongly (>.70) on the single component. However, Items 9 and 21 showed low factor loadings (.24 and .14) as well as low corrected item-total correlations (.22 and .13). In addition, upon closer examination, both items did not align well conceptually with the construct of attitudes and were therefore excluded to obtain a highly homogenous Attitudes Scale. The final revised 21-item Attitudes Scale showed excellent reliability (ω = .96) and was used in all subsequent analyses reported in the main manuscript.

**Table A: Psychometric properties and component loadings of the Attitude Scale (23 items)**

| **Items** | ***N*** | ***M*** | ***SD*** | **r.drop^a^** | **Component Loading** |
| --- | --- | --- | --- | --- | --- |
| 1. Preregistration is important to me. | 370 | -1.34 | 1.61 | 0.79 | 0.83 |
| 2. I have more trust in research findings when the study has been preregistered. | 369 | -1.18 | 1.68 | 0.81 | 0.84 |
| 3. I have more trust in researchers who preregister their studies than in those who do not. | 371 | -1.26 | 1.67 | 0.81 | 0.84 |
| 4. My field of science benefits from preregistration. | 370 | -1.12 | 1.67 | 0.81 | 0.84 |
| 5. Preregistration should be mandatory for all types of studies. | 370 | -1.80 | 1.45 | 0.67 | 0.71 |
| 6. Preregistration should be mandatory only for specific types of studies (e.g. confirmatory studies). | 368 | -0.25 | 1.85 | 0.47 | 0.51 |
| 7. Preregistration hinders exploratory/discovery research. (R)^b^ | 367 | -1.32 | 1.62 | 0.51 | 0.55 |
| 8. Preregistration should be an evaluation criterion in selection decisions (e.g. publication, research evaluation, funding etc.). | 368 | -1.50 | 1.61 | 0.76 | 0.79 |
| 9. Preregistration can be exploited (e.g. by cheating). (R)^b^ | 365 | -0.70 | 1.37 | 0.22 | 0.24 |
| 10. Preregistration does not improve research substantially. (R)^b^ | 368 | -1.02 | 1.60 | 0.80 | 0.82 |
| 11. Preregistration cannot prevent questionable research practices. (R)^b^ | 364 | -1.42 | 1.46 | 0.55 | 0.59 |
| 12. Preregistration can prevent selective reporting (i.e., only reporting significant results or results compatible with the hypotheses). | 364 | -0.03 | 1.78 | 0.56 | 0.59 |
| 13. Preregistration can prevent p-hacking (i.e., misusing data analyses to find patterns that can be presented as statistically significant). | 360 | -0.37 | 1.64 | 0.64 | 0.66 |
| 14. Preregistration can prevent HARKing (i.e., adjusting hypotheses to the observed results). | 360 | -0.03 | 1.74 | 0.69 | 0.71 |
| 15. Preregistration can prevent publication bias (i.e., only publishing studies with positive/significant results). | 360 | -0.49 | 1.83 | 0.70 | 0.73 |
| 16. Preregistration improves a study’s quality. | 360 | -0.78 | 1.68 | 0.82 | 0.85 |
| 17. Preregistration increases the credibility of animal research. | 358 | -0.72 | 1.76 | 0.81 | 0.83 |
| 18. The costs of preregistering a study are higher than the benefits. (R)^b^ | 355 | -1.09 | 1.55 | 0.63 | 0.67 |
| 19. Preregistering studies is generally unnecessary. (R)^b^ | 355 | -0.71 | 1.70 | 0.76 | 0.79 |
| 20. Preregistration makes science more transparent. | 356 | -0.02 | 1.79 | 0.79 | 0.82 |
| 21. There is a large variety of tools available to create preregistrations (for example templates and repositories). | 329 | 0.03 | 0.72 | 0.13 | 0.14 |
| 22. Preregistration is not useful in practice. (R)^b^ | 350 | -0.92 | 1.54 | 0.79 | 0.82 |
| 23. A preregistration badge (i.e., a public acknowledgment that a study was preregistered provided by many journals) increases my trust in a study. | 352 | -0.81 | 1.68 | 0.79 | 0.81 |

*Note. N* = number of participants who answered the item; *M* = mean; *SD* = standard deviation.

^a^Corrected item-total correlation, indicating how well each item correlates with the total scale score when that item is excluded.

^b^Reverse-coded item.

**Subjective Norms Scale**

The PA-PCA indicated a one-component structure, accounting for 48.9% of the total variance of subjective norms regarding preregistration. The ratio of the first to the second eigenvalue was 3.15 (with eigenvalues of 3.42 and 1.09 for the first two components). Coefficient ωH was .80. Both criteria supported unidimensionality of the Subjective Norms Scale.

A one-component PCA was computed (Table B). All but two items loaded strongly (>.70) on the component: Item 6 had a loading of .60., while Item 2 showed a very low component loading of .13 and a low corrected item-total correlation (.10). Moreover, Item 2 did not conceptually align with the construct of subjective norms and was therefore excluded to obtain a more homogenous scale. The final revised 6-item Subjective Norms Scale showed a good reliability of ω = .84 and was further used in the analyses reported in the main paper.

**Table B: Psychometric properties and component loadings of the Subjective Norms Scale (7 items)**

| **Items** | ***N*** | ***M*** | ***SD*** | **r.drop^a^** | **Component Loading** |
| --- | --- | --- | --- | --- | --- |
| 1. My peers or colleagues motivate me to preregister my studies. | 346 | -1.58 | 1.40 | 0.67 | 0.79 |
| 2. I want to be part of the Open Science Movement. | 346 | 0.94 | 1.54 | 0.10 | 0.13 |
| 3. My supervisor/superior wants me to preregister my studies. | 331 | -1.14 | 1.36 | 0.60 | 0.73 |
| 4. My co-authors want me to preregister my studies. | 341 | -1.33 | 1.36 | 0.71 | 0.84 |
| 5. Preregistration is highly acknowledged in my research community. | 346 | -1.66 | 1.35 | 0.69 | 0.83 |
| 6. I feel social pressure to preregister my studies. | 349 | -1.47 | 1.47 | 0.45 | 0.60 |
| 7. I think that most researchers in my field preregister their studies. | 349 | -2.15 | 1.10 | 0.52 | 0.70 |

*Note. N* = number of participants who answered the item; *M* = mean; *SD* = standard deviation.

^a^Corrected item-total correlation, indicating how well each item correlates with the total scale score when that item is excluded.

**Perceived Behavioral Control Scale**

The PA-PCA suggested a three-component solution. The three components accounted for 74.2% of the total variance, with eigenvalues of 2.28, 1.73, and 1.18 for Components 1 through 3, respectively. The ratio of the first to the second eigenvalue was 1.32, and the coefficient ωH was .46. Thus, unidimensionality could not be assumed for the Perceived Behavioral Control Scale.

Since the third eigenvalue (1.18) was only slightly above chance level in the PA-PCA, and because the loading pattern of the three-component oblimin-rotated solution was inconclusive with two items showing substantial cross-loadings, a two-component solution was considered. This solution explained 57.4% of the overall variance and showed a clear simple structure (Table C). The first component, with high loadings of items 1, 4, 5, and 6, represented the availability of resources and support from colleagues and supervisors for preregistration and was labeled Perceived Behavioral Control – Resources Subscale. The second component, with high loadings of items 2, 3, and 7, represented the knowledge needed for preregistration (two items) and the authority to decide about preregistration (one item), and was named Perceived Behavioral Control – Knowledge Subscale. While Perceived Behavioral Control – Resources Subscale (4 items) showed acceptable reliability with ω = .72, the reliability estimate of the Perceived Behavioral Control – Knowledge Subscale (3 items) was questionable with ω = .65 but that we accepted for our exploratory study. Thus, both subscales were ultimately used in the analyses presented in the main manuscript.

**Table C: Psychometric properties and component loadings of the Perceived Behavioral Control Scale (7 items)**

| **Items** | ***N*** | ***M*** | ***SD*** | **Component Loading 1** | **Component Loading 2** |
| --- | --- | --- | --- | --- | --- |
| 1. It is or it would be easy for me to preregister my studies. | 348 | -1.3 | 1.4 | 0.68 | -0.18 |
| 2. I know how to create and upload a preregistration. | 346 | -1.3 | 1.6 | 0.17 | 0.71 |
| 3. It is my decision to preregister my studies or not. | 351 | 0.88 | 1.8 | -0.12 | 0.65 |
| 4. I do not have the time nor the resources to preregister my studies. (R)^a^ | 347 | -1.19 | 1.5 | 0.70 | -0.19 |
| 5. My supervisor/superior does not support preregistration. (R)^a^ | 330 | -0.12 | 1.4 | 0.78 | 0.25 |
| 6. My co-authors do not support preregistration. (R)^a^ | 336 | -0.22 | 1.3 | 0.82 | 0.02 |
| 7. I do not feel well informed about preregistration. (R)^a^ | 347 | -0.84 | 1.7 | -0.05 | 0.83 |

*Note. N* = number of participants who answered the item; *M* = mean; *SD* = standard deviation.

^a^Reverse-coded item.

**Intentions Scale**

The PA-PCA of the 3-item Intentions Scale indicated a single underlying component. The ratio of the first to the second eigenvalue was 7.20 (with eigenvalues of 2.46 and 0.34 for the first two components). The single component explained 82% of the total variance with high factor loadings for all three items. Coefficient ωH was .89. These values support the appropriateness of the one-component solution, and all three items were used in the calculation of the scale score. The reliability estimate was ω = .89.

**Table D: Psychometric properties and component loadings of the Intention Scale (3 items)**

| **Items** | ***N*** | ***M*** | ***SD*** | **r.drop^a^** | **Component Loading** |
| --- | --- | --- | --- | --- | --- |
| 1. I will preregister my studies in the future. | 346 | -1.16 | 1.57 | 0.81 | 0.92 |
| 2. I am open to the idea of preregistering my research in the future. | 348 | -0.43 | 1.96 | 0.81 | 0.92 |
| 3. I do not intend to preregister my future study/studies. (R)^b^ | 347 | -0.69 | 1.91 | 0.73 | 0.87 |

*Note. N* = number of participants who answered the item; *M* = mean; *SD* = standard deviation.

^a^Corrected item-total correlation, indicating how well each item correlates with the total scale score when that item is excluded.

^b^Reverse-coded item.

**Motivations Scale**

For the 10-items Motivations Scale, a single-component structure was suggested by the PA-PCA. The ratio of the first to the second eigenvalue was 8.48 (with eigenvalues of 6.54 and 0.77 for the first two components). The single component thus represented 65.4% of the total variance. Factor loadings were high for most items (only two items loaded below .80, see Table E), supporting the unidimensionality of the Motivations Scale.

The unidimensionality was confirmed by a high omega hierarchical (ωH = .94) and the reliability estimated by coefficient omega was also excellent (ω = .94).

**Table E: Psychometric properties and component loadings of the Motivations Scale (10 items)**

| **Items** | ***N*** | ***M*** | ***SD*** | **r.drop^a^** | **Component Loading** |
| --- | --- | --- | --- | --- | --- |
| 1. I feel like preregistration is an investment in my future (e.g., it is helpful for my credibility or career). | 347 | -1.29 | 1.56 | 0.81 | 0.86 |
| 2. I believe that it is becoming harder to publish studies that were not preregistered. | 346 | -1.24 | 1.50 | 0.50 | 0.57 |
| 3. Preregistration helps plan studies better. | 348 | -0.65 | 1.84 | 0.77 | 0.82 |
| 4. The preregistration badge (i.e., a public acknowledgment that a study was preregistered provided by many journals) would be an incentive for me to preregister my studies. | 348 | -0.76 | 1.68 | 0.76 | 0.81 |
| 5. I feel morally compelled to preregister my studies. | 347 | -1.36 | 1.51 | 0.76 | 0.81 |
| 6. Preregistration makes studies more transparent. | 348 | -0.46 | 1.86 | 0.81 | 0.85 |
| 7. Preregistration makes studies more trustworthy. | 347 | -0.93 | 1.72 | 0.87 | 0.90 |
| 8. I want others to be able to comment on my planned studies. | 347 | -0.73 | 1.72 | 0.62 | 0.68 |
| 9. Preregistration helps researchers protect themselves from their own biases. | 346 | -0.45 | 1.71 | 0.82 | 0.86 |
| 10. Preregistration represents good scientific practice. | 347 | -0.38 | 1.68 | 0.82 | 0.87 |

*Note. N* = number of participants who answered the item; *M* = mean; *SD* = standard deviation.

^a^Corrected item-total correlation, indicating how well each item correlates with the total scale score when that item is excluded.

**Obstacles Scale**

A PA-PCA on the 10-item Obstacle Scale revealed a two-component structure (eigenvalues of 3.79 and 1.60), explaining 53.9% of the total variance. Although the ratio of first to the second eigenvalue (3.79/1.60 = 2.37) was below the cutoff of 3 for essential unidimensionality, this assumption was supported by omega hierarchical (ωH = .73).

Since the evidence was not clearly in favor of essential unidimensionality, we adopted the two-component solution presented in Table F. Items 3 to 6 and 9 and 10 had high loadings on the first component. This dimension reflected practical obstacles to preregistration, particularly disadvantages in terms of time and effort, and was labeled Practical Obstacles Subscale. The second component with substantial to high loadings of items 1, 2, 7 and 8 represented concerns about scooping, confidentiality, and competitive disadvantages, and was labeled Competitive Obstacles Subscale.

The Practical Obstacles Subscale (6 items) showed a good reliability (ω = .84), whereas the Competitive Obstacles Subscale (4 items) demonstrated a reliability that was questionable (ω = .66) but that we deemed acceptable for our exploratory purposes. For the analyses reported in the main manuscript, we used both newly developed subscales.

**Table F: Psychometric properties and component loadings of the Obstacle Scale (10 items)**

| **Items** | **N** | ***M*** | ***SD*** | **Component Loading 1** | **Component Loading 2** |
| --- | --- | --- | --- | --- | --- |
| 1. Preregistration puts me at a disadvantage in comparison to those who do not preregister. | 346 | 0.29 | 1.6 | 0.23 | 0.52 |
| 2. I am concerned that after pre-registering my studies others will find errors in, or deviations from, my study plans. | 346 | -0.37 | 1.5 | -0.11 | 0.43 |
| 3. I do not like that preregistration limits my flexibility in research. | 346 | 1.43 | 1.5 | 0.62 | 0.20 |
| 4. Preregistration incurs a considerable time cost. | 345 | 1.82 | 1.1 | 0.84 | -0.06 |
| 5. Preregistration is a bureaucratic exercise. | 347 | 1.67 | 1.4 | 0.81 | 0.08 |
| 6. For me, there are not enough incentives to preregister my studies. | 346 | 1.33 | 1.4 | 0.50 | 0.10 |
| 7. I would be afraid of scooping (i.e., someone taking my idea and publishing it before me) when preregistering my study. | 348 | 1.20 | 1.6 | -0.01 | 0.85 |
| 8. I would be unsure about confidentiality issues and intellectual property rights when preregistering. | 347 | 1.50 | 1.5 | -0.01 | 0.83 |
| 9. For my projects, preregistration is unnecessary. | 345 | 1.35 | 1.6 | 0.81 | -0.12 |
| 10. Preregistration would slow down the scientific progress of my project. | 348 | 1.32 | 1.5 | 0.82 | 0.02 |

*Note. N* = number of participants who answered the item; *M* = mean; *SD* = standard deviation.
