# Supplementary material for: A survey of researchers’ attitudes to preregistration in animal research reveals multiple perceived barriers to adoption
Source: PLoS Biol. 2026 Jul 28;24(7):e3003511. doi: 10.1371/journal.pbio.3003511 (PMC13411886; doi:10.1371/journal.pbio.3003511)
Supplement: S3 File — (DOCX) [file pbio.3003511.s003.docx]

**S3 File: Preregistered vs. non-preregistered analyses**

**Preregistered simple linear regressions**

In line with the preregistered analysis plan, we first examined bivariate associations between each predictor and each outcome using simple linear regression models (one predictor at a time). These analyses capture the total (unadjusted) associations between predictors and outcomes, without controlling for other variables. The reported *p*-values were not corrected for multiple testing and thus have to be viewed with caution. We refrained from corrections here since these analyses serve mainly descriptive purposes.

Overall, preregistration experience showed consistent positive associations with attitudes, subjective norms, perceived behavioral control (both subscales), intentions, and motivations, as well as negative associations with practical obstacles. Years of animal research experience showed small but consistent negative associations with attitudes, intentions, and motivations, and a positive association with practical obstacles. Associations involving gender, field of research, and organization of employment were generally smaller and more outcome-specific. Detailed results are presented in Table A.

**Table A: Simple linear regressions**

| **Attitudes Scale** | | | | |
| --- | --- | --- | --- | --- |
| **Predictor** | ***b*** | **SE** | ***p*** | ***R*²** |
| Preregistration experience | 1.24 | 0.26 | < .001 | .060 |
| Years of animal research | −0.03 | 0.01 | < .001 | .043 |
| Gender (Female) | 0.19 | 0.13 | .147 | .006 |
| Field (Basic biological research) | −0.41 | 0.19 | .031 | .026 |
| Field (General biology) | 0.36 | 0.19 | .057 |  |
| Organization (Private industry) | 0.44 | 0.23 | .060 | .023 |
| Organization (Other) | 0.48 | 0.20 | .015 |  |
| **Subjective Norms Scale** | | | | |
| **Predictor** | ***b*** | **SE** | ***p*** | ***R*²** |
| Preregistration experience | 1.08 | 0.22 | < .001 | .062 |
| Years of animal research | −0.00 | 0.01 | .610 | .001 |
| Gender (Female) | 0.02 | 0.11 | .828 | .000 |
| Field (Basic biological research) | −0.01 | 0.16 | .926 | .000 |
| Field (General biology) | 0.06 | 0.16 | .729 |  |
| Organization (Private industry) | 0.04 | 0.20 | .849 | .001 |
| Organization (Other) | 0.07 | 0.17 | .675 |  |
| **Perceived Behavioral Control – Resources Subscale** | | | | |
| **Predictor** | ***b*** | **SE** | ***p*** | ***R*²** |
| Preregistration experience | 0.90 | 0.24 | < .001 | .039 |
| Years of animal research | −0.01 | 0.01 | .099 | .008 |
| Gender (Female) | 0.01 | 0.12 | .917 | .000 |
| Field (Basic biological research) | 0.29 | 0.17 | .088 | .028 |
| Field (General biology) | 0.49 | 0.17 | .004 |  |
| Organization (Private industry) | 0.08 | 0.21 | .715 | .000 |
| Organization (Other) | −0.01 | 0.18 | .934 |  |
| **Perceived Behavioral Control – Knowledge Subscale** | | | | |
| **Predictor** | ***b*** | **SE** | ***p*** | ***R*²** |
| Preregistration experience | 1.31 | 0.29 | < .001 | .055 |
| Years of animal research | 0.01 | 0.01 | .083 | .009 |
| Gender (Female) | −0.52 | 0.14 | < .001 | .040 |
| Field (Basic biological research) | 0.31 | 0.21 | .138 | .008 |
| Field (General biology) | 0.22 | 0.21 | .280 |  |
| Organization (Private industry) | −0.55 | 0.25 | .028 | .060 |
| Organization (Other) | −0.93 | 0.21 | < .001 |  |
| **Intentions Scale** | | | | |
| **Predictor** | ***b*** | **SE** | ***p*** | ***R*²** |
| Preregistration experience | 1.37 | 0.37 | < .001 | .037 |
| Years of animal research | −0.04 | 0.01 | < .001 | .035 |
| Gender (Female) | 0.41 | 0.18 | .024 | .015 |
| Field (Basic biological research) | −0.42 | 0.26 | .111 | .021 |
| Field (General biology) | 0.51 | 0.26 | .052 |  |
| Organization (Private industry) | 0.38 | 0.33 | .244 | .008 |
| Organization (Other) | 0.34 | 0.28 | .219 |  |
| **Motivations Scale** | | | | |
| **Predictor** | ***b*** | **SE** | ***p*** | ***R*²** |
| Preregistration experience | 1.15 | 0.31 | < .001 | .039 |
| Years of animal research | −0.03 | 0.01 | < .001 | .046 |
| Gender (Female) | 0.28 | 0.15 | .059 | .011 |
| Field (Basic biological research) | −0.60 | 0.21 | .005 | .049 |
| Field (General biology) | 0.58 | 0.21 | .007 |  |
| Organization (Private industry) | 0.55 | 0.27 | .041 | .013 |
| Organization (Other) | 0.14 | 0.23 | .540 |  |
| **Practical Obstacles Subscales** | | | | |
| **Predictor** | ***b*** | **SE** | ***p*** | ***R*²** |
| Preregistration experience | −0.82 | 0.24 | < .001 | .034 |
| Years of animal research | 0.01 | 0.01 | .022 | .015 |
| Gender (Female) | −0.19 | 0.11 | .093 | .009 |
| Field (Basic biological research) | 0.29 | 0.17 | .085 | .019 |
| Field (General biology) | −0.26 | 0.17 | .112 |  |
| Organization (Private industry) | −0.18 | 0.21 | .384 | .004 |
| Organization (Other) | −0.17 | 0.18 | .337 |  |
| **Competitive Obstacles Subscales** | | | | |
| **Predictor** | ***b*** | **SE** | ***p*** | ***R*²** |
| Preregistration experience | −0.38 | 0.24 | .118 | .007 |
| Years of animal research | −0.01 | 0.01 | .406 | .002 |
| Gender (Female) | 0.23 | 0.12 | .050 | .012 |
| Field (Basic biological research) | 0.15 | 0.17 | .364 | .032 |
| Field (General biology) | −0.51 | 0.17 | .002 |  |
| Organization (Private industry) | −0.06 | 0.21 | .762 | .001 |
| Organization (Other) | −0.10 | 0.18 | .568 |  |

*Note.* *b* = unstandardized regression coefficient; SE = standard error; *R*² = proportion of variance explained. Reference categories: No preregistration experience, Male, Basic and experimental medical research, Academia.

**Preregistered multiple linear regressions**

We next conducted multiple linear regression analyses including all predictors simultaneously to examine their independent (partial) associations with each outcome. As for the simple linear regressions above, the reported *p*-values were not corrected for multiple testing. Overall, the pattern of findings from the preregistered multiple linear regression analyses closely mirrored the results of the non-preregistered multivariate and univariate ANCOVA models. Across both analytic approaches, *preregistration experience* emerged as the most consistent and robust correlate of outcomes, showing positive associations with the attitudes scale, subjective norms scale, perceived behavioral control – resources subscale, perceived behavioral control – knowledge subscale, intentions scale, and motivations scale, as well as negative associations with the practical obstacles subscale, while no consistent association was observed for the competitive obstacles subscale. In both the preregistered regression framework and the non-preregistered MANCOVA/ANCOVA framework, preregistration experience accounted for the largest proportion of explained variance across outcomes, indicating that it was the strongest predictor among those examined.

Similarly, *years of animal research* experience showed small but consistent negative associations with the attitudes scale, intentions scale, and motivations scale across both analyses, whereas associations with subjective norms, perceived behavioral control, and obstacles were weak or inconsistent and did not remain robust after correction in the ANCOVA models. These converging results indicate that greater research experience was primarily related to less favorable attitudes and lower intentions and motivations to preregister, rather than to perceived norms, control, or obstacles.

Associations involving *gender*, *field of research*, and *organization of employment* were comparatively smaller and more outcome-specific in both analytic approaches. Gender differences were largely confined to the perceived behavioral control – knowledge subscale, with women reporting lower self-perceived knowledge, a pattern that was evident in both the preregistered regression models and the non-preregistered ANCOVA. Differences by field of research were most pronounced for the attitudes scale, motivations scale, and competitive obstacles subscale, particularly contrasting researchers in General Biology with other fields, while organization of employment was primarily associated with perceived knowledge about preregistration, with lower scores observed among participants working outside academia. Across outcomes, these predictors contributed modest proportions of explained variance, consistent with the small effect sizes observed in the non-preregistered ANCOVA analyses.

Taken together, the convergence of results across the preregistered regression-based analyses and the non-preregistered MANCOVA/ANCOVA strengthens confidence in the robustness of the observed associations and suggests that preregistration experience represents the most salient correlate of attitudes, perceived control, intentions, motivations, and perceived obstacles related to preregistration, whereas other demographic and professional characteristics show more limited and outcome-specific associations. Detailed results are reported below.

**Preregistered multiple linear regressions**

Across the eight multiple linear regression models—using the attitudes scale, subjective norms scale, perceived behavioral control – resources subscale, perceived behavioral control – knowledge subscale, intentions scale, motivations scale, practical obstacles subscale, and competitive obstacles subscale as dependent variables—model explanatory power was generally modest (*R*² ≈ .05–.15). Sample sizes ranged from 329 to 351 due to listwise deletion of missing data. Preregistration experience emerged as the most consistent correlate across outcomes, whereas gender, field, organization, and years of animal research experience showed more outcome-specific associations.

- **Attitudes scale**

The model predicting the attitudes scale was statistically significant (*N* = 351; *R*² = .122, adjusted *R*² = .104; *F*(7,343) = 6.79, *p* < .001). More years of animal research experience were associated with less positive attitudes (*b* = −0.025, *p* < .001; *β* = −0.18), while preregistration experience was associated with more positive attitudes (*b* = 1.070, *p* < .001; *β* = 0.88). Squared semipartial correlations (*ΔR*²) indicated that preregistration experience (*ΔR*² = .048) and years of animal research experience (*ΔR*² = .038) accounted for the largest unique shares of explained variance.

- **Subjective Norms Scale**

The model for the subjective norms scale was significant (*N* = 335; *R*² = .057, adjusted *R*² = .036; *F*(7,327) = 2.81, *p* = .008). Preregistration experience was the only predictor showing a clear association, with higher subjective norms among participants with preregistration experience (*b* = 1.048, *p* < .001; *β* = 1.05). The *ΔR²* decomposition showed that preregistration experience accounted for nearly all explained variance in the model (*ΔR²* = .055), with negligible contributions from the remaining predictors.

- **Perceived Behavioral Control – Resources Subscale**

The model predicting the perceived behavioral control – resources subscale was statistically significant (*N* = 333; *R*² = .063, adjusted *R*² = .043; F(7,325) = 3.11, *p* = .003). Preregistration experience was positively associated with perceived behavioral control (*b* = 0.829, *p* = .001; *β* = 0.79). Field of research was also associated with this outcome, with higher scores in the General Biology field compared with the reference field (*b* = 0.369, p = .033; *β* = 0.35). *ΔR*² values indicated that preregistration experience (*ΔR*² = .033) and field (*ΔR*² = .020, grouped) contributed most to explained variance.

- **Perceived behavioral control – Knowledge Subscale**

The perceived behavioral control – knowledge subscale showed the strongest overall model fit (*N* = 333; *R*² = .154, adjusted *R*² = .136; *F*(7,325) = 8.46, *p* < .001). Preregistration experience was positively associated with perceived knowledge-related control (*b* = 1.423, *p* < .001; *β* = 1.11). Gender was also associated with this outcome, with lower scores among women compared with the reference category (*b* = −0.458, *p* < .001; *β* = −0.36). Employment in private industry was negatively associated with perceived control relative to the reference organization (*b* = −0.869, *p* < .001; *β* = −0.68). *ΔR*² indicated that preregistration experience (*ΔR*² = .057), organization (*ΔR*² = .054), and gender (*ΔR*² = .034) made the largest unique contributions.

- **Intentions Scale**

The model for the intentions scale was significant (*N* = 331; *R*² = .086, adjusted *R*² = .066; *F*(7,323) = 4.35, *p* < .001). Years of animal research experience were negatively associated with intentions (*b* = −0.031, *p* = .003; *β* = −0.16), whereas preregistration experience was positively associated with intentions (*b* = 1.226, *p* = .002; *β* = 0.75). Both predictors showed comparable unique contributions to explained variance (years: *ΔR*² = .032; preregistration experience: *ΔR*² = .032).

- **Motivations Scale**

The model predicting the motivations scale was statistically significant (*N* = 330; *R*² = .122, adjusted *R*² = .102; *F*(7,322) = 6.36, *p* < .001). Greater animal research experience was associated with lower motivation scores (*b* = −0.028, *p* < .001; *β* = −0.18). Field of research was also associated with motivation, with lower scores in Basic Biological Research (*b* = −0.470, *p* = .033; *β* = −0.35) and higher scores in General Biology (*b* = 0.434, *p* = .047; *β* = 0.32) compared with the reference field. Preregistration experience was positively associated with motivation (*b* = 1.065, *p* < .001; *β* = 0.80). ΔR² values indicated similar contributions from years of experience (*ΔR*² = .039), preregistration experience (*ΔR*² = .036), and field (*ΔR*² = .035).

- **Practical Obstacles Subscale**

The model for the practical obstacles subscale was significant (*N* = 329; *R*² = .059, adjusted *R*² = .039; *F*(7,321) = 2.88, *p* = .006). Preregistration experience was associated with fewer perceived practical obstacles (*b* = −0.791, *p* = .001; *β* = −0.77). Years of animal research experience showed a weak, but non-significant positive association with practical obstacles (*p* = .066; *β* = 0.10). Preregistration experience accounted for the largest unique share of explained variance (*ΔR*² = .033).

- **Competitive Obstacles Subscale**

Finally, the model predicting the competitive obstacles subscale was significant (*N* = 329; *R*² = .052, adjusted *R*² = .031; *F*(7,321) = 2.49, *p* = .017). Field of research was the primary correlate, with lower competitive obstacles reported in General Biology compared with the reference field (*b* = −0.536, *p* = .003; *β* = −0.51). Gender showed a borderline association (*p* = .051; *β* = 0.22), whereas preregistration experience was not significantly associated with this outcome (*p* = .127). *ΔR*² values indicated that field explained the largest unique proportion of variance (*ΔR*² = .029).
