# Supplementary figures and images for: A survey of researchers’ attitudes to preregistration in animal research reveals multiple perceived barriers to adoption
Source: PLoS Biol. 2026 Jul 28;24(7):e3003511. doi: 10.1371/journal.pbio.3003511 (PMC13411886; doi:10.1371/journal.pbio.3003511)

**S1 Fig: Attention check item**

**
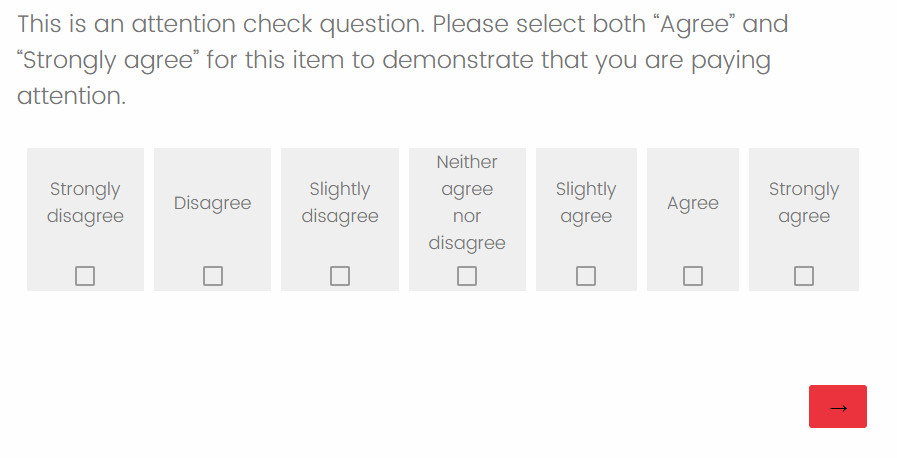
**

Supplement: S1 Fig — (DOCX) [file pbio.3003511.s004.docx]
