# Supplementary material for: A survey of researchers’ attitudes to preregistration in animal research reveals multiple perceived barriers to adoption
Source: PLoS Biol. 2026 Jul 28;24(7):e3003511. doi: 10.1371/journal.pbio.3003511 (PMC13411886; doi:10.1371/journal.pbio.3003511)
Supplement: S1 Table — (DOCX) [file pbio.3003511.s005.docx]

**S1 Table: Deviations from preregistration plan**

| **Deviations** | | | | | |
| --- | --- | --- | --- | --- | --- |
|  | **Details** | | **Original Wording** | **Deviation Description** | **Reader Impact** |
| 1 | Type | Analysis | Participants who will not respond correctly to the attention check will be excluded from the analysis (*This is an attention check question. Please select both “Agree” and “Strongly agree” for this item to demonstrate that you are paying attention.).* Additionally, participants who complete the survey more than once using the same entry code will be excluded from the analysis, except for their first completed attempt. | Participants with more than 90% missing data were also removed from the analysis. | The deviation has no impact on the study findings, as the incomplete responses were unusable for statistical analysis and would not have contributed meaningfully to the results. |
|  | Reason | New knowledge |  |  |  |
|  | Timing | After data access |  |  |  |
| 2 | Type | Variables | The items used in the survey are adapted from the questionnaire developed by Spitzer and Mueller, who report high reliabilities for all six scales: attitudes towards study preregistration, subjective norms, perceived behavioral control, intentions, motivations, and obstacles with regard to study preregistration. However, it is not clear whether these scales are really unidimensional. Therefore, we will conduct Principal Component Analyses (PCA) to explore the underlying structure of the items in the survey within each of the six outcomes. To arrive at an appropriate number of components we will use the scree criterion together with the parallel analysis criterion (using the eigenvalue means of 1000 random samples for comparison). We generally expect the scales to be unidimensional, but in case that there is more than one component according to the above criteria, the extraction will be followed by an oblique Oblimin-rotation to arrive at a simple structure even in case of correlated components. | The PCA analysis revealed that two of the six original scales exhibited a two-component structure. Consequently, each was divided into two subscales:   - Perceived Behavioral Control Scale:   - Resources Subscale   - Knowledge Subscale - Obstacles Scale   - Practical Obstacles Subscale   - Competitive Obstacles Subscale | The use of the two new subscales improved the psychometric quality of the instrument and allowed for a closer conceptual alignment with the tested constructs. This refinement increased the precision of the questionnaire and led to a more robust analysis. |
|  | Reason | New knowledge |  |  |  |
|  | Timing | After data access |  |  |  |
| 3 | Type | Analysis | Also, we will report the internal consistency Cronbach’s Alpha for each scale and set the cut-off for satisfactory alpha values at 0.7 or above. | Cronbach’s α was replaced by McDonald’s ω as the primary measure of internal consistency. In addition, subscales with reliability estimates slightly below the preregistered cut-off of .70 were retained.  The reason for using McDonald’s ω instead of Cronbach’s α was that α’s assumption of an essential τ-equivalent model (implying equal factor loadings for all items) is seldom warranted and can lead to an underestimation of reliability when loadings are not equal for all items (congeneric model). It became clear to us only after finishing the preregistration that ω would be the preferred measure for our data and justified its use in our detailed analyses on Psychometric quality (S3) but failed to report in the preregistration deviation table (now included). Although we preregistered a cut-off of .70, this was done assuming unidimensional scales. However, the PCA combined with Parallel Analysis presented in S3 indicated that several scales were multidimensional. To preserve construct validity, we split these scales into theoretically meaningful subdimensions. Two of the resulting subscales showed a slightly lower reliability than .70 (ω = .65 for the Perceived Behavioral Control – Knowledge Subscale and ω = .66 for the Competitive Obstacles Subscale). We accepted this for our exploratory purposes and also in light of the low number of items of these subscales (3 and 4, respectively) that often come with lower reliabilities. | This deviation has no impact on the interpretation of the results. The use of McDonald’s ω provides a more accurate estimate of internal consistency, and retaining slightly lower-reliability subscales preserves theoretically meaningful constructs without affecting the overall conclusions. |
|  | Reason | New knowledge |  |  |  |
|  | Timing | After data access |  |  |  |
| 4 | Type | Analysis | For the association analysis, we will conduct simple and multiple linear regression models using the scale scores for attitudes, subjective norms, perceived behavioral control, intentions, motivations, and obstacles as dependent variables. To explore bivariate associations between the predictors and the dependent variables, we will run simple regressions (one predictor at a time) for each of the six dependent variables separately. The predictors included in the univariable linear regression models will be:   - Gender (“male” vs. “female”) - Research experience (“animal research experience” in years as continuous variable) - Preregistration experience (“prior experience with preregistration” vs. “no experience with preregistration”) - Field of animal research (“basic biology research” vs. “general biology” vs. “basic and experimental research”) - Type of institution (“academia vs. non-academia”).   In a subsequent step, we will conduct six separate multiple linear regression models (one for each dependent variable) and we will fit all five predictors in the regression models independent of their significance in the univariable analysis. This is done to explore the unique associations of each predictor with the dependent variables. Unstandardized and standardized partial regression coefficients as well as the squared semipartial correlations ($\Delta R^{2})$will be reported. | Simple and multiple linear regression models with the Preregistration Scale scores as dependent variables were initially considered for the association analysis and preregistered on OSF. However, because the study included multiple conceptually related outcome variables, the analytical approach was adapted. Rather than estimating separate regression models for each outcome, a multivariate analysis of covariance (MANCOVA) was conducted to allow for the simultaneous modeling of these correlated dependent variables, while controlling for covariates. Significant multivariate effects were subsequently followed up with (multiple testing corrected) univariate analyses of covariance (ANCOVAs) and appropriate post hoc comparisons to examine effects on individual outcome variables.  The original preregistered *multiple* linear regression models are still part of our analyses: the univariate ANCOVA models following up the significant MANCOVA effects are statistically equivalent to multiple regressions.  The original preregistered *simple* linear regression analyses were conducted and are presented in the Supplementary Materials (S9 Preregistered vs. non-preregistered analyses). These results are consistent with the main MANCOVA findings. | Using MANCOVA instead of separate regression models allowed correlated preregistration outcomes to be analyzed jointly, improving statistical efficiency and reducing the risk of inflated Type I error. The approach provides clear omnibus tests for predictors, followed by focused outcome-specific analyses, resulting in more robust and interpretable inferences for the reader. |
|  | Reason | New knowledge |  |  |  |
|  | Timing | After data access |  |  |  |
| 5 | Type | Analysis | Differences between the study sample and the population of all study directors with active licenses for animal experimentation in Switzerland will be calculated for the variables below. Point estimates and confidence intervals will be reported for all variables.   - Age: two-sided t-test. - Gender (“male” vs. “female” in each of the two samples): chi-squared test. - Years since the registration as study directors: two-sided t-test. | To assess comparability between participants and non-participants an equivalence testing approach was used instead. This deviation was made in response to a reviewer suggestion, highlighting that interpreting non-significant *p*-values as evidence of similarity is inappropriate (Table 1 fallacy). Consistent with current methodological recommendations, the new approach is more suitable for assessing potential selection bias and representativeness in observational studies. | This change does not affect the interpretation of the study results. Instead, it improves transparency and avoids potentially misleading conclusions, providing readers with a clearer and more appropriate description of sample characteristics and limitations regarding generalizability. |
|  | Reason | New knowledge |  |  |  |
|  | Timing | After data access |  |  |  |
| **Unregistered Steps** | | | | | |
|  | **Details** | | **Original Wording** | **Unregistered Step Description** | **Reader Impact** |
| 1 | Type | Analysis | - | An unplanned comparison of sociodemographic characteristics was conducted between participants who had preregistered and those who never preregistered a study, due to the large sample size difference between the groups. Two-sample *t*-tests were used for mean differences and Fisher's Exact Test for differences in categorical variables. | This unplanned analysis just gives more context about the sociodemographic differences between the two groups and has no impact on the readers interpretation of the study results |
|  | Timing | After data access |  |  |  |
