# Supplementary material for: A survey of researchers’ attitudes to preregistration in animal research reveals multiple perceived barriers to adoption
Source: PLoS Biol. 2026 Jul 28;24(7):e3003511. doi: 10.1371/journal.pbio.3003511 (PMC13411886; doi:10.1371/journal.pbio.3003511)
Supplement: S2 Table — (DOCX) [file pbio.3003511.s006.docx]

**S2 Table: General experiences with study preregistration**

| **ALL PARTICIPANTS (*N* = 418)** | **% (*n*)** | |
| --- | --- | --- |
| ***Have you ever preregistered a study?*** | | |
| Yes | 10.0% (42) | |
| No | 90.0% (376) | |
| *N* | 418 (0 missing) | |
| ***Where did you learn about preregistration?*** | | |
| Never heard before this survey | 39.2% (147) | |
| Read about it | 31.5% (118) | |
| Further education | 27.2% (102) | |
| Conversation with colleagues | 19.7% (74) | |
| Presentation at conference(s) | 12.0% (45) | |
| During my studies | 5.9% (22) | |
| Information event at workplace | 5.3% (20) | |
| Do not remember | 5.3% (20) | |
| Other | 2.1% (8) | |
| Supervisor/superior | 1.6% (6) | |
| Co-author(s) | 1.1% (4) | |
| *N* | 374 (44 missing) | |
| ***Which persons or institutions have an influence on your decision to preregister or not to preregister your study?*** | | |
| Institutional policies | 47.6% (175) | |
| Funding guidelines | 35.6% (131) | |
| Supervisor/superior | 32.6% (120) | |
| Nobody | 28.0% (103) | |
| Journal guidelines | 26.4% (97) | |
| Co-author(s) | 19.0% (70) | |
| Colleagues | 15.8% (58) | |
| Guidelines of learned societies | 9.0% (33) | |
| Other | 1.6% (6) | |
| *N* | 360 (58 missing) | |
| **ONLY IF PARTICIPANTS PREREGISTERED BEFORE (*n*** **= 42)** | | **% (*n*)** |
| ***Approximately how many studies have you preregistered before?*** | | |
| *M (SD)* | 7.6 (13.62) | |
| *Mdn* | 3.0 | |
| Range | 1 - 80 | |
| *n* | 38 (4 missing) | |
| ***On average, how many hours would it take you to preregister a study?*** | | |
| *M (SD)* | 22.9 (32.66) | |
| *Mdn* | 11.0 | |
| Range | 1 - 150 | |
| *n* | 38 (4 missing) | |
| ***What was/were the reason/s for your first preregistration? Please select every option that applies.*** | | |
| Mandatory for a project | 45.5 % (15) | |
| Self-motivated | 27.3 % (9) | |
| Suggestion from supervisor/superior | 21.2 % (7) | |
| Recommendation by co-authors | 18.2 % (6) | |
| Requirement to get funding | 15.2 % (5) | |
| Conversation with colleagues | 6.1 % (2) | |
| Other | 3.0 % (1) | |
| *n* | 33 (9 missing) | |
| ***When I preregister, I use the following repository (i.e., uploading platform) for sharing my preregistration. Please select every option that applies.*** | | |
| animalstudyregistry.org (ASR) | 29.6% (8) | |
| Offline (e.g., I only share it with my co-authors or store it at my institution) | 22.2% (6) | |
| ClinicalTrials.gov | 18.5% (5) | |
| Institutional platform | 14.8% (4) | |
| osf.io (Open Science Framework) | 11.1% (3) | |
| Other | 7.4% (2) | |
| AsPredicted.org | 7.4% (2) | |
| PROSPERO | 3.7% (1) | |
| Registered reports with a specific journal | 3.7% (1) | |
| preclinicaltrials.eu | 0.0% (0) | |
| researchregistry.com | 0.0% (0) | |
| Personal website | 0.0% (0) | |
| *n* | 27 (15 missing) | |
| ***What is your preferred preregistration template(s) (i.e., form that lists important elements to preregister and can be used by researchers to create their own preregistration)?*** | | |
| No preference | 52.0% (13) | |
| animalstudyregistry.org (ASR) | 20.0% (5) | |
| Other | 8.0% (2) | |
| ClinicalTrials.gov | 8.0% (2) | |
| PREPARE checklist | 8.0% (2) | |
| Do not use templates | 4.0% (1) | |
| osf.io (Open Science Framework) | 4.0% (1) | |
| preclinicaltrials.eu | 4.0% (1) | |
| AsPredicted.org | 0.0% (0) | |
| PROSPERO | 0.0% (0) | |
| researchregistry.com | 0.0% (0) | |
| *n* | 25 (17 missing) | |
| ***Why do you prefer the selected template(s)?*** | | |
| Comprehensive | 50.0% (6) | |
| Fits well with my research area | 50.0% (6) | |
| Easy to use | 41.7% (5) | |
| Other | 25.0% (3) | |
| Time-efficient | 25.0% (3) | |
| Preferred by colleagues | 16.7% (2) | |
| The only template I know | 16.7% (2) | |
| First template I used | 16.7% (2) | |
| Recommended by co-author(s) | 8.3% (1) | |
| Recommended by supervisor/superior | 0.0% (0) | |
| *n* | 12 (30 missing) | |

*Note.* *M* = mean; *SD* = standard deviation; *Mdn* = median; *N* = total sample size; *n* = subgroup sample size.
