# Supplementary material for: A survey of researchers’ attitudes to preregistration in animal research reveals multiple perceived barriers to adoption
Source: PLoS Biol. 2026 Jul 28;24(7):e3003511. doi: 10.1371/journal.pbio.3003511 (PMC13411886; doi:10.1371/journal.pbio.3003511)
Supplement: S3 Table — (DOCX) [file pbio.3003511.s007.docx]

**S3 Table: Subsample comparison – Preregistration experience vs. no preregistration experience**

| **Characteristics** | **Preregistration experience**  **(*n* = 42)** | **No preregistration experience**  **(*n* = 376)** | **Test statistic^a^**  **p-value** |
| --- | --- | --- | --- |
| **Age^b^** |  |  |  |
| *M (SD)* | 45.9 (10.49) | 47.0 (9.05) | *t*(46.99) = 0.69  .496 |
| *Mdn* | 42.0 | 46.0 |  |
| Range | 31 - 74 | 26 - 65 |  |
| *n* | 41 (1 missing) | 362 (14 missing) |  |
| **Gender** |  |  |  |
| Female | 42.9% (18) | 40.8% (152) | Fisher Exact  .758 |
| Male | 52.4% (22) | 55.8% (208) |  |
| Other / Prefer not to say | 4.8% (2) | 3.5% (13) |  |
| *n* | 42 (0 missing) | 373 (3 missing) |  |
| **Years registered as study director^b^** |  |  |  |
| *M (SD)* | 10.2 (8.34) | 10.1 (7.90) | *t*(48.47) = -0.08  .938 |
| *Mdn* | 7.0 | 8.0 |  |
| Range | 0 - 30 | 0 - 42 |  |
| *n* | 41 (1 missing) | 363 (13 missing) |  |
| **Years in animal research** |  |  |  |
| *M (SD)* | 19.4 (8.79) | 20.2 (8.85) | *t*(50.84) = 0.52  .605 |
| *Mdn* | 17.5 | 19.0 |  |
| Range | 7 - 36 | 2 - 44 |  |
| *n* | 42 (0 missing) | 372 (4 missing) |  |
| **Academic age** |  |  |  |
| *M (SD)* | 15.7 (9.95) | 17.5 (9.24) | *t*(49.46) = 1.06  .292 |
| *Mdn* | 12.0 | 16.5 |  |
| Range | 1 - 37 | 0 - 40 |  |
| *n* | 42 (0 missing) | 366 (10 missing) |  |
| **Educational attainment** |  |  |  |
| Master’s degree | 2.4% (1) | 5.6% (21) | Fisher Exact  .645 |
| PhD / Dr. med. | 52.4% (22) | 51.5% (192) |  |
| Habilitation / professorship | 42.9% (18) | 41.8% (156) |  |
| Other | 2.4% (1) | 1.1% (4) |  |
| *n* | 42 (0 missing) | 373 (3 missing) |  |
| **Seniority level** |  |  |  |
| PhD student / technician | 0.0% (0) | 4.3% (16) | Fisher Exact  .282 |
| Postdoc. / senior researcher | 33.3% (14) | 25.9% (96) |  |
| Lecturer | 2.4% (1) | 2.7% (10) |  |
| Group leader / Professor | 52.4% (22) | 60.9% (226) |  |
| Other | 11.9% (5) | 6.2% (23) |  |
| *n* | 42 (0 missing) | 372 (5 missing) |  |
| **Field of animal research** |  |  |  |
| Basic biological research^c^ | 4.8% (2) | 14.4% (54) | Fisher Exact  .140 |
| General biology^d^ | 19.0% (8) | 13.4% (50) |  |
| Basic and experimental medical research^e^ | 76.2% (32) | 72.2% (270) |  |
| *n* | 42 (0 missing) | 374 (2 missing) |  |
| **Organization of employment** |  |  |  |
| Academic | 69.0% (29) | 78.6% (294) | Fisher Exact  .119 |
| Academic & other | 0.0% (0) | 2.4% (9) |  |
| Private | 23.8% (10) | 12.6% (47) |  |
| Governmental | 7.1% (3) | 2.1% (8) |  |
| Non-profit | 0.0% (0) | 4.3% (16) |  |
| *n* | 42 (0 missing) | 374 (2 missing) |  |

*Note.* *M* = mean; *SD* = standard deviation; *Mdn* = median; *n* = subgroup sample size.

^a^ Independent *t*-tests were used for mean differences in continuous variables; Fisher Exact tests were used for percentage differences in categorical variables.

^b^ In contrast to Table 1 in the main manuscript, the values displayed here are based on our own data collection and not on the data provided by the Swiss Federal Food Safety and Veterinary Office (FSVO).

^c^ Including following fields: Biomedical Engineering; Cancer Research; Cardiovascular Research; Endocrinology; Immunology; Medical Microbiology; Neuroscience; Nutrition and Metabolism; Pathology and/or Pathophysiology; Pharmacology; Physiology; Regenerative Medicine; Toxicology; Veterinary Medicine; Virology.

^d^ Including following fields: Animal Breeding; Animal Nutrition; Animal Welfare; Ecology; Ethology; Evolution; Laboratory Animal Science; Wildlife Biology; Zoology.

^e^ Including following fields: Biochemistry; Biophysics; Cell Biology; Cytology; Developmental Biology; Embryology; Epigenetics; Experimental Microbiology; Genetics; Molecular Biology; Radiobiology; Structural Biology.

Significant results are marked with *p <0.05, **p <0.01, ***p <0.001.
