# Supplementary material for: A survey of researchers’ attitudes to preregistration in animal research reveals multiple perceived barriers to adoption
Source: PLoS Biol. 2026 Jul 28;24(7):e3003511. doi: 10.1371/journal.pbio.3003511 (PMC13411886; doi:10.1371/journal.pbio.3003511)
Supplement: S4 Table — (DOCX) [file pbio.3003511.s008.docx]

**S4 Table: Multivariate and Univariate Effects by Predictor**

| **Preregistration experience** | | | | | | | | |
| --- | --- | --- | --- | --- | --- | --- | --- | --- |
| **Multivariate effect** | **Value** | | | ***F*(8, 314)** | ***p*** | |  |  |
| Wilks’ Λ | 0.833 | | | 7.86 | < .001 | |  |  |
| Pillai’s Trace | 0.167 | | | 7.86 | < .001 | |  |  |
| **Univariate effect** | ***b*^a^** | | | ***F*(1, 321)** | ***p*** | | ***p* (Holm)** | $\boldsymbol{\eta}_{\boldsymbol{p}}^{\boldsymbol{2}}$ |
| Attitudes Scale | 1.124 | | | 15.70 | < .001 | | < .001 | 0.05 |
| Subjective Norms Scale | 1.038 | | | 18.67 | < .001 | | < .001 | 0.06 |
| Perceived Behavioral Control – Resources Subscale | 0.821 | | | 11.02 | .001 | | .004 | 0.03 |
| Perceived Behavioral Control – Knowledge Subscale | 1.435 | | | 24.61 | < .001 | | < .001 | 0.07 |
| Intentions Scale | 1.217 | | | 9.91 | .002 | | .004 | 0.03 |
| Motivations Scale | 1.052 | | | 11.59 | < .001 | | .004 | 0.04 |
| Obstacles – Practical Subscale | -0.791 | | | 10.32 | .001 | | .004 | 0.03 |
| Obstacles – Competitive Subscale | -0.388 | | | 2.34 | .127 | | .127 | 0.01 |
| **Animal research experience (years)** | | | | | | | | |
| **Multivariate effect** | **Value** | | | ***F*(8, 314)** | ***p*** | |  |  |
| Wilks’ Λ | 0.933 | | | 2.84 | .005 | |  |  |
| Pillai’s Trace | 0.057 | | | 2.37 | .017 | |  |  |
| **Univariate effect** | ***b*** | | | ***F*(1, 321)** | ***p*** | | ***p* (Holm)** | $\boldsymbol{\eta}_{\boldsymbol{p}}^{\boldsymbol{2}}$ |
| Attitudes Scale | -0.022 | | | 13.02 | < .001 | | .002 | 0.04 |
| Subjective Norms Scale | -0.002 | | | 0.27 | .606 | | 1.000 | 0.00 |
| Perceived Behavioral Control – Resources Subscale | -0.014 | | | 5.96 | .015 | | .076 | 0.02 |
| Perceived Behavioral Control – Knowledge Subscale | 0.004 | | | 0.27 | .602 | | 1.000 | 0.00 |
| Intentions Scale | -0.031 | | | 11.88 | < .001 | | .004 | 0.04 |
| Motivations Scale | -0.028 | | | 15.57 | < .001 | | < .001 | 0.05 |
| Obstacles – Practical Subscale | 0.012 | | | 4.80 | .029 | | .116 | 0.01 |
| Obstacles – Competitive Subscale | -0.004 | | | 0.04 | .846 | | 1.000 | 0.00 |
| **Gender** | | | | | | | | |
| **Multivariate effect** | **Value** | | | ***F*(8, 314)** | ***p*** | |  |  |
| Wilks’ Λ | 0.927 | | | 3.10 | .002 | |  |  |
| Pillai’s Trace | 0.062 | | | 2.60 | .009 | |  |  |
| **Univariate effect** | ***b*^b^** | | | ***F*(1, 321)** | ***p*** | | ***p* (Holm)** | $\boldsymbol{\eta}_{\boldsymbol{p}}^{\boldsymbol{2}}$ |
| Attitudes Scale | 0.047 | | | 1.99 | .159 | | .477 | 0.01 |
| Subjective Norms Scale | 0.019 | | | 0.16 | .687 | | 1.000 | 0.00 |
| Perceived Behavioral Control – Resources Subscale | -0.077 | | | 0.03 | .858 | | 1.000 | 0.00 |
| Perceived Behavioral Control – Knowledge Subscale | -0.509 | | | 17.13 | < .001 | | < .001 | 0.05 |
| Intentions Scale | 0.238 | | | 4.89 | .028 | | .194 | 0.01 |
| Motivations Scale | 0.133 | | | 3.91 | .049 | | .246 | 0.01 |
| Obstacles – Practical Subscale | -0.112 | | | 2.57 | .110 | | .439 | 0.01 |
| Obstacles – Competitive Subscale | 0.233 | | | 4.21 | .041 | | .246 | 0.01 |
| **Field of animal research** | | | | | | | | |
| **Multivariate effect** | **Value** | | | ***F*(16, 628/630)** | | ***p*** |  |  |
| Wilks’ Λ | 0.890 | | | 2.36 | | .002 |  |  |
| Pillai’s Trace | 0.103 | | | 2.13 | | .006 |  |  |
| **Univariate effect** | ***b*^c^**  **(Basic biological research vs. reference)** | | ***b*^c^ (General biology vs. reference)** | ***F*(2, 321)** | | ***p*** | ***p* (Holm)** | $\boldsymbol{\eta}_{\boldsymbol{p}}^{\boldsymbol{2}}$ |
| Attitudes Scale | -0.387 | | 0.349 | 5.15 | | .006 | .044 | 0.03 |
| Subjective Norms Scale | 0.064 | 0.008 | | 0.02 | | .979 | 1.000 | 0.00 |
| Perceived Behavioral Control – Resources Subscale | 0.294 | 0.308 | | 2.65 | | .072 | .360 | 0.02 |
| Perceived Behavioral Control – Knowledge Subscale | 0.067 | 0.091 | | 0.31 | | .733 | 1.000 | 0.00 |
| Intentions Scale | -0.184 | 0.347 | | 1.51 | | .223 | .892 | 0.01 |
| Motivations Scale | -0.474 | 0.433 | | 6.40 | | .002 | .015 | 0.04 |
| Obstacles – Practical Subscale | 0.163 | -0.108 | | 1.01 | | .366 | 1.000 | 0.01 |
| Obstacles – Competitive Subscale | 0.093 | -0.536 | | 4.95 | | .008 | .046 | 0.03 |
| **Organization of employment** | | | | | | | | |
| **Multivariate effect** | **Value** | | | ***F*(16, 628/630)** | | ***p*** |  |  |
| Wilks’ Λ | 0.903 | | | 2.05 | | .009 |  |  |
| Pillai’s Trace | 0.103 | | | 2.13 | | .006 |  |  |
| **Univariate effect** | ***b*^d^**  **(Private vs. reference)** | | ***b*^d^**  **(Other vs. reference)** | ***F*(2, 321)** | | ***p*** | ***p* (Holm)** | $\boldsymbol{\eta}_{\boldsymbol{p}}^{\boldsymbol{2}}$ |
| Attitudes Scale | 0.326 | | 0.307 | 2.29 | | .103 | .720 | 0.01 |
| Subjective Norms Scale | 0.046 | 0.042 | | 0.17 | | .847 | 1.000 | 0.00 |
| Perceived Behavioral Control – Resources Subscale | -0.023 | 0.001 | | 0.01 | | .988 | 1.000 | 0.00 |
| Perceived Behavioral Control – Knowledge Subscale | -0.894 | -0.411 | | 9.10 | | < .001 | .001 | 0.05 |
| Intentions Scale | 0.185 | 0.131 | | 0.43 | | .654 | 1.000 | 0.00 |
| Motivations Scale | 0.039 | 0.268 | | 0.48 | | .617 | 1.000 | 0.00 |
| Obstacles – Practical Subscale | -0.070 | -0.075 | | 0.22 | | .805 | 1.000 | 0.00 |
| Obstacles – Competitive Subscale | -0.138 | 0.063 | | 0.48 | | .621 | 1.000 | 0.00 |

*Note.* *F* = *F*-statistic; *η²* = partial eta squared (effect size); *SE* = standard error; *df* = degrees of freedom; *t* = *t*-statistic. *b* = unstandardized regression coefficient; *F* = F-statistic; *p* = unadjusted p-value; *p* (Holm) = Holm-adjusted p-value; $\eta_{p}^{2}$= partial eta squared.

^a^ Reference category – No preregistration experience.

^b^ Reference category – Male.

^c^ Reference category – Basic and experimental medical research.

^d^ Reference category – Academia.
