# Supplementary material for: A survey of researchers’ attitudes to preregistration in animal research reveals multiple perceived barriers to adoption
Source: PLoS Biol. 2026 Jul 28;24(7):e3003511. doi: 10.1371/journal.pbio.3003511 (PMC13411886; doi:10.1371/journal.pbio.3003511)
Supplement: S5 Table — (DOCX) [file pbio.3003511.s009.docx]

**S5 Table: Overview of qualitative codes – Barriers**

| **Used open-ended items** | **Number of responses (*n*)** |
| --- | --- |
| **All participants** |  |
| *What do you perceive as drawbacks of preregistration?* | 142 |
| *What do you think would be the long-term negative consequences of mandatory preregistration?* | 140 |
| **Only if participants never preregistered** |  |
| *What are the reasons for not preregistering your studies?* | 313 |
| **Only if participants preregistered before** |  |
| *I am now less motivated to preregister than I was before because…* | 5 |
| **Codes** | **Participants mentioning the code at least once (*n*)** |
| **Practical and structural barriers** |  |
| Practical barriers |  |
| Bureaucratic / administrative / extra work | 107 |
| Time consuming | 81 |
| Scooping | 72 |
| Causes delays | 17 |
| Redundant - e.g. Animex | 15 |
| External complaints / harassment | 10 |
| Loss of intellectual property | 10 |
| Increases (research) costs | 9 |
| Not possible due to confidentiality agreements | 6 |
| Lack of resources | 5 |
| Internal preregistration | 4 |
| Not compatible with industry | 3 |
| Not compatible with patent system | 2 |
| No connection to Animex | 2 |
| Structural barriers |  |
| Not interested / not necessary | 31 |
| Not requested by journals, funders, institutions etc. | 28 |
| Not (only) my decision | 8 |
| Nobody checks the preregistration forms / No feedback | 7 |
| Not commonly established | 6 |
| Lack of harmonization | 3 |
| No support from research partners / co-authors | 2 |
| No incentives | 1 |
| **Knowledge barriers** |  |
| Lack of knowledge | 76 |
| Unaware of the benefits and impact | 42 |
| Lack of guidance | 7 |
| **Scientific barriers** |  |
| Not suitable for exploratory/fundamental research | 74 |
| Lack of flexibility | 48 |
| Blocks innovation | 20 |
| Too many deviations | 27 |
| Loss of creativity | 14 |
| **Regulatory barriers** |  |
| Reduction in the number of studies / animal research | 22 |
| Reduces international competitivity | 18 |
| Overregulation in Switzerland | 16 |
| Relocation to other countries | 16 |

*Note.* *n* = subgroup sample size.
