# Supplementary material for: A survey of researchers’ attitudes to preregistration in animal research reveals multiple perceived barriers to adoption
Source: PLoS Biol. 2026 Jul 28;24(7):e3003511. doi: 10.1371/journal.pbio.3003511 (PMC13411886; doi:10.1371/journal.pbio.3003511)
Supplement: S6 Table — (DOCX) [file pbio.3003511.s010.docx]

**S6 Table: Overview of qualitative codes – Facilitators**

| **Used open-ended items** | **Number of responses (*n*)** |
| --- | --- |
| **All participants** |  |
| *What do you perceive as benefits of preregistration?* | 140 |
| *What do you think would be the long-term benefits of mandatory preregistration?* | 140 |
| **Only if participants preregistered before** |  |
| *I am now more motivated to preregister than I was before because…* | 6 |
| **Codes** | **Participants mentioning the code at least once (*n*)** |
| **Transparency and integrity drivers** |  |
| Transparency and openness |  |
| More transparency | 30 |
| Reduces parallel research | 10 |
| Better public / external understanding | 9 |
| Open Science support | 4 |
| Increases trustworthiness | 4 |
| Increases credibility | 3 |
| Scientific rigor and integrity |  |
| Higher research quality | 19 |
| Prevents publication bias / all data is known | 16 |
| Better study design / thorough methods | 15 |
| Improves reproducibility / reliability | 10 |
| Good research practices / prevention of QRPs | 8 |
| Better consideration of animal use (3R) | 8 |
| Uniformity of protocols / experiments | 7 |
| Better statistical analysis | 5 |
| Prevents HARKing | 5 |
| Prevents p-haking | 4 |
| Less deviations | 3 |
| Reduces researcher bias | 2 |
| **Collaboration and protection of intellectual property** |  |
| Encourages scientific feedback & exchange | 16 |
| Provides a track record | 4 |
| Facilitates collaborations & synergies | 2 |
| Protects intellectual property | 2 |
| **Practical and Contextual Incentives** |  |
| Better (study) planning | 18 |
| Helps drafting papers / Speeds up the journal review process | 2 |
| Improves time & project management | 1 |
| **No facilitators** |  |
| No benefits at all | 55 |
| Benefits for confirmatory not exploratory/fundamental research | 12 |

*Note.* *n* = subgroup sample size; QRPs = questionable research practices; 3R = replace, reduce, refine.
