# Supplementary material for: A survey of researchers’ attitudes to preregistration in animal research reveals multiple perceived barriers to adoption
Source: PLoS Biol. 2026 Jul 28;24(7):e3003511. doi: 10.1371/journal.pbio.3003511 (PMC13411886; doi:10.1371/journal.pbio.3003511)
Supplement: S7 Table — (DOCX) [file pbio.3003511.s011.docx]

**S7 Table: Overview of qualitative codes – Suggestions**

| **Used open-ended items** | **Number of responses (*n*)** |
| --- | --- |
| **All participants** |  |
| *We are interested in how we can improve various aspects of preregistration (e.g. templates, repositories, reviewing process, integrations in published articles, education etc.). Do you have any suggestions? What do you think should be improved about preregistration?* | 129 |
| *What would make you (and perhaps other researchers) preregister more often?* | 137 |
| *Do you have any suggestions as to how to lower your (or other researchers') negative perceptions of preregistration?* | 131 |
| **Codes** | **Participants mentioning the code at least once (*n*)** |
| **Practical and structural recommendations** |  |
| Easy-to-use tool / clear (or flexible) template | 34 |
| Link with Animex / Form A / grants requests | 29 |
| Flexibility / possibility to make changes | 18 |
| Strong confidentiality measures | 16 |
| Voluntary rather than mandatory | 11 |
| Offer support & resources - dedicated staff, funds etc. | 8 |
| If mandators, only for confirmatory research | 6 |
| Offer expert review of preregistered forms | 6 |
| Increase embargo | 4 |
| International implementation, not only Switzerland | 3 |
| Badges for preregistered studies | 1 |
| **Education and awareness** |  |
| Clear demonstration of benefits (for all types of research) | 34 |
| Increase knowledge / awareness | 27 |
| Information on where and how to preregister | 13 |
| Workshops / courses / continued education | 11 |
| Provide examples | 1 |
| **Incentives** |  |
| Endorsement / requirement | 5 |
| Endorsement / requirement from (good) journals | 12 |
| Endorsement / requirement from funders | 7 |
| Endorsement / requirement from scientific community | 5 |
| Endorsement / requirement from institutions | 3 |
| Endorsement / requirement from PIs | 1 |
| Incentives / benefits | 4 |
| Publishing benefits | 8 |
| Faster authorization process | 5 |
| Funding agencies benefits | 3 |
| **Close-ended items** | |
| **All participants** |  |
| *The application form for animal experiments (Animex-ch) and the preregistration template should be the same.* | |
| Disagree | 24.9% (85) |
| Neutral | 28.7% (98) |
| Agree | 46.5% (159) |
| *N* | 342 (76 missing) |
| *The application form for animal experiments (Animex-ch) and the preregistration template should be separated from each other.* | |
| Disagree | 39.8% (137) |
| Neutral | 31.1% (107) |
| Agree | 29.1% (100) |
| *N* | 344 (74 missing) |
| *Certain sections of the application form for animal experiments (Animex-ch) should be linked and uploaded to the preregistration template.* | |
| Disagree | 21.0% (72) |
| Neutral | 28.6% (98) |
| Agree | 50.4% (173) |
| *N* | 343 (75 missing) |

*Note.* *n* = subgroup sample size.
