# Supplementary material for: A survey of researchers’ attitudes to preregistration in animal research reveals multiple perceived barriers to adoption
Source: PLoS Biol. 2026 Jul 28;24(7):e3003511. doi: 10.1371/journal.pbio.3003511 (PMC13411886; doi:10.1371/journal.pbio.3003511)
Supplement: Attachment — Submitted filename: Review Wurbel.docx [file pbio.3003511.s013.docx]

**Review of the manuscript titled “Researchers‘ perspectives on preregistration in animal research” submitted to PLOS Biology**

Reviewer: Olmo van den Akker

In this manuscript, the authors surveyed study directors of animal experiments in Switzerland to elicit their experiences with and perceptions of the practice of preregistration in animal research. While the survey responses were heterogeneous, a picture emerged of study directors that have limited knowledge and experience with preregistration, which possibly influenced the skeptical attitudes about the practice.

I appreciated the high level of transparency provided in this manuscript, especially the way that the authors show how their questionnaire follows from the questionnaire used by Spitzer and Müller, and that the authors also present the available demographic characteristics of the non-participants. Below you can find a list of major and minor points for improvement.

**Major points for improvement:**

1. The DOI provided for accessing the data (<https://doi.org/10.48620/91802>) is currently inaccessible, presumably because it remains under review. Ensuring public data availability is essential for assessing validity, enabling re-analysis, and facilitating potential inclusion in systematic reviews.
2. The description of the qualitative analysis is minimal. The statement that “recurring themes were identified” does not provide enough detail to assess the analytical rigor. More information on coding procedures, number of coders, consensus strategies, and links between data excerpts and reported findings is needed.
3. The authors preregistered using Cronbach’s α with a cut-off value of 0.70, yet report McDonald’s ω instead and retain (sub)scales with ω < 0.70. These deviations are not included in the preregistration deviation table. The rationale for these changes, and their implications for reliability and validity, should be explained.
4. Multiple statistical tests are conducted without correction for multiple comparisons. The authors should either justify this decision or apply a correction. In line with this, more emphasis on effect sizes is recommended, especially where statistically significant results correspond to very small effect sizes (e.g., η² = 0.01–0.02), as it is unclear whether such effects are meaningful.
5. The authors carry out many statistical tests but do not correct for multiple testing. The authors should convincingly explain why they did not do so or implement a correction for multiple testing that ensures the findings are not merely based on randomness. What could help here is to focus more on effect sizes. These are more informative, especially since there are many cases where associations are statistically significant but effect sizes are low (eta-squared = 0.01 – 0.02). Can these really be interpreted as substantial effects?
6. Some of the obstacles perceived by the participants can be easily rebutted (e.g. that deviations are discouraged or even forbidden). I would like to see the authors do this in their discussion section as it is quite worrisome that such misconceptions about preregistration remain and seem to influence uptake and attitudes.
7. The recommendations offered in the conclusion (infrastructure, education, incentive systems) are very broad. More concrete and actionable suggestions, perhaps tailored to different stakeholders would improve the manuscript’s utility. Example: “*Providing the necessary infrastructure, appropriate educational options and a well-designed incentive system might thus help foster the use of preregistration in the animal research community*.”
8. To what extent are the results of participants who never heard of preregistration relevant? Shouldn’t they be left out given that they have no insight into the practice, or shouldn’t the sample be split into those who had at least heard of the practice and those who had not?

**Minor points for improvement:**

1. The introduction is quite comprehensive but the authors should acknowledge that there is also research highlighting that preregistration is currently not as effective as it could be. For example, there are observational studies that show that undisclosed deviations are common and that the positive effects of preregistration are not consistently found across studies.
2. A link to more information about Animex-ch would be helpful.
3. *“Additionally, one more reminder was issued at the institutional level by the Animal Welfare Officers (AWO). At the time of the study, 49 institutions in Switzerland had designated AWOs*” 🡪 Does this mean that not all directors received an additional reminder? If so, could this have introduced bias into the sample?
4. It is not entirely clear why some (sets of) questions are labeled under psychosocial constructs and others are not. It can be argued that ‘obstacles’ do not really tap into participants’ psychology, while it can equivalently be argued that the perceptions of barriers to preregistration do tap into psychology. It seems the main difference is the way they are measured so maybe the description should focus on that. The authors could be clearer about this and maybe even disregard the term “psychosocial constructs” altogether.
5. The response rate of 30.2% is way higher than what the authors (and myself) expected. Why do the authors think this is the case? Does this have any ramifications for the interpretation of the results?
6. The authors mention in the preregistration that they would first assess the appropriateness of doing a PCA, but they don’t mention the Kaiser-Meyer-Olkin (KMO) measure of sampling adequacy and Bartlett's test of sphericity in the manuscript. It would be good to state whether these ‘tests’ were indeed carried out.
7. The authors use a MANCOVA framework instead of the preregistered linear regressions (and disclose this in the preregistration deviation table). Because these regressions were planned as the most important analyses in the study, I would encourage the authors to do these regressions regardless and present the results in a supplement. I also think it would be good if the authors would discuss the influence this deviation had on statistical power.
8. Why did the authors more comprehensively present Wilks’ Lambda than Pillai’s Trace when presenting the results? A sentence in the manuscript explaining this would be useful.
9. The process for removing items with “poor psychometric properties” should be described in more detail so readers know which items were removed and why.
10. How do the authors reconcile contradictory survey results? For example, that preregistration is seen by some as more time-consuming and by others as less time-consuming, or that preregistration is seen by some as conducive to collaboration and by others as hampering it.
11. “*This study represents the first attempt to evaluate the researchers’ experience with, and perspectives on, preregistration in animal research*” 🡪 I don’t think this claim can be made given that the authors cite some references that also do this (e.g., Wieschowski et al., 2020).
12. “*However, the gender difference could also reflect that men tend to overestimate their knowledge more often than women, suggesting that women lower ratings may indicate more accurate or honest self-evaluations rather than a true lack of capacity.”* 🡪 This claim requires references
13. Speculation about the negative experiences or privacy considerations of participants who did not disclose their gender seems excessive, particularly given the small number of respondents in this category, and should be moderated.
14. The claim that the sample is representative of all animal scientists in Switzerland is not supported, since study directors constitute a specific subgroup. I think this statement should be reconsidered.
15. The limitation on page 34 is outlined well but I would like to see some more discussion of how this specific limitation could have biased the results.
