# Supplementary material for: A survey of researchers’ attitudes to preregistration in animal research reveals multiple perceived barriers to adoption
Source: PLoS Biol. 2026 Jul 28;24(7):e3003511. doi: 10.1371/journal.pbio.3003511 (PMC13411886; doi:10.1371/journal.pbio.3003511)
Supplement: Attachment — Submitted filename: Response_to_Reviewers.docx [file pbio.3003511.s014.docx]

**RESPONSE TO REVIEWRS’ COMMENTS**

We want to thank the reviewers for taking the time to carefully review our manuscript and for providing very constructive and useful feedback on the analysis, presentation, and interpretation of our results. We have addressed and replied to all of their comments below and indicated our changes to the manuscript where appropriate. We believe that the revisions have considerably strengthened the manuscript and enhanced its clarity.

| **Reviewer #1 [Ulf Toelch]** | |
| --- | --- |
| **Reviewer’s general comment** | **Authors’ response** |
| Priboi et al. present in their manuscript "Researchers' perspectives on preregistration in animal research" a valuable survey results on the perception of preregistration of animal researchers in Switzerland. The presented data is an important contribution that will inform policy not only in Switzerland and beyond. To increase the impact, the authors may consider my comments below. The study is complex including a preregistration and many supplemental tables. I apologise to the authors that I could not assess the whole material. Time given for review times at PLOS Biology are extremely short and reviewers who contribute their time for free are constantly harassed with emails about the status of their review. In the short time allowed, I was not able to do an in depth comparison of the preregistration and the actual manuscript. This already shows that for preregistrations to become useful also publisher and editors need to allow for slower but more rigorous science. | We thank reviewer #1 for their positive feedback and thoughtful comments on their review. We have addressed the reviewer’s comments, and we believe that our revisions facilitate understanding of the complex data and analyses. |
| **Reviewer’s specific comments** | **Authors’ response** |
| 1. Value of preregistration: The authors start with the slight overstatement that preregistration is impactful and at the same time acknowledge that evidence backing this claim is not conclusive yet (ll62/63). Animal researchers already preregister their study with regulatory authorities even though this may not be as exhaustive as a formal preregistration and not include all the details. It is unclear in particular in the discussion what a formal preregistration would add to the animal permits. With the negative attitudes displayed by researchers how does this connect to the thematic analysis what researchers want and how realistic are these demands? Overall, the manuscript would benefit from a less enthusiastic description of preregistration as the panacea to problems in animal research. | We appreciate this comment for raising two important points. In response, we did the following:   1. We softened the claims regarding the impact of preregistration throughout the “Introduction” and explicitly acknowledge that empirical evidence is still limited. See changes done between LL 56-67. 2. We now explain in more detail how applications for licensees to conduct animal experimentation in Switzerland and elsewhere differ from preregistration. First, these applications have a different purpose as they serve ethical evaluation and implementation of the 3Rs. And, secondly, these applications are confidential and thus they cannot provide the transparency on the research process that preregistration is aimed at. See LL 718-721 in the “Discussion” section. |
| 2. Comparison of participants and non participants: The authors compare study participants to non participants. This is done via t-tests and an absence of significance is interpreted as similarity between groups. There is good evidence that such an approach is not optimal (Table 1 fallacy). If the authors are interested in confounders or selection bias, the three comparions described here are insufficient to conclude that study populations were similar in the sense that results here will generalise to the general population of study directors. This should be acknowledged in the discussion. The authors may want to consider removing p-values in the comparison of the two groups. | We agree that our interpretation of non-significant p-values as evidence of similarity was inappropriate. In the revised manuscript, we no longer rely on null hypothesis significance testing to assess comparability. Instead, we applied an equivalence-based approach (LL 228-234). We further clarify in the limitation section that this approach does not fully rule out selection bias or establish complete comparability between groups (LL 776-778). |
| 3. Psychological scales: The scales are analysed in much detail. The reader is, however, lost in numbers. A Figure summarising some of the scale results would be beneficial to get a better grasp of magnitude of effects. Also there are multiple comparisons and only on some scales there are differences for example for gender. This leads to meaningless comparisons such as: "Follow-up pairwise comparisons (S7 Table) revealed that participants who preferred not to state their gender reported significantly less positive attitudes and lower motivations to preregister than participants identifying as female or male;" (ll409-411). This is based on a comparison of 14 participants vs the rest of the population. I doubt that based on this can claim that these differences exist. The reported comparisons should be evaluated carefully by the authors. | We agree that our presentation of this complex study and the many results was not ideal and made it difficult for readers to follow. In the revised version we present several figures that should address the reviewer’s comment:   - Figure 1 – a heatmap presenting an overview of all predictor-outcome associations - Figures 2A-E – separate figures presenting the predictor-outcome associations for each predictor   We also agree with the reservation regarding differences associated with gender. The very small number of participants who preferred not to state their gender (*n* = 14) limits the interpretation of these comparisons. We therefore recoded these responses as missing and excluded this subgroup from the analyses, restricting gender comparisons to participants identifying as female or male. This revision aligns the analyses with the preregistered analysis plan, which specified gender as a binary variable (female vs male), from which we had deviated later by including the “other/prefer not to say” category. We believe that this revision improves the robustness and interpretation of the results. |
| 4. That said, in the current form I feel it is difficult to see the overall picture. The results are presented in a way that makes it hard to grasp what the data looks like. Maybe supplementary tables contain this information but I was not able to assess these (see comment above). An additional Figure and a more selective presentation of the psychological scale results could facilitate a better understanding of the material provided. | We agree that the presentation of the results made it difficult to see the overall picture of our findings and have revised the Results section substantially. In particular, we added an overview figure (Figure 1) and predictor-specific figures (Figures 2A–E) to illustrate all of our main analyses. These figures provide a high-level view of our findings and should help readers quickly get a good overview. Furthermore, we have streamlined and simplified the “Results” section by eliminating the details of the statistical analyses from the text and instead referring to tables and figures. We believe that these changes have substantially improved the readability of the paper and will make it easier for readers to grasp both the overall picture and the relevant patterns in the data. |

| **Reviewer #2 [Andrew H. Tyner]** | |
| --- | --- |
| **Reviewer’s general comment** | **Authors’ response** |
| In "Researchers' perspectives on preregistration in animal research," the authors adapt a questionnaire from Spitzer and Mueller (2023) to explore attitudes, norms, and experiences with preregistration among a sample of Swiss study directors working in animal research. The authors present their data in rich detail through psychometric analyses of closed-ended items and a thoroughgoing exploration of themes emerging from the open-ended items. I concur with the authors on the importance of gaining a deeper understanding of researchers' experiences with preregistration and I commend them for their impressively high response rate and the care and detail of their analyses. Below I offer a set of suggestions to improve the presentation of the research and extract more meaning out of these data. | We appreciate the reviewer’s positive and thoughtful assessment of our manuscript and their recognition of the importance of the topic. We also thank the reviewer for their constructive suggestions. In response, we have addressed all comments in detail below and have revised the manuscript accordingly. We believe that these changes strengthen the manuscript and improve its clarity. |
| **Reviewer’s specific comments** | **Authors’ response** |
| **Major comments** | |
| 1. The authors rely heavily on significance testing, particularly in Aim 3, to the exclusion of substantive interpretations of their findings and descriptively meaningful results. My main takeaway from Aim 3 is that many of these variables have statistically significant associations, many others do not, and I'm not left with any clearer understanding of what these findings tell us about challenges, opportunities, or understandings of preregistration in their sample. The supplemental materials aren't much help either - for example, in the 'Field of research' analyses the authors refer readers to Table S8, but this table (like Tables S7 and S9) only shows results on the transformed scales. What am I to make of the fact that basic and experimental medical researchers have a mean of -0.47 on the attitudes scale, while basic biological researchers have a mean of -0.70 and general biologists have a mean of -0.12? I understand that the authors are making use of the scales they developed in Aim 2, but for a general audience the authors need to be translating at least some of these results into substantively meaningful terms. I would encourage the authors to make greater use of visualizations in this manuscript to highlight some of the key relationships they see emerging in their data. In some situations these visualizations might make use of the underlying items to complement the presentation of scales. | As already mentioned in response to a similar comment made by reviewer #1, we fully agree that our presentation of the results made it difficult to get a clear picture of the pattern of associations across the different outcomes. To address this, we have substantially revised the entire “Results” section as outlined in response to points 3 and 4 of reviewer #1. |
| 2. As a related concern, the MANCOVA models presented in Aim 3 need a more careful presentation and justification. To start with, the authors should remove any language referring to effects (e.g. "A MANCOVA was conducted to examine the effects of preregistration experience, animal research experience, gender, field of research, and organization of employment on the eight Preregistration Scales and Subscales" [371-373]). These are not causal models and the data they've collected do not lend themselves to a causal analysis or interpretation. What's more, the setup of the models doesn't follow an intuitive causal ordering - they've set up the preregistration scales as the outcome variables and included preregistration experience as a predictor. The picture is undoubtedly more complicated than attitudes/norms affect behaviors, but a model where the behaviors are a primary predictor for the psychometric outcomes seems even more questionable. At the very least we'd think of behavior/experience and attitudes/norms as mutually influencing each other. Again, all of this language of causal ordering and effects is misplaced given the structure of these data - the real takeaway is that many of these predictors and constructs are statistically related. But, if the authors are going to present their findings in causal terms and set up models with predictors and outcomes, it should be done more carefully. | We agree with the reviewer that the data and analyses do not support causal inference. We have therefore removed all language referring to “effects*”* and causal ordering throughout the manuscript. The Aim 3 analyses are now consistently described in terms of associations and adjusted relationships, and the text has been revised to clarify that the models are intended to summarize statistical relationships among constructs rather than to imply causal direction. We also place greater emphasis on effect size estimates as indicators of the strength of these associations, without causal interpretation. Some examples of the changes we made are detailed below. |
| 3. The presentation of the open-ended results in Aims 4 and 5 is impressively thorough. Still, I wonder whether these results could be better summarized or organized to distill key insights and takeaways for the reader. As it stands, these sections often read like the authors are just relaying all of the open-ended responses they received. The section on Knowledge Barriers stands out as an example: "Many participants stated that they were not aware that preregistration even existed. Some were unfamiliar with the procedure, describing a lack of guidance or uncertainty about the steps involved and the appropriate platforms for their studies. Others expressed general skepticism about the potential benefits and overall impact of preregistration in science" (471-474). Help the reader navigate these findings - what percentage of researchers expressed these views? Does that vary by relevant background characteristics? What do we learn from this that we didn't get from the close-ended items? And then a few lines later in the next section: "Often, participants working in exploratory or fundamental research questioned the feasibility of preregistration for their studies" (477-478). What does often mean? What does questioning the feasibility mean? And "However, while some participants recognized these advantages, many saw no benefits at all, or found it appropriate only for confirmatory studies" (493-495) - why such imprecision in reporting? Who holds these beliefs and in what proportions? | We appreciate the recognition of the depth of the qualitative results. We analyzed the answers to the open-ended questions using thematic analysis following the approach described by Braun and Clarke (1, 2). In line with their recommendations, we identified patterns across the dataset but did not report numbers of responses on specific themes described by participants.  In the revised manuscript we now report the number of participants who mentioned each code at least once (e.g. LL 493-503), providing an indication of how frequently certain topics were raised. We also extended the information in S8 Tables 1, 2, and 3. Percentages were not calculated because the open-ended items differed in number and wording across participants (see S8 Table 1), and because participants often responded to only a subset of items, making percentage estimates difficult to interpret and not directly comparable across themes.  Subset analyses addressing research questions such as “whether reported outcomes varied by relevant background characteristics” could also not be meaningfully conducted based on the open-ended responses. The majority of responses were brief and focused on single aspects, resulting in limited thematic depth within subgroups and insufficient data to support reliable or interpretable comparisons across participant characteristics. In addition, participants sometimes addressed multiple themes within a single response, complicating attribution of themes to specific background characteristics. Addressing such research questions would require a richer qualitative dataset (e.g., in-depth interviews) with more extensive and systematically comparable responses than those collected in the present study.  We also corrected the unclear formulation regarding the feasibility of preregistration in exploratory/fundamental research (see LL 511-515). |
| 4. Finally, I was not able to access this manuscript's data since the provided DOI did not resolve. The authors mention that the files are currently under review by the team managing University of Bern's repository, so perhaps that's the reason. All the same, reviewers should have an opportunity to reproduce the authors' analyses as a matter of course. | Since the revision of the paper, the link to the data has been activated by the University of Bern BORIS repository and can be accessed here: <https://doi.org/10.48620/91802>. The repository offers access to the raw data in Excel format, the RStudio scripts used for data cleaning and analysis, and a description of the study and data.  However, it is important to note that 38 out of the 418 participants did not consent to the publication of their data in an open repository. Consequently, their responses were not included in the dataset deposited in BORIS. Also, the published RStudio script used for data cleaning was adapted accordingly, such that no information related to these 38 participant IDs is included.  At the reviewers’ request, we can provide access to the full set of 418 responses. |
| **Minor comments** | |
| 5. The authors explain their revised analytical approach in item 3 of Table S1. They write that they switched from simple and multiple linear regressions to a MANCOVA approach because of multi-category predictors. I agree with the authors that MANCOVA is an advisable approach here but not for the reasons the authors offer. The advantage of MANOVA/MANCOVA (and multivariate analyses more generally) is the simultaneous estimation of models with distinct but correlated outcome variables. It doesn't have anything to do with multi-category predictors. You can fit multiple linear regressions with multi-category predictions without a problem - like binary predictors, one level becomes the reference and the results of the other categories are interpreted in light of that reference category. | We appreciate this clarification and agree with the reviewer’s assessment. Our original explanation incorrectly attributed the choice of MANCOVA to the presence of multi-category predictors. We have revised Table 1S to clarify that the decision to use MANCOVA was due to the presence of multiple conceptually related and correlated outcome variables, which are more appropriately analyzed jointly within a multivariate framework. |
| 6. "Preregistration is a promising tool for improving transparency and quality of research, and is arguably among the most impactful Open Science practices" (lines 56-58). I'd soften this language to, "...and arguably has the potential to be among the most impactful…" The evidence base on preregistration is not strong enough yet to justify the statement as written (as the authors point out elsewhere in the manuscript). | We agree with this comment. As already mentioned in response to a similar point made by reviewer #1, we have softened the claims regarding the impact of preregistration throughout the “Introduction” by using more cautious language and by explicitly acknowledging that empirical evidence is still limited. |
| 7. "The inability to replicate published results…have questioned…" (lines 68-69". Rephrase to, "...have led to questions…" | We have changed the text accordingly – see LL 68-70. |
| 8. "...these questionable research practices can slow down scientific progress, waist animal lives for inconclusive results…" (lines 78-79). Should be "...waste animal lives…" | Thank you for catching the misspelling. We have corrected the text accordingly – see LL 79. |
| 9. "The values for non-participants are FSVO-provided data, whereas those for participants are based on survey responses. This distinction arises because FSVO collected gender data using only two categories (female, male), while our survey allowed for four options (female, male, other, and prefer not to say)" (lines 295-297). I'd recommend that the FSVO-provided data be used for both groups in the Table 1 reporting. Otherwise the data aren't directly comparable. | Unfortunately, we cannot use FSVO-provided gender data for both groups because the FSVO did not provide gender information for participants. We received only the gender distribution for non-participants. However, given the few participants choosing “other” or “prefer not to say”, and excluding these from the analysis, we believe that the data are now comparable. In contrast, for age and years since registration as study directors, the FSVO provided summary information for both participants and non-participants, which facilitated direct comparison of those variables. |
| 10. "Taken together, these results suggest that participants and non-participants were comparable in terms of age, gender distribution, and animal research experience" (lines 318-320). I'd recommend a more constrained interpretation here to avoid conflating absence of evidence with evidence of absence. Perhaps something like: "Altogether we do not find statistically significant differences between participants and non-participants in terms of age, gender distribution, or animal research experience." | We agree that our interpretation of non-significant p-values as evidence of similarity was inappropriate. As already mentioned above in response to a similar point made by reviewer #1, we have revised the text accordingly and applied an equivalence-based approach (LL 228-234). We further clarify in the limitation section that this approach does not fully rule out selection bias or establish complete comparability between groups (LL 776-778). |
| 11. "Similarly, a further survey conducted in the Netherland…" (566). Should be "...in the Netherlands…" | Thank you for noticing the misspelling. We corrected the text – see LL 631. |
| 12. "We can only speculate that these participants may have had negative experiences with preregistration and/or may have high privacy concerns that leaves them in general reluctant to disclose information, being it research ideas or private information such as their gender" (615-618). Even as the authors acknowledge that this is speculative, I find this explanation nonsensical. The reasons participants have for not disclosing their gender identities are the same reasons they don't preregister? This is implausible and frankly needless - I see it as a consequence of the over-reliance on significance testing. The authors had no reason to expect this significant result in the first place and consequently offer a haphazard interpretation of it - this is a recipe for capitalizing on noise. | We agree that the interpretation offered in the original manuscript was speculative and not sufficiently grounded in theory. Therefore, given the very small number of participants who did not disclose their gender and following the suggestion of reviewer #1, we removed this group from the analyses entirely and restricted gender comparisons to female versus male participants. The speculative interpretation referring to non-disclosure of gender has been deleted accordingly. This revision eliminates the risk of overinterpreting unstable effects based on very small subsamples. |
| 13. "This delimitation could help reduce research waste by avoiding repetition and support the refinement of future studies, contributing to overall less animals use" (650-652). I had trouble following this sentence. Consider a rephrase for clarity. | This sentence was removed in the course of the revision. |
| 14. "Second, despite being introduced to the definition of preregistration at the beginning of the survey, some participants may not have read or fully understood the concept...This is supported by requests received after survey completion from three participants who asked to have their data deleted upon realizing that they had confused the two concepts" (708-715). Kudos to the authors for this level of transparency. I agree that it's a concern (if three people wrote in with this request there's a good chance that others were also confused), but there's not much to be done about it now. So mostly I just want to thank the authors for the transparent reporting. I think it strengthens the overall credibility of the manuscript and demonstrates exemplary reporting. | Thank you! |

| **Reviewer #3 [Olmo van den Akker]** | |
| --- | --- |
| **Reviewer’s general comment** | **Authors’ response** |
| In this manuscript, the authors surveyed study directors of animal experiments in Switzerland to elicit their experiences with and perceptions of the practice of preregistration in animal research. While the survey responses were heterogeneous, a picture emerged of study directors that have limited knowledge and experience with preregistration, which possibly influenced the skeptical attitudes about the practice.  I appreciated the high level of transparency provided in this manuscript, especially the way that the authors show how their questionnaire follows from the questionnaire used by Spitzer and Müller, and that the authors also present the available demographic characteristics of the non-participants. Below you can find a list of major and minor points for improvement. | We thank the reviewer for this thoughtful assessment of our manuscript, and we appreciate their recognition of the transparency of our approach. We addressed their constructive comments in detail below and adapted the manuscript accordingly. We believe these changes strengthen the manuscript and improve its clarity. |
| **Reviewer’s specific comments** | **Authors’ response** |
| **Major comments** | |
| 1. The DOI provided for accessing the data (<https://doi.org/10.48620/91802>) is currently inaccessible, presumably because it remains under review. Ensuring public data availability is essential for assessing validity, enabling re-analysis, and facilitating potential inclusion in systematic reviews. | As already mentioned above in response to the same comment made by reviewer #2, this has been resolved. The link to the data has been activated by the University of Bern BORIS repository and can be accessed here: <https://doi.org/10.48620/91802>.  The repository offers access to the raw data in Excel format, the RStudio scripts used for data cleaning and analysis, and a description of the study and data.  However, it is important to note that 38 out of the 418 participants did not consent to the publication of their data in an open repository. Consequently, their responses were not included in the dataset deposited in BORIS. Also, the published RStudio script used for data cleaning was adapted accordingly, such that no information related to these 38 participant IDs is included.  At the reviewers’ request, we can provide access to the full set of 418 responses. |
| 2. The description of the qualitative analysis is minimal. The statement that “recurring themes were identified” does not provide enough detail to assess the analytical rigor. More information on coding procedures, number of coders, consensus strategies, and links between data excerpts and reported findings is needed. | We agree that the initial description of the qualitative analysis lacked sufficient detail. We have revised the Methods section to clarify the analytical procedure, specifying that initial coding was conducted by the first author, followed by discussion and refinement of codes with the last author (see LL 302-303). We also describe how consensus was reached and how themes were developed (see LL 306-308). Representative data excerpts are presented in Figure 3. |
| 3. The authors preregistered using Cronbach’s α with a cut-off value of 0.70, yet report McDonald’s ω instead and retain (sub)scales with ω < 0.70. These deviations are not included in the preregistration deviation table. The rationale for these changes, and their implications for reliability and validity, should be explained. | We agree with the reviewer’s comment. The reason for using McDonald’s ω instead of Cronbach’s α was that α’s assumption of an essential τ-equivalent model (implying equal factor loadings for all items) is seldom warranted and can lead to an underestimation of reliability when loadings are not equal for all items (congeneric model). It became clear to us only after finishing the preregistration that ω would be the preferred measure for our data and justified its use in our detailed analyses on Psychometric quality (S3) but failed to report this in the preregistration deviation table (now included). Although we preregistered a cut-off of .70, this was done assuming unidimensional scales. However, the PCA combined with Parallel Analysis presented in S3 indicated that several scales were multidimensional. To preserve construct validity, we split these scales into theoretically meaningful subdimensions. Two of the resulting subscales showed a slightly lower reliability than .70 (ω = .65 for the Perceived Behavioral Control – Knowledge Subscale and ω = .66 for the Competitive Obstacles Subscale). We accepted this for our exploratory purposes and also in light of the low number of items of these subscales (3 and 4, respectively) that often come with lower reliabilities. |
| 4. Multiple statistical tests are conducted without correction for multiple comparisons. The authors should either justify this decision or apply a correction. In line with this, more emphasis on effect sizes is recommended, especially where statistically significant results correspond to very small effect sizes (e.g., η² = 0.01–0.02), as it is unclear whether such effects are meaningful. | We agree with the reviewer and have revised the analyses to explicitly address multiple testing. Specifically, *p*-values for all follow-up tests were adjusted using the Holm correction, which is now clearly stated in the Methods and Results sections and reflected in the figures.  In addition, we have placed more emphasis on effect sizes throughout the manuscript. Partial eta-squared values are now consistently reported, qualified in terms of their magnitude, and visualized in new figures (Fig. 1 and Figs. 2A-E), as already mentioned above in response to a similar point made by reviewers #1 and #2.  Furthermore, we have revised the entire “Results” section to facilitate readability and getting a clearer picture of our findings. |
| 5. The authors carry out many statistical tests but do not correct for multiple testing. The authors should convincingly explain why they did not do so or implement a correction for multiple testing that ensures the findings are not merely based on randomness. What could help here is to focus more on effect sizes. These are more informative, especially since there are many cases where associations are statistically significant but effect sizes are low (eta-squared = 0.01 – 0.02). Can these really be interpreted as substantial effects? | As this comment is redundant with the comment before, we refer to our response above. |
| 6. Some of the obstacles perceived by the participants can be easily rebutted (e.g. that deviations are discouraged or even forbidden). I would like to see the authors do this in their discussion section as it is quite worrisome that such misconceptions about preregistration remain and seem to influence uptake and attitudes. | We agree that some of the perceived barriers identified in our data reflect misconceptions about preregistration that needed explicit clarification. In response, we have revised the Discussion section to directly address and rebut these misconceptions – see LL 697-709. |
| 7. The recommendations offered in the conclusion (infrastructure, education, incentive systems) are very broad. More concrete and actionable suggestions, perhaps tailored to different stakeholders would improve the manuscript’s utility. Example: “Providing the necessary infrastructure, appropriate educational options and a well-designed incentive system might thus help foster the use of preregistration in the animal research community.” | We agree that more concrete examples can improve the conclusion of the manuscript. At the same time, we were mindful not to over-interpret the qualitative data, as responses to the open-ended questions were generally brief and did not provide sufficient detail to support highly specific or stakeholder-tailored recommendations. In response, we have revised the “Conclusion” section to include examples linked directly to the barriers, facilitators, and suggestion in our findings – see LL 825-843. |
| 8. To what extent are the results of participants who never heard of preregistration relevant? Shouldn’t they be left out given that they have no insight into the practice, or shouldn’t the sample be split into those who had at least heard of the practice and those who had not? | This is a question we also asked ourselves but came to a different conclusion. Participants who reported never having heard of preregistration were not excluded because all respondents were provided with a definition of preregistration at the beginning of the survey. Thus, their understanding of preregistration when filling out the survey may not have been substantially different from those answering that they had heard of it. In both cases, having read our definition at the beginning of the survey should have allowed them to evaluate the concept and report their attitudes, perceived norms, and perceived behavioral control. Their responses should reflect informed perceptions formed at the time of the survey, which are relevant to our aim of understanding how preregistration is perceived across the population of animal researchers, including those with no prior exposure to the practice.  Our primary analytical focus was on differences between researchers with and without actual preregistration experience, rather than prior awareness alone. Splitting the sample based on prior awareness (heard vs not heard of preregistration) would address a different research question and was not part of our preregistered analysis plan. |
| **Minor comments** | |
| 9. The introduction is quite comprehensive but the authors should acknowledge that there is also research highlighting that preregistration is currently not as effective as it could be. For example, there are observational studies that show that undisclosed deviations are common and that the positive effects of preregistration are not consistently found across studies. | We softened the claims regarding the impact of preregistration throughout the “Introduction” by using more cautious language and by explicitly acknowledging that empirical evidence is still limited. See changes done between LL 56-67. |
| 10. A link to more information about Animex-ch would be helpful. | We added the link related to animex-ch in the manuscript (see LL 131). We also explained in more detail the difference between applications for animal licenses via animex-ch, which are confidential and serve ethical evaluation and the implementation of the 3Rs, and preregistration, which is public and serves transparency and evaluation of research rigor (see LL 718-721). |
| 11. “Additionally, one more reminder was issued at the institutional level by the Animal Welfare Officers (AWO). At the time of the study, 49 institutions in Switzerland had designated AWOs” 🡪 Does this mean that not all directors received an additional reminder? If so, could this have introduced bias into the sample? | We thank the reviewer for bringing this up as our wording was confusing. In Switzerland, every institution conducting animal research is obliged by law to have an AWO. Thus, the 49 institutions comprise all institutions in Switzerland conducting animal research.  Our study was discussed within the national AWO network, and all AWOs agreed to disseminate an additional reminder at the institutional level within their respective institutions. We provided them with a standardized text for their email reminder to ensure consistent wording across institutions. We did not verify whether all 49 AWOs complied with this agreement but have no reason to doubt it. We therefore consider the risk of systematic bias introduced by the AWO-mediated reminder to be negligible – see LL151-161. |
| 12. It is not entirely clear why some (sets of) questions are labeled under psychosocial constructs and others are not. It can be argued that ‘obstacles’ do not really tap into participants’ psychology, while it can equivalently be argued that the perceptions of barriers to preregistration do tap into psychology. It seems the main difference is the way they are measured so maybe the description should focus on that. The authors could be clearer about this and maybe even disregard the term “psychosocial constructs” altogether. | We thank the reviewer for raising this point. In our study, the term “psychosocial constructs” refers specifically to the six multi-item scales derived from the Theory of Planned Behavior (attitudes, subjective norms, perceived behavioral control, and intentions), as well as closely related constructs motivation and obstacle that were included in the original study by Spitzer & Müller (2023). These measures are theoretically grounded and intended to capture underlying psychological predispositions or representations rather than single, context-specific perceptions (see LL 185-188).  Other variables in the survey (e.g., socio-demographic characteristics, research background, prior awareness of preregistration, or preregistration experience) are descriptive in nature and were therefore not conceptualized as psychosocial constructs. Similarly, barriers and facilitators, examined separately under Aim 4, consist of direct, context-specific items capturing perceived external conditions rather than latent constructs measured through theoretically motivated scales.  We therefore use the term “psychosocial constructs” to distinguish these theory-based, multi-item scales from descriptive or contextual measures. We acknowledge that this distinction was not sufficiently explicit in the initial version of the manuscript and have clarified this conceptual separation in the revised text. |
| 13. The response rate of 30.2% is way higher than what the authors (and myself) expected. Why do the authors think this is the case? Does this have any ramifications for the interpretation of the results? | We agree that the response rate of 30.2% was higher than initially anticipated, and we can only speculate about the underlying reasons. Several factors may have contributed.   - The survey was disseminated through well-established and trusted institutional channels (i.e., via animex-ch, the FSVO, AWOs), which may have increased its perceived legitimacy and encouraged participation. - The survey targeted a clearly defined and relevant population using a direct, personalized approach, which may have facilitated engagement. - Issues related to open and reproducible science have been highly salient in Switzerland and elsewhere in recent years, which may have increased interest in the topic. - It is conceivable that some respondents participated because they held critical views either in favor or against preregistration and wished to express support or concerns.   Regarding implications for interpretation, the relatively high response rate and the apparent similarity in characteristics of participants and non-participants reduced our concerns about bias. However, as rightly argued by the other reviewers that our comparison between participants and non-participants does not allow us to reject the possibility of selection bias, we took a more cautious stance in the revised manuscript to avoid overgeneralization. |
| 14. The authors mention in the preregistration that they would first assess the appropriateness of doing a PCA, but they don’t mention the Kaiser-Meyer-Olkin (KMO) measure of sampling adequacy and Bartlett's test of sphericity in the manuscript. It would be good to state whether these ‘tests’ were indeed carried out. | We thank the reviewer for pointing out this missing information. We now included these results in document S3_Psychometric quality. |
| 15. The authors use a MANCOVA framework instead of the preregistered linear regressions (and disclose this in the preregistration deviation table). Because these regressions were planned as the most important analyses in the study, I would encourage the authors to do these regressions regardless and present the results in a supplement. I also think it would be good if the authors would discuss the influence this deviation had on statistical power. | As also pointed out by reviewer #2, MANCOVA is the more appropriate framework for our analyses due to the presence of multiple conceptually related and correlated outcome variables, which are more appropriately analyzed jointly within a multivariate framework. However, as the preregistered linear regression analyses were central to the original analysis plan, we followed the reviewer’s recommendation, and now also report the preregistered simple regressions (one predictor at a time) for each of the dependent variables in the Supplementary Materials (S9 Preregistered vs. non-preregistered analyses).  Regarding the implications of this deviation for statistical power, we described in the preregistration deviation table (S1 Table) that we opted to use a MANCOVA framework in the main analyses to better reflect the multivariate structure of the outcome variables. We are aware that a multivariate approach can increase statistical power to detect an overall effect of a predictor, especially when the dependent variables are correlated and the effects of the predictor align in their directions across dependent variables. To avoid inflating Type I error in the univariate follow-up ANCOVAs we now control for multiple testing across conceptually related dependent variables using Holm correction. |
| 16. Why did the authors more comprehensively present Wilks’ Lambda than Pillai’s Trace when presenting the results? A sentence in the manuscript explaining this would be useful. | Wilks’ Lambda was primarily used because it remains the most commonly reported MANOVA statistic and facilitates comparison with prior studies. Pillai’s Trace, which is more robust to violations of MANOVA assumptions, was additionally calculated to confirm the findings. As both approaches showed similar results, the more detailed presentation of Wilks’ Lambda was used to avoid redundant reporting. In the revised manuscript, we moved the relevant statistics to Supplementary Table S7, where both Wilks’ Lambda and Pillai’s Trace are reported. |
| 17. The process for removing items with “poor psychometric properties” should be described in more detail so readers know which items were removed and why. | Detailed information on the removal of items with poor psychometric properties, including which items were removed and the specific criteria underlying these decisions, is provided in the supplementary table “S3 Psychometric quality” – see LL 61-64 and LL 80-83. |
| 18. How do the authors reconcile contradictory survey results? For example, that preregistration is seen by some as more time-consuming and by others as less time-consuming, or that preregistration is seen by some as conducive to collaboration and by others as hampering it. | We agree that some survey findings appear contradictory at first glance, such as preregistration being perceived as time-consuming versus time-saving, or as facilitating versus hindering collaboration. We do not interpret these patterns as inconsistencies in the data, but rather as reflecting genuine heterogeneity in respondents’ experiences and perspectives. While several plausible explanations could be speculated (e.g., differences in study complexity or lab practices), we intentionally refrain from drawing such conclusions in the manuscript, as they go beyond what can be directly deduced from the data. We therefore present these findings descriptively, as capturing the range of views held within the surveyed population. |
| 19. “This study represents the first attempt to evaluate the researchers’ experience with, and perspectives on, preregistration in animal research” 🡪 I don’t think this claim can be made given that the authors cite some references that also do this (e.g., Wieschowski et al., 2020). | We revised the text accordingly (see LL 617-625). |
| 20. “However, the gender difference could also reflect that men tend to overestimate their knowledge more often than women, suggesting that women lower ratings may indicate more accurate or honest self-evaluations rather than a true lack of capacity.” 🡪 This claim requires references | We added the following citation to support our claim: Pallier, G. (2003). Gender differences in the self-assessment of accuracy on cognitive tasks. *Sex roles*, *48*(5), 265-276. |
| 21. Speculation about the negative experiences or privacy considerations of participants who did not disclose their gender seems excessive, particularly given the small number of respondents in this category, and should be moderated. | We agree that speculation about negative experiences or privacy considerations would be inappropriate, particularly given the small size of this subgroup. In the revised manuscript, and following the suggestion of another reviewer, we have re-coded responses from participants who preferred not to state their gender as missing and excluded this subgroup from all analyses. Gender comparisons are now restricted to participants identifying as female or male only, in line with the preregistered analysis plan. As a result, no interpretations regarding participants who did not disclose their gender remain in the manuscript. |
| 22. The claim that the sample is representative of all animal scientists in Switzerland is not supported, since study directors constitute a specific subgroup. I think this statement should be reconsidered. | We agree that our findings may not generalize to all animal scientists as our target populations were study directors. We have revised this accordingly – see LL 132-142 and LL 774-783. Our decision to target study directors was based on the fact that they are the ones who decide whether or not to preregister a study, while other personnel involved in animal experiments (animal care staff, attending veterinarians, technicians, and students) may have different views but do not usually have the power to decide on whether or not to preregister a study. |
| 23. The limitation on page 34 is outlined well but I would like to see some more discussion of how this specific limitation could have biased the results. | We have revised the “Strengths and limitations” section to more explicitly discuss how the identified limitations may have biased our findings and the likely direction of such bias – see LL 793-820. |

**References**

1.	Braun V, Clarke V. Using thematic analysis in psychology. Qualitative research in psychology. 2006;3(2):77-101.

2.	Braun V, Clarke V. In H. Cooper, P. M. Camic, D. L. Long, A. T. Panter, D. Rindskopf, & K. J. Sher (Eds), APA handbook of research methods in psychology, Vol. 2: Research designs: Quantitative, qualitative, neuropsychological, and biological (pp. 57-71). Washington, DC: American Psychological Association; 2012.
