# Supplementary material for: A survey of researchers’ attitudes to preregistration in animal research reveals multiple perceived barriers to adoption
Source: PLoS Biol. 2026 Jul 28;24(7):e3003511. doi: 10.1371/journal.pbio.3003511 (PMC13411886; doi:10.1371/journal.pbio.3003511)
Supplement: Attachment — Submitted filename: ResponseToReviwers.docx [file pbio.3003511.s015.docx]

**RESPONS TO REVIEWERS' COMMENTS:**

**Reviewer #1:** [identifies himself as Ulf Toelch]

**The revised version of the manuscript "Researchers' perspectives on preregistration in animal research" by Probi et al. has improved substantially in clarity and is an excellent read with highly relevant analyses regarding the perception and uptake of preregistrations in animal research. Also data and associated meta data have been deposited in an accessible format in an open repository. Some minor points remain that the authors may want to address.**

**1.	In agreement with reviewer #2, the text has been updated to give a less optimistic and now more nuanced view of preregistrations. What remains, however, is the first sentence of the abstract that, in prominent position, advocates preregistrations in manner that is in my opinion not yet backed up by the evidence. Even when adding "potentially" this still is a very strong statement. This is also the case in ll56/57. I again suggest to tone this down even further. Why the nitpicking on a single sentence? The paper will hopefully be used in policy discussions. These discussion will in my opinion not benefit from the strong language used in the paper. Most evidence only comes from related fields like psychology and clinical trials. Even here the evidence is mixed (e.g. outcome switching occurs despite preregistration in clinical trials, papers deviate in an undisclosed way from preregistrations in psychology). More and better evidence is needed to showcase under what circumstances preregistrations make sense and what expected benefits for the scientific system will be. The paper is an important contribution to inform these discussions. A language that signals an openness to these discussion could be helpful. Please take this only as mild nudging and not fundamental criticism of the paper at large.**

We believe that by adding “potentially” we have toned down this statement sufficiently to avoid giving a false impression about the state of current evidence. However, we maintain that preregistration is generally considered both promising and potentially impactful because of first principles (by making the research process more transparent) as well as accumulating empirical evidence. For example, an editorial in the May 27 issue of the journal Nature stated that “Registered Reports [a specific form of preregistration including peer review of the registered protocol; explanation added by us] improve the credibility of scientific claims by rewarding big questions, sound methods and solid analyses” and that “they need to become a standard tool in research.” We would therefore like to keep the sentence as is but leave the final decision to the editor.

**2.	Ll 232 the conventional threshold for equivalence tests needs a reference. I agree that the equivalence test used here is now better suited than a conventional t-test. In my opinion there is not even a need for a test. The need for a sample that is not influenced by a selection bias is not as strong as in a clinical trial. The authors also discuss the possibility of a selection bias.**

We have now added an appropriate reference (ref. 45) – see LL 237-238.

**3.	The Figures now represent the underlying data much better. There is a small typo in the text referring to Figure 2 (grater->greater, line 437). L 434 what does modest mean here. You have in the methods indicated low medium and large effect sizes. Please refer to effect sizes in this terminology. Effect sizes were largely small and some were medium. I also do not understand the word consistency here. What would consistency of effect sizes across categories mean?**

Thanks for spotting this typo – this has been corrected.

In response to the comment regarding L434, we have revised this paragraph for clarity and replaced “moderate” by “medium” to keep consistency with the effect sizes mentioned in the methods section. By “consistent” we meant that all effects differ in the expected direction across all scales and subscales. However, we have deleted this last part of the last sentence as it is not essential for understanding these results.

**Reviewer #2:** [identifies himself as Andrew H. Tyner]

**Thanks for the opportunity to re-review "Researchers' perspectives on preregistration in animal research". It's clear from the extensive revisions and detailed point-by-point responses that the authors thoughtfully considered all of the feedback they were provided and made sensible decisions on how to address it. I'm comfortable with this manuscript proceeding to publication. Below I outline my one major reservation; a handful of minor substantive suggestions; and a few remaining line-level suggestions. The line numbers I reference are for the version with tracked changes.**

**Major reservation**

**1. The reproducibility materials are now available at the link the authors provided, which is good to see. Still, I'm not able to reproduce their analyses using these materials. Part of this has to do with file names. In the readme, the data file is called "Preregistration_Study.xlsx", in the actual materials it's called "Preregistration_Study-anonymized_data", and in the R code it's called "PreReg_Study.xlsx". Additionally, "PreReg_Study.xlsx" appears to be the input of the cleaning script, the output of the cleaning script, and the input of the analysis script. I would strongly prefer to see a 'raw' data file that serves as the input to the cleaning script and then an analytic data file that's both the output of the cleaning script and the input of the analysis script. Also, when I try to run the cleaning script by changing the name of the input file, it throws an error at line 418 because it's being asked to drop columns that don't exist.**

We agree that the previous organization and naming of the files may have caused confusion and hindered reproducibility. In response, we incorporated the suggestions of Reviewer #2 and harmonized the file names across the repository, README file, and R scripts to ensure consistency throughout the reproducibility materials. We also corrected the issue in the cleaning script identified by the reviewer at line 418, where the script attempted to remove non-existing columns. We carefully rechecked all scripts and repository materials to ensure that the analyses and figures can now be reproduced using the deposited data and code. Furthermore, we followed the suggestion of Reviewer #2 and provide a “raw data” file (PreReg_Study.xlsx) as an input for the cleaning script (PreReg_Study-cleaning.R), and a “cleaned data” file (PreReg_Study_clean.Rdata ) as an input for the analysis script (PreReg_Study-analysis.R). Thus, the reproducibility of the analyses and figures can now be checked directly by running the analysis script on the cleaned data file. As mentioned in the Cover letter, the raw data file had to be deposited under restricted access on BORIS because 38 of the 418 participants had denied consent to publish their data open access. Because the files may not yet be accessible on BORIS (they are working on our amendments) and to allow the editor and the reviewers to assess computational reproducibility of our analyses and figures without asking BORIS for access (which is subject to approval and a Data Transfer and Use Agreement (DTUA)), we herewith submit the full data sets together with all other material for your consideration.

**Minor suggestions**

**2. "In addition, we used a criterion based on the ratio of the first and second eigenvalue of the PCA, as well as omega hierarchical (ωH) from a bifactor model, to assess essential unidimensionality" (lines 281-283). What was the actual criterion? The authors allude it to without defining it.**

The detailed rationale and thresholds used to assess essential unidimensionality were described in supplementary material S3 Psychometric quality. To improve clarity, we now briefly specify the criteria directly in the main Methods section, including the eigenvalue ratio criterion (> 3) and the ωH threshold (≥ .70) with the support of relevant references (refs. 46-49), while retaining the detailed explanation in the supplementary material – see LL 266-267.

**3. "When necessary, items with poor psychometric properties (low factor loadings and/or low corrected item-total correlations) and weak conceptual alignment were removed, resulting in improved internal consistency and construct validity of each scale" (lines 424-426). Which items were removed? This is the Results section, so they should just report it directly.**

Detailed information regarding the retained and removed items for each scale was already reported in Supplement S3. To improve clarity in the main Results section, we now explicitly report that Items 9 and 21 were removed from the Attitudes Scale and Item 2 was removed from the Subjective Norms Scale due to poor psychometric performance and weak conceptual alignment – see LL 383-387.

**4. "Interestingly, our analysis also revealed differences by gender and organization of employment...may indicate more accurate or honest self-evaluations rather than a true lack of capacity" (lines 813-822). On one hand, nothing they include in this paragraph is inaccurate. On the other hand, it seems slightly misleading to highlight these single instances of statistical differences without recognizing that across *most* constructs there were not statistically significant differences for gender or employment. The absence of evidence for differences is, for me, the main takeaway here.**

We agree with reviewer #2 and have revised the introduction to this section accordingly, emphasizing the lack of significant association with any other scale or subscale. It now reads: “Although no significant associations of gender and organization of employment were observed for any other Scale or Subscale, they both differed in the researchers’ perceived ability to preregister.” – LL 684-686.

**5. "This might be due to the less competitive nature resulting in less fear of being scooped in General Biology compared the more competitive fields of basic and applied biomedical research" (lines 834-837). I am not in the relevant disciplines, so for a general audience I would suggest more context on what makes basic and applied biomedical research more competitive than General Biology.**

This is based on personal experience rather than hard evidence. We have therefore revised the sentence indicating that it is unclear whether General Biology is indeed perceived as less competitive. It now reads: “Whether this indicates that General Biology is perceived as less competitive, resulting in less fear of being scooped compared to basic and applied biomedical research, is not clear.” – LL 698-700.

**6. "Although preregistration is arguably more straightforward in the case of a confirmatory study with a clearly defined hypothesis, it is another common misunderstanding that preregistration cannot be used for exploratory research. Again, this perceived barrier might be addressed by educating animal researchers about existing flexible preregistration templates (e.g., AsPredicted, OSF) that support the preregistration of specific types of research" (lines 899-908). I think this slightly misstates the problem. The question isn't *whether* preregistration of non-confirmatory research is possible; it's *why* researchers should do so. The solution here is to foreground the value proposition of preregistration in non-confirmatory research rather than merely showing that it's possible.**

We are grateful to reviewer #2 for bringing up this important point. We have added two sentences to this section (LL 745-748) to briefly explain why preregistration of exploratory research is not only possible, but actually important, supported by two relevant references. It now reads: “Although preregistration is arguably more straightforward in the case of a confirmatory study with a clearly defined hypothesis, it is another common misunderstanding that preregistration cannot be used for exploratory research. Exploratory research generates preliminary evidence and hypotheses that stimulate further avenues of research (Kimmelman et al. 2014 PLOS Biol, 10.1371/journal.pbio.1001863). It is thus in the best interest of the scientific community that exploratory research is trustworthy and useful (Dirnagl 2020 PLOS Biol, 10.1371/journal.pbio.3000690).”

**7. The particularly high administrative burdens associated with animal research in Switzerland are alluded to a few times without being explicitly defined. For those without the contextual background, what makes the administrative requirements more stringent in Switzerland compared to peer countries? This deserves a paragraph unto itself in the Discussion since it's a factor that potentially limits the generalizability of their findings.**

We did not intend to give the impression that the administrative burden associated with animal research is exceptionally high in Switzerland, and it is indeed comparable to many other countries with similar regulations, including the EU, the UK, and the USA, although the exact procedures for obtaining licenses for animal experiments may differ. We believe that this is already reflected in our wording. Thus, we wrote: “At the same time, it is paramount to acknowledge the existing administrative burden and the regulatory pressure faced by the animal research community in Switzerland and elsewhere” (LL 726-728). And: “Particularly for countries with regulatory structures similar to those in Switzerland, such as the countries within the European Union, our findings may provide useful guidance for fostering preregistration in animal research” (LL 805-808). We consider this sufficient context and would refrain from going into further detail about national differences in the exact licensing procedures, which may not be relevant for the general reluctance towards preregistration expressed by Swiss study directors.

**Line-level suggestions**

**8. "On average, participants exhibited rather unfavorable attitudes towards preregistration..." (lines 45-46). Perhaps "reported" rather than "exhibited" here.**

We have replaced “exhibited” by “reported” – L 46.

**9. "Our findings offer guidance on promising measures to enhance acceptance of preregistration among animal researchers, including raising awareness, offering education and training, and facilitating procedures" (lines 50-53). The ending of this sentence is strange. Facilitating which procedures?**

This meant “facilitating the procedure of preregistration”, which we have now specified more clearly – L 53.

**10. "A report by the US National Institute of Health working group has recommended..." (lines 97-98). Which working group are they referring to here?**

It is “The Advisory Committee to the Director (ACD) Working Group on Enhancing Rigor, Transparency, and Translatability in Animal Research”, which was established by the NIH to improve biomedical research outcomes. The working group delivered its final strategic recommendations in 2021 (see ref. 39: Wold B, Tabak L. ACD working group on enhancing rigor, transparency, and translatability in animal research: final report. Bethesda (MD): NIH Advisory Committee to the Director. 2021). We have now added the specification of the working group in the text of the manuscript – L 90-92.

**11. "associations of several predictors on the Preregistration Scales..." (line 297). Should probably be "associations of several predictors with the Preregistration Scales...".**

Yes – this has been corrected.

**12. "However, on all scales and subscales, participants ratings ranged from..." (lines 455-456). Should be "participants' ratings" instead.**

This has been corrected.
