# Supplementary material for: A survey of researchers’ attitudes to preregistration in animal research reveals multiple perceived barriers to adoption
Source: PLoS Biol. 2026 Jul 28;24(7):e3003511. doi: 10.1371/journal.pbio.3003511 (PMC13411886; doi:10.1371/journal.pbio.3003511)
Supplement: Peer review history

# Editor report: Decision Letter 0

**DOI:** 10.1371/journal.pbio.3003511.r001

**Authors:** Roland G Roberts

10 Oct 2025

Dear Dr Würbel,

Thank you for submitting your manuscript entitled "Researchers‘ perspectives on preregistration in animal research" for consideration as a Research Article by PLOS Biology.

Your manuscript has now been evaluated by the PLOS Biology editorial staff, as well as by an academic editor with relevant expertise, and I'm writing to let you know that we would like to send your submission out for external peer review.

IMPORTANT: Please change your article type to "Meta-Research Article" when you upload your additional metadata (see next paragraph).

However, before we can send your manuscript to reviewers, we need you to complete your submission by providing the metadata that is required for full assessment. To this end, please login to Editorial Manager where you will find the paper in the 'Submissions Needing Revisions' folder on your homepage. Please click 'Revise Submission' from the Action Links and complete all additional questions in the submission questionnaire.

Once your full submission is complete, your paper will undergo a series of checks in preparation for peer review. After your manuscript has passed the checks it will be sent out for review. To provide the metadata for your submission, please Login to Editorial Manager (https://www.editorialmanager.com/pbiology) within two working days, i.e. by Oct 14 2025 11:59PM.

If your manuscript has been previously peer-reviewed at another journal, PLOS Biology is willing to work with those reviews in order to avoid re-starting the process. Submission of the previous reviews is entirely optional and our ability to use them effectively will depend on the willingness of the previous journal to confirm the content of the reports and share the reviewer identities. Please note that we reserve the right to invite additional reviewers if we consider that additional/independent reviewers are needed, although we aim to avoid this as far as possible. In our experience, working with previous reviews does save time.

If you would like us to consider previous reviewer reports, please edit your cover letter to let us know and include the name of the journal where the work was previously considered and the manuscript ID it was given. In addition, please upload a response to the reviews as a 'Prior Peer Review' file type, which should include the reports in full and a point-by-point reply detailing how you have or plan to address the reviewers' concerns.

During the process of completing your manuscript submission, you will be invited to opt-in to posting your pre-review manuscript as a bioRxiv preprint. Visit http://journals.plos.org/plosbiology/s/preprints for full details. If you consent to posting your current manuscript as a preprint, please upload a single Preprint PDF.

Feel free to email us at plosbiology@plos.org if you have any queries relating to your submission.

Kind regards,

Roli Roberts

Roland Roberts, PhD

Senior Editor

PLOS Biology

rroberts@plos.org

# Peer review: Decision Letter 1

**DOI:** 10.1371/journal.pbio.3003511.r002

**Authors:** Roland G Roberts

12 Dec 2025

Dear Dr Würbel,

Thank you for your patience while your manuscript "Researchers‘ perspectives on preregistration in animal research" was peer-reviewed at PLOS Biology. It has now been evaluated by the PLOS Biology editors, an Academic Editor with relevant expertise, and by three independent reviewers.

You'll see that reviewer #1 says that the study is “an important contribution” but he wants you to be less enthusiastic about preregistration in the paper, to flag the limitations of some of your analyses, and present the analysis of psychological scales much more clearly (including an additional Fig). Reviewer #2 is impressed by the dataset, but suggests several ways in which the analysis and presentation could be much clearer, and cautions you to avoid causal wording. Reviewer #3 liked your transparency, but raises a number of concerns, including an undeclared deviation from the preregistration, failure to correct for multiple testing, a request for more concrete recommendations, and a query about separating respondents who had not heard of registration. I see that two reviewers noted that the data DOI does not currently resolve; this must be rectified.

In light of the reviews, which you will find at the end of this email, we would like to invite you to revise the work to thoroughly address the reviewers' reports.

Given the extent of revision needed, we cannot make a decision about publication until we have seen the revised manuscript and your response to the reviewers' comments. Your revised manuscript is likely to be sent for further evaluation by all or a subset of the reviewers.

In addition to these revisions, you will need to complete some formatting changes, which you will receive in a follow up email. A member of our team will be in touch with a set of requests shortly.

We expect to receive your revised manuscript within 3 months. Please email us (plosbiology@plos.org) if you have any questions or concerns, or would like to request an extension.

At this stage, your manuscript remains formally under active consideration at our journal; please notify us by email if you do not intend to submit a revision so that we may withdraw it.

**IMPORTANT - SUBMITTING YOUR REVISION**

Your revisions should address the specific points made by each reviewer. Please submit the following files along with your revised manuscript:

1. A 'Response to Reviewers' file - this should detail your responses to the editorial requests, present a point-by-point response to all of the reviewers' comments, and indicate the changes made to the manuscript.

*NOTE: In your point-by-point response to the reviewers, please provide the full context of each review. Do not selectively quote paragraphs or sentences to reply to. The entire set of reviewer comments should be present in full and each specific point should be responded to individually, point by point.

You should also cite any additional relevant literature that has been published since the original submission and mention any additional citations in your response.

2. In addition to a clean copy of the manuscript, please also upload a 'track-changes' version of your manuscript that specifies the edits made. This should be uploaded as a "Revised Article with Changes Highlighted" file type.

*Re-submission Checklist*

When you are ready to resubmit your revised manuscript, please refer to this re-submission checklist: https://plos.io/Biology_Checklist

To submit a revised version of your manuscript, please go to https://www.editorialmanager.com/pbiology/ and log in as an Author. Click the link labelled 'Submissions Needing Revision' where you will find your submission record.

Please make sure to read the following important policies and guidelines while preparing your revision:

*Published Peer Review*

Please note while forming your response, if your article is accepted, you may have the opportunity to make the peer review history publicly available. The record will include editor decision letters (with reviews) and your responses to reviewer comments. If eligible, we will contact you to opt in or out. Please see here for more details:

https://blogs.plos.org/plos/2019/05/plos-journals-now-open-for-published-peer-review/

*PLOS Data Policy*

Please note that as a condition of publication PLOS' data policy (http://journals.plos.org/plosbiology/s/data-availability) requires that you make available all data used to draw the conclusions arrived at in your manuscript. If you have not already done so, you must include any data used in your manuscript either in appropriate repositories, within the body of the manuscript, or as supporting information (N.B. this includes any numerical values that were used to generate graphs, histograms etc.). For an example see here: http://www.plosbiology.org/article/info%3Adoi%2F10.1371%2Fjournal.pbio.1001908#s5

*Blot and Gel Data Policy*

We require the original, uncropped and minimally adjusted images supporting all blot and gel results reported in an article's figures or Supporting Information files. We will require these files before a manuscript can be accepted so please prepare them now, if you have not already uploaded them. Please carefully read our guidelines for how to prepare and upload this data: https://journals.plos.org/plosbiology/s/figures#loc-blot-and-gel-reporting-requirements

*Protocols deposition*

To enhance the reproducibility of your results, we recommend that if applicable you deposit your laboratory protocols in protocols.io, where a protocol can be assigned its own identifier (DOI) such that it can be cited independently in the future. Additionally, PLOS ONE offers an option for publishing peer-reviewed Lab Protocol articles, which describe protocols hosted on protocols.io. Read more information on sharing protocols at https://plos.org/protocols?utm_medium=editorial-email&utm_source=authorletters&utm_campaign=protocols

Thank you again for your submission to our journal. We hope that our editorial process has been constructive thus far, and we welcome your feedback at any time. Please don't hesitate to contact us if you have any questions or comments.

Sincerely,

Roli Roberts

Roland Roberts, PhD

Senior Editor

PLOS Biology

rroberts@plos.org

------------------------------------

REVIEWERS' COMMENTS:

Reviewer #1:

[identifies himself as Ulf Toelch]

Priboi et al. present in their manuscript "Researchers' perspectives on preregistration in animal research" a valuable survey results on the perception of preregistration of animal researchers in Switzerland. The presented data is an important contribution that will inform policy not only in Switzerland and beyond. To increase the impact, the authors may consider my comments below.

The study is complex including a preregistration and many supplemental tables. I apologise to the authors that I could not assess the whole material. Time given for review times at PLOS Biology are extremely short and reviewers who contribute their time for free are constantly harassed with emails about the status of their review. In the short time allowed, I was not able to do an in depth comparison of the preregistration and the actual manuscript. This already shows that for preregistrations to become useful also publisher and editors need to allow for slower but more rigorous science.

Value of preregistration

The authors start with the slight overstatement that preregistration is impactful and at the same time acknowledge that evidence backing this claim is not conclusive yet (ll62/63). Animal researchers already preregister their study with regulatory authorities even though this may not be as exhaustive as a formal preregistration and not include all the details. It is unclear in particular in the discussion what a formal preregistration would add to the animal permits. With the negative attitudes displayed by researchers how does this connect to the thematic analysis what researchers want and how realistic are these demands? Overall, the manuscript would benefit from a less enthusiastic description of preregistration as the panacea to problems in animal research.

Comparison of participants and non participants.

The authors compare study participants to non participants. This is done via t-tests and an absence of significance is interpreted as similarity between groups. There is good evidence that such an approach is not optimal (Table 1 fallacy). If the authors are interested in confounders or selection bias, the three comparions described here are insufficient to conclude that study populations were similar in the sense that results here will generalise to the general population of study directors. This should be acknowledged in the discussion. The authors may want to consider removing p-values in the comparison of the two groups.

Psychological scales

The scales are analysed in much detail. The reader is, however, lost in numbers. A Figure summarising some of the scale results would be beneficial to get a better grasp of magnitude of effects. Also there are multiple comparisons and only on some scales there are differences for example for gender. This leads to meaningless comparisons such as: "Follow-up pairwise comparisons (S7 Table) revealed that participants who preferred not to state their gender reported significantly less positive attitudes and lower motivations to preregister than participants identifying as female or male;" (ll409-411) This is based on a comparison of 14 particpants vs the rest of the population. I doubt that based on this can claim that these differences exist. The reported comparisons should be evaluated carefully by the authors.

That said, in the current form I feel it is difficult to see the overall picture. The results are presented in a way that makes it hard to grasp what the data looks like. Maybe supplementary tables contain this information but I was not able to assess these (see comment above). An additional Figure and a more selective presentation of the psychological scale results could facilitate a better understanding of the material provided.

Reviewer #2:

[identifies himself as Andrew H. Tyner]

In "Researchers' perspectives on preregistration in animal research," the authors adapt a questionnaire from Spitzer and Mueller (2023) to explore attitudes, norms, and experiences with preregistration among a sample of Swiss study directors working in animal research. The authors present their data in rich detail through psychometric analyses of closed-ended items and a thoroughgoing exploration of themes emerging from the open-ended items. I concur with the authors on the importance of gaining a deeper understanding of researchers' experiences with preregistration and I commend them for their impressively high response rate and the care and detail of their analyses. Below I offer a set of suggestions to improve the presentation of the research and extract more meaning out of these data.

Major Comments

1. The authors rely heavily on significance testing, particularly in Aim 3, to the exclusion of substantive interpretations of their findings and descriptively meaningful results. My main takeaway from Aim 3 is that many of these variables have statistically significant associations, many others do not, and I'm not left with any clearer understanding of what these findings tell us about challenges, opportunities, or understandings of preregistration in their sample. The supplemental materials aren't much help either - for example, in the 'Field of research' analyses the authors refer readers to Table S8, but this table (like Tables S7 and S9) only shows results on the transformed scales. What am I to make of the fact that basic and experimental medical researchers have a mean of -0.47 on the attitudes scale, while basic biological researchers have a mean of -0.70 and general biologists have a mean of -0.12? I understand that the authors are making use of the scales they developed in Aim 2, but for a general audience the authors need to be translating at least some of these results into substantively meaningful terms. I would encourage the authors to make greater use of visualizations in this manuscript to highlight some of the key relationships they see emerging in their data. In some situations these visualizations might make use of the underlying items to complement the presentation of scales.

2. As a related concern, the MANCOVA models presented in Aim 3 need a more careful presentation and justification. To start with, the authors should remove any language referring to effects (e.g. "A MANCOVA was conducted to examine the effects of preregistration experience, animal research experience, gender, field of research, and organization of employment on the eight Preregistration Scales and Subscales" [371-373]). These are not causal models and the data they've collected do not lend themselves to a causal analysis or interpretation. What's more, the setup of the models doesn't follow an intuitive causal ordering - they've set up the preregistration scales as the outcome variables and included preregistration experience as a predictor. The picture is undoubtedly more complicated than attitudes/norms affect behaviors, but a model where the behaviors are a primary predictor for the psychometric outcomes seems even more questionable. At the very least we'd think of behavior/experience and attitudes/norms as mutually influencing each other. Again, all of this language of causal ordering and effects is misplaced given the structure of these data - the real takeaway is that many of these predictors and constructs are statistically related. But, if the authors are going to present their findings in causal terms and set up models with predictors and outcomes, it should be done more carefully.

3. The presentation of the open-ended results in Aims 4 and 5 is impressively thorough. Still, I wonder whether these results could be better summarized or organized to distill key insights and takeaways for the reader. As it stands, these sections often read like the authors are just relaying all of the open-ended responses they received. The section on Knowledge Barriers stands out as an example: "Many participants stated that they were not aware that preregistration even existed. Some were unfamiliar with the procedure, describing a lack of guidance or uncertainty about the steps involved and the appropriate platforms for their studies. Others expressed general skepticism about the potential benefits and overall impact of preregistration in science" (471-474). Help the reader navigate these findings - what percentage of researchers expressed these views? Does that vary by relevant background characteristics? What do we learn from this that we didn't get from the close-ended items? And then a few lines later in the next section: "Often, participants working in exploratory or fundamental research questioned the feasibility of preregistration for their studies" (477-478). What does often mean? What does questioning the feasibility mean? And "However, while some participants recognized these advantages, many saw no benefits at all, or found it appropriate only for confirmatory studies" (493-495) - why such imprecision in reporting? Who holds these beliefs and in what proportions?

4. Finally, I was not able to access this manuscript's data since the provided DOI did not resolve (https://doi.org/10.48620/91802). The authors mention that the files are currently under review by the team managing University of Bern's repository, so perhaps that's the reason. All the same, reviewers should have an opportunity to reproduce the authors' analyses as a matter of course.

Minor comments

5. The authors explain their revised analytical approach in item 3 of Table S1. They write that they switched from simple and multiple linear regressions to a MANCOVA approach because of multi-category predictors. I agree with the authors that MANCOVA is an advisable approach here but not for the reasons the authors offer. The advantage of MANOVA/MANCOVA (and multivariate analyses more generally) is the simultaneous estimation of models with distinct but correlated outcome variables. It doesn't have anything to do with multi-category predictors. You can fit multiple linear regressions with multi-category predictions without a problem - like binary predictors, one level becomes the reference and the results of the other categories are interpreted in light of that reference category.

6. "Preregistration is a promising tool for improving transparency and quality of research, and is arguably among the most impactful Open Science practices" (lines 56-58). I'd soften this language to, "...and arguably has the potential to be among the most impactful…" The evidence base on preregistration is not strong enough yet to justify the statement as written (as the authors point out elsewhere in the manuscript).

7. "The inability to replicate published results…have questioned…" (lines 68-69". Rephrase to, "...have led to questions…"

8. "...these questionable research practices can slow down scientific progress, waist animal lives for inconclusive results…" (lines 78-79). Should be "...waste animal lives…"

9. "The values for non-participants are FSVO-provided data, whereas those for participants are based on survey responses. This distinction arises because FSVO collected gender data using only two categories (female, male), while our survey allowed for four options (female, male, other, and prefer not to say)" (lines 295-297). I'd recommend that the FSVO-provided data be used for both groups in the Table 1 reporting. Otherwise the data aren't directly comparable.

10. "Taken together, these results suggest that participants and non-participants were comparable in terms of age, gender distribution, and animal research experience" (lines 318-320). I'd recommend a more constrained interpretation here to avoid conflating absence of evidence with evidence of absence. Perhaps something like: "Altogether we do not find statistically significant differences between participants and non-participants in terms of age, gender distribution, or animal research experience."

11. "Similarly, a further survey conducted in the Netherland…" (566). Should be "...in the Netherlands…"

12. "We can only speculate that these participants may have had negative experiences with preregistration and/or may have high privacy concerns that leaves them in general reluctant to disclose information, being it research ideas or private information such as their gender" (615-618). Even as the authors acknowledge that this is speculative, I find this explanation nonsensical. The reasons participants have for not disclosing their gender identities are the same reasons they don't preregister? This is implausible and frankly needless - I see it as a consequence of the over-reliance on significance testing. The authors had no reason to expect this significant result in the first place and consequently offer a haphazard interpretation of it - this is a recipe for capitalizing on noise.

13. "This delimitation could help reduce research waste by avoiding repetition and support the refinement of future studies, contributing to overall less animals use" (650-652). I had trouble following this sentence. Consider a rephrase for clarity.

14. "Second, despite being introduced to the definition of preregistration at the beginning of the survey, some participants may not have read or fully understood the concept...This is supported by requests received after survey completion from three participants who asked to have their data deleted upon realizing that they had confused the two concepts" (708-715). Kudos to the authors for this level of transparency. I agree that it's a concern (if three people wrote in with this request there's a good chance that others were also confused), but there's not much to be done about it now. So mostly I just want to thank the authors for the transparent reporting. I think it strengthens the overall credibility of the manuscript and demonstrates exemplary reporting.

Best regards,

Andrew Tyner

Principal Research Scientist

Center for Open Science

andrewtyner@cos.io

Reviewer #3:

[identifies himself as Olmo van den Akker]

Please find my review in the attached file, and good luck with any revisions!

[unformatted version; see attachment for formatted version]:

Review of the manuscript titled “Researchers‘ perspectives on preregistration in animal research” submitted to PLOS Biology

Reviewer: Olmo van den Akker

In this manuscript, the authors surveyed study directors of animal experiments in Switzerland to elicit their experiences with and perceptions of the practice of preregistration in animal research. While the survey responses were heterogeneous, a picture emerged of study directors that have limited knowledge and experience with preregistration, which possibly influenced the skeptical attitudes about the practice.

I appreciated the high level of transparency provided in this manuscript, especially the way that the authors show how their questionnaire follows from the questionnaire used by Spitzer and Müller, and that the authors also present the available demographic characteristics of the non-participants. Below you can find a list of major and minor points for improvement.

Major points for improvement:

1. The DOI provided for accessing the data (https://doi.org/10.48620/91802) is currently inaccessible, presumably because it remains under review. Ensuring public data availability is essential for assessing validity, enabling re-analysis, and facilitating potential inclusion in systematic reviews.

2. The description of the qualitative analysis is minimal. The statement that “recurring themes were identified” does not provide enough detail to assess the analytical rigor. More information on coding procedures, number of coders, consensus strategies, and links between data excerpts and reported findings is needed.

3. The authors preregistered using Cronbach’s α with a cut-off value of 0.70, yet report McDonald’s ω instead and retain (sub)scales with ω < 0.70. These deviations are not included in the preregistration deviation table. The rationale for these changes, and their implications for reliability and validity, should be explained.

4. Multiple statistical tests are conducted without correction for multiple comparisons. The authors should either justify this decision or apply a correction. In line with this, more emphasis on effect sizes is recommended, especially where statistically significant results correspond to very small effect sizes (e.g., η² = 0.01–0.02), as it is unclear whether such effects are meaningful.

5. The authors carry out many statistical tests but do not correct for multiple testing. The authors should convincingly explain why they did not do so or implement a correction for multiple testing that ensures the findings are not merely based on randomness. What could help here is to focus more on effect sizes. These are more informative, especially since there are many cases where associations are statistically significant but effect sizes are low (eta-squared = 0.01 – 0.02). Can these really be interpreted as substantial effects?

6. Some of the obstacles perceived by the participants can be easily rebutted (e.g. that deviations are discouraged or even forbidden). I would like to see the authors do this in their discussion section as it is quite worrisome that such misconceptions about preregistration remain and seem to influence uptake and attitudes.

7. The recommendations offered in the conclusion (infrastructure, education, incentive systems) are very broad. More concrete and actionable suggestions, perhaps tailored to different stakeholders would improve the manuscript’s utility. Example: “Providing the necessary infrastructure, appropriate educational options and a well-designed incentive system might thus help foster the use of preregistration in the animal research community.”

8. To what extent are the results of participants who never heard of preregistration relevant? Shouldn’t they be left out given that they have no insight into the practice, or shouldn’t the sample be split into those who had at least heard of the practice and those who had not?

Minor points for improvement:

1. The introduction is quite comprehensive but the authors should acknowledge that there is also research highlighting that preregistration is currently not as effective as it could be. For example, there are observational studies that show that undisclosed deviations are common and that the positive effects of preregistration are not consistently found across studies.

2. A link to more information about Animex-ch would be helpful.

3. “Additionally, one more reminder was issued at the institutional level by the Animal Welfare Officers (AWO). At the time of the study, 49 institutions in Switzerland had designated AWOs” Does this mean that not all directors received an additional reminder? If so, could this have introduced bias into the sample?

4. It is not entirely clear why some (sets of) questions are labeled under psychosocial constructs and others are not. It can be argued that ‘obstacles’ do not really tap into participants’ psychology, while it can equivalently be argued that the perceptions of barriers to preregistration do tap into psychology. It seems the main difference is the way they are measured so maybe the description should focus on that. The authors could be clearer about this and maybe even disregard the term “psychosocial constructs” altogether.

5. The response rate of 30.2% is way higher than what the authors (and myself) expected. Why do the authors think this is the case? Does this have any ramifications for the interpretation of the results?

6. The authors mention in the preregistration that they would first assess the appropriateness of doing a PCA, but they don’t mention the Kaiser-Meyer-Olkin (KMO) measure of sampling adequacy and Bartlett's test of sphericity in the manuscript. It would be good to state whether these ‘tests’ were indeed carried out.

7. The authors use a MANCOVA framework instead of the preregistered linear regressions (and disclose this in the preregistration deviation table). Because these regressions were planned as the most important analyses in the study, I would encourage the authors to do these regressions regardless and present the results in a supplement. I also think it would be good if the authors would discuss the influence this deviation had on statistical power.

8. Why did the authors more comprehensively present Wilks’ Lambda than Pillai’s Trace when presenting the results? A sentence in the manuscript explaining this would be useful.

9. The process for removing items with “poor psychometric properties” should be described in more detail so readers know which items were removed and why.

10. How do the authors reconcile contradictory survey results? For example, that preregistration is seen by some as more time-consuming and by others as less time-consuming, or that preregistration is seen by some as conducive to collaboration and by others as hampering it.

11. “This study represents the first attempt to evaluate the researchers’ experience with, and perspectives on, preregistration in animal research” I don’t think this claim can be made given that the authors cite some references that also do this (e.g., Wieschowski et al., 2020).

12. “However, the gender difference could also reflect that men tend to overestimate their knowledge more often than women, suggesting that women lower ratings may indicate more accurate or honest self-evaluations rather than a true lack of capacity.” This claim requires references

13. Speculation about the negative experiences or privacy considerations of participants who did not disclose their gender seems excessive, particularly given the small number of respondents in this category, and should be moderated.

14. The claim that the sample is representative of all animal scientists in Switzerland is not supported, since study directors constitute a specific subgroup. I think this statement should be reconsidered.

15. The limitation on page 34 is outlined well but I would like to see some more discussion of how this specific limitation could have biased the results.

Attachment Submitted filename: Review Wurbel.docx

# Author comment: Author response to Decision Letter 2

**DOI:** 10.1371/journal.pbio.3003511.r003

14 Apr 2026

Attachment Submitted filename: Response_to_Reviewers.docx

# Peer review: Decision Letter 2

**DOI:** 10.1371/journal.pbio.3003511.r004

**Authors:** Roland G Roberts

28 May 2026

Dear Dr Würbel,

Thank you for your patience while we considered your revised manuscript "Researchers‘ perspectives on preregistration in animal research" for publication as a Meta-Research Article at PLOS Biology. This revised version of your manuscript has been evaluated by the PLOS Biology editors, the Academic Editor and two of the original reviewers.

Based on the reviews, we are likely to accept this manuscript for publication, provided you satisfactorily address the remaining points raised by the reviewers and the following data and other policy-related requests.

IMPORTANT - please attend to the following:

a) We normally like Titles to contain an active verb, plus some idea of the results, so please could you change it to "A survey of researchers' attitudes to preregistration in animal research reveals multiple perceived barriers to adoption"

b) Please address the remaining concerns raised by the reviewers.

c) Please address my Data Policy requests below; specifically, we need you to supply the numerical values underlying Figs 1, 2ABCDE, 4, either as a supplementary data file or as a permanent DOI’d deposition. I note that you already have an associated BORIS deposition (https://doi.org/10.48620/91802); please could you confirm whether the data and code in this deposition are sufficient to recreate the Figures? (also note the code issues raised by reviewer #2)

d) Please cite the location of the data clearly in all relevant main and supplementary Figure legends, e.g. “The data and code required to generate this Figure can be found in "https://doi.org/10.48620/91802".

As you address these items, please take this last chance to review your reference list to ensure that it is complete and correct. If you have cited papers that have been retracted, please include the rationale for doing so in the manuscript text, or remove these references and replace them with relevant current references. Any changes to the reference list should be mentioned in the cover letter that accompanies your revised manuscript.

In addition to these revisions, you may need to complete some formatting changes, which you will receive in a follow up email. A member of our team will be in touch with a set of requests shortly. If you do not receive a separate email within a few days, please assume that checks have been completed, and no additional changes are required.

We expect to receive your revised manuscript within two weeks.

To submit your revision, please go to https://www.editorialmanager.com/pbiology/ and log in as an Author. Click the link labelled 'Submissions Needing Revision' to find your submission record. Your revised submission must include the following:

- a cover letter that should detail your responses to any editorial requests, if applicable, and whether changes have been made to the reference list

- a Response to Reviewers file that provides a detailed response to the reviewers' comments (if applicable, if not applicable please do not delete your existing 'Response to Reviewers' file.)

- a track-changes file indicating any changes that you have made to the manuscript.

NOTE: If Supporting Information files are included with your article, note that these are not copyedited and will be published as they are submitted. Please ensure that these files are legible and of high quality (at least 300 dpi) in an easily accessible file format. For this reason, please be aware that any references listed in an SI file will not be indexed. For more information, see our Supporting Information guidelines:

https://journals.plos.org/plosbiology/s/supporting-information

*Published Peer Review History*

Please note that you may have the opportunity to make the peer review history publicly available. The record will include editor decision letters (with reviews) and your responses to reviewer comments. If eligible, we will contact you to opt in or out. Please see here for more details:

https://plos.org/published-peer-review-history/

*Press*

Should you, your institution's press office or the journal office choose to press release your paper, please ensure you have opted out of Early Article Posting on the submission form. We ask that you notify us as soon as possible if you or your institution is planning to press release the article.

*Protocols deposition*

To enhance the reproducibility of your results, we recommend that if applicable you deposit your laboratory protocols in protocols.io, where a protocol can be assigned its own identifier (DOI) such that it can be cited independently in the future. Additionally, PLOS ONE offers an option for publishing peer-reviewed Lab Protocol articles, which describe protocols hosted on protocols.io. Read more information on sharing protocols at https://plos.org/protocols?utm_medium=editorial-email&utm_source=authorletters&utm_campaign=protocols

Please do not hesitate to contact me should you have any questions.

Sincerely,

Roli Roberts

Roland Roberts, PhD

Senior Editor

rroberts@plos.org

PLOS Biology

------------------------------------------------------------------------

DATA POLICY:

You may be aware of the PLOS Data Policy, which requires that all data be made available without restriction: http://journals.plos.org/plosbiology/s/data-availability. For more information, please also see this editorial: http://dx.doi.org/10.1371/journal.pbio.1001797

Note that we do not require all raw data. Rather, we ask that all individual quantitative observations that underlie the data summarized in the figures and results of your paper be made available in one of the following forms:

1) Supplementary files (e.g., excel). Please ensure that all data files are uploaded as 'Supporting Information' and are invariably referred to (in the manuscript, figure legends, and the Description field when uploading your files) using the following format verbatim: S1 Data, S2 Data, etc. Multiple panels of a single or even several figures can be included as multiple sheets in one excel file that is saved using exactly the following convention: S1_Data.xlsx (using an underscore).

2) Deposition in a publicly available repository. Please also provide the accession code or a reviewer link so that we may view your data before publication.

Regardless of the method selected, please ensure that you provide the individual numerical values that underlie the summary data displayed in the following figure panels as they are essential for readers to assess your analysis and to reproduce it: Figs 1, 2ABCDE, 4. NOTE: the numerical data provided should include all replicates AND the way in which the plotted mean and errors were derived (it should not present only the mean/average values).

Please also ensure that figure legends in your manuscript include information on where the underlying data can be found, and ensure your supplemental data file/s has a legend.

Please ensure that your Data Statement in the submission system accurately describes where your data can be found.

------------------------------------------------------------------------

DATA NOT SHOWN?

- Please note that per journal policy, we do not allow the mention of "data not shown", "personal communication", "manuscript in preparation" or other references to data that is not publicly available or contained within this manuscript. Please either remove mention of these data or provide figures presenting the results and the data underlying the figure(s).

------------------------------------------------------------------------

REVIEWERS' COMMENTS:

Reviewer #1:

[identifies himself as Ulf Toelch]

The revised version of the manuscript "Researchers' perspectives on preregistration in animal research" by Probi et al. has improved substantially in clarity and is an excellent read with highly relevant analyses regarding the perception and uptake of preregistrations in animal research. Also data and associated meta data have been deposited in an accessible format in an open repository. Some minor points remain that the authors may want to address.

1.In agreement with reviewer #2, the text has been updated to give a less optimistic and now more nuanced view of preregistrations. What remains, however, is the first sentence of the abstract that, in prominent position, advocates preregistrations in manner that is in my opinion not yet backed up by the evidence. Even when adding "potentially" this still is a very strong statement. This is also the case in ll56/57. I again suggest to tone this down even further. Why the nitpicking on a single sentence? The paper will hopefully be used in policy discussions. These discussion will in my opinion not benefit from the strong language used in the paper. Most evidence only comes from related fields like psychology and clinical trials. Even here the evidence is mixed (e.g. outcome switching occurs despite preregistration in clinical trials, papers deviate in an undisclosed way from preregistrations in psychology). More and better evidence is needed to showcase under what circumstances preregistrations make sense and what expected benefits for the scientific system will be. The paper is an important contribution to inform these discussions. A language that signals an openness to these discussion could be helpful. Please take this only as mild nudging and not fundamental criticism of the paper at large.

2.Ll 232 the conventional threshold for equivalence tests needs a reference. I agree that the equivalence test used here is now better suited than a conventional t-test. In my opinion there is not even a need for a test. The need for a sample that is not influenced by a selection bias is not as strong as in a clinical trial. The authors also discuss the possibility of a selection bias.

3. The Figures now represent the underlying data much better. There is a small typo in the text referring to Figure 2 (grater->greater, line 437). L 434 what does modest mean here. You have in the methods indicated low medium and large effect sizes. Please refer to effect sizes in this terminology. Effect sizes were largely small and some were medium. I also do not understand the word consistency here. What would consistency of effect sizes across categories mean?

Reviewer #2:

[identifies himself as Andrew H. Tyner]

Thanks for the opportunity to re-review "Researchers' perspectives on preregistration in animal research". It's clear from the extensive revisions and detailed point-by-point responses that the authors thoughtfully considered all of the feedback they were provided and made sensible decisions on how to address it. I'm comfortable with this manuscript proceeding to publication. Below I outline my one major reservation; a handful of minor substantive suggestions; and a few remaining line-level suggestions. The line numbers I reference are for the version with tracked changes.

Major reservation

1. The reproducibility materials are now available at the link the authors provided, which is good to see. Still, I'm not able to reproduce their analyses using these materials. Part of this has to do with file names. In the readme, the data file is called "Preregistration_Study.xlsx", in the actual materials it's called "Preregistration_Study-anonymized_data", and in the R code it's called "PreReg_Study.xlsx". Additionally, "PreReg_Study.xlsx" appears to be the input of the cleaning script, the output of the cleaning script, and the input of the analysis script. I would strongly prefer to see a 'raw' data file that serves as the input to the cleaning script and then an analytic data file that's both the output of the cleaning script and the input of the analysis script. Also, when I try to run the cleaning script by changing the name of the input file, it throws an error at line 418 because it's being asked to drop columns that don't exist.

Minor suggestions

2. "In addition, we used a criterion based on the ratio of the first and second eigenvalue of the PCA, as well as omega hierarchical (ωH) from a bifactor model, to assess essential unidimensionality" (lines 281-283). What was the actual criterion? The authors allude it to without defining it.

3. "When necessary, items with poor psychometric properties (low factor loadings and/or low corrected item-total correlations) and weak conceptual alignment were removed, resulting in improved internal consistency and construct validity of each scale" (lines 424-426). Which items were removed? This is the Results section, so they should just report it directly.

4. "Interestingly, our analysis also revealed differences by gender and organization of employment...may indicate more accurate or honest self-evaluations rather than a true lack of capacity" (lines 813-822). On one hand, nothing they include in this paragraph is inaccurate. On the other hand, it seems slightly misleading to highlight these single instances of statistical differences without recognizing that across *most* constructs there were not statistically significant differences for gender or employment. The absence of evidence for differences is, for me, the main takeaway here.

5. "This might be due to the less competitive nature resulting in less fear of being scooped in General Biology compared the more competitive fields of basic and applied biomedical research" (lines 834-837). I am not in the relevant disciplines, so for a general audience I would suggest more context on what makes basic and applied biomedical research more competitive than General Biology.

6. "Although preregistration is arguably more straightforward in the case of a confirmatory study with a clearly defined hypothesis, it is another common misunderstanding that preregistration cannot be used for exploratory research. Again, this perceived barrier might be addressed by educating animal researchers about existing flexible preregistration templates (e.g., AsPredicted, OSF) that support the preregistration of specific types of research" (lines 899-908). I think this slightly misstates the problem. The question isn't *whether* preregistration of non-confirmatory research is possible; it's *why* researchers should do so. The solution here is to foreground the value proposition of preregistration in non-confirmatory research rather than merely showing that it's possible.

7. The particularly high administrative burdens associated with animal research in Switzerland are alluded to a few times without being explicitly defined. For those without the contextual background, what makes the administrative requirements more stringent in Switzerland compared to peer countries? This deserves a paragraph unto itself in the Discussion since it's a factor that potentially limits the generalizability of their findings.

Line-level suggestions

8. "On average, participants exhibited rather unfavorable attitudes towards preregistration..." (lines 45-46). Perhaps "reported" rather than "exhibited" here.

9. "Our findings offer guidance on promising measures to enhance acceptance of preregistration among animal researchers, including raising awareness, offering education and training, and facilitating procedures" (lines 50-53). The ending of this sentence is strange. Facilitating which procedures?

10. "A report by the US National Institute of Health working group has recommended..." (lines 97-98). Which working group are they referring to here?

11. "associations of several predictors on the Preregistration Scales..." (line 297). Should probably be "associations of several predictors with the Preregistration Scales...".

12. "However, on all scales and subscales, participants ratings ranged from..." (lines 455-456). Should be "participants' ratings" instead.

# Author comment: Author response to Decision Letter 3

**DOI:** 10.1371/journal.pbio.3003511.r005

11 Jun 2026

Attachment Submitted filename: ResponseToReviwers.docx

# Editor report: Decision Letter 3

**DOI:** 10.1371/journal.pbio.3003511.r006

**Authors:** Roland G Roberts

15 Jun 2026

Dear Hanno,

Thank you for the submission of your revised Meta-Research Article "A survey of researchers' attitudes to preregistration in animal research reveals multiple perceived barriers to adoption" for publication in PLOS Biology. On behalf of my colleagues and the Academic Editor, Cilene Lino de Oliveira, I'm pleased to say that we can in principle accept your manuscript for publication, provided you address any remaining formatting and reporting issues. These will be detailed in an email you should receive within 2-3 business days from our colleagues in the journal operations team; no action is required from you until then. Please note that we will not be able to formally accept your manuscript and schedule it for publication until you have completed any requested changes.

Please take a minute to log into Editorial Manager at http://www.editorialmanager.com/pbiology/, click the "Update My Information" link at the top of the page, and update your user information to ensure an efficient production process.

PRESS: We frequently collaborate with press offices. If your institution or institutions have a press office, please notify them about your upcoming paper at this point, to enable them to help maximise its impact. If the press office is planning to promote your findings, we would be grateful if they could coordinate with biologypress@plos.org. If you have previously opted in to the early version process, we ask that you notify us immediately of any press plans so that we may opt out on your behalf.

We also ask that you take this opportunity to read our Embargo Policy regarding the discussion, promotion and media coverage of work that is yet to be published by PLOS. As your manuscript is not yet published, it is bound by the conditions of our Embargo Policy. Please be aware that this policy is in place both to ensure that any press coverage of your article is fully substantiated and to provide a direct link between such coverage and the published work. For full details of our Embargo Policy, please visit http://www.plos.org/about/media-inquiries/embargo-policy/.

Thank you again for choosing PLOS Biology for publication and supporting Open Access publishing. We look forward to publishing your study.

Sincerely,

Roli

Roland G Roberts, PhD, PhD

Senior Editor

PLOS Biology

rroberts@plos.org
